# Macroevolutionary patterns in marine hermaphroditism

**DOI:** 10.1111/evo.14639

**Published:** 2022-10-13

**Authors:** George C. Jarvis, Craig R. White, Dustin J. Marshall

**Affiliations:** ^1^ School of Biological Sciences/Centre for Geometric Biology Monash University Melbourne VIC 3800 Australia

**Keywords:** Biogeography, hermaphroditism, life history, local gamete competition

## Abstract

Most plants and many animals are hermaphroditic; whether the same forces are responsible for hermaphroditism in both groups is unclear. The well‐established drivers of hermaphroditism in plants (e.g., seed dispersal potential, pollination mode) have analogues in animals (e.g., larval dispersal potential, fertilization mode), allowing us to test the generality of the proposed drivers of hermaphroditism across both groups. Here, we test these theories for 1153 species of marine invertebrates, from three phyla. Species with either internal fertilization, restricted offspring dispersal, or small body sizes are more likely to be hermaphroditic than species that are external fertilizers, planktonic developers, or larger. Plants and animals show different biogeographical patterns, however: animals are less likely to be hermaphroditic at higher latitudes—the opposite to the trend in plants. Overall, our results suggest that similar forces, namely, competition among offspring or gametes, shape the evolution of hermaphroditism across plants and three invertebrate phyla.

Hermaphroditism, in which individuals produce both male and female gametes in their lifetime, occurs in almost all higher plants (95%) and around a third of animals (excluding insects [Avise 2011]). The forces that drive hermaphroditism in plants are well‐resolved (Bawa 1980; Renner and Ricklefs 1995; Sakai and Weller 1999; de Jong and Klinkhamer 2005; Walas et al. 2018), but it is unclear if the same forces are responsible for hermaphroditism in animals (Williams 1975). If hermaphroditism is driven by similar forces in these two evolutionary disparate groups, we can start to develop more general and robust predictions for how hermaphroditism should (co)vary with life history and ecology in multicellular organisms.

Most of our understanding of hermaphroditism comes from plants. Hermaphrodites are thought to be more likely to self‐fertilize and suffer negative consequences of inbreeding depression (Charlesworth and Charlesworth 1981; Lloyd 1982). However, when opportunities for mating are limited (e.g., at low population densities), the costs of selfing may be outweighed by the benefits of reproductive assurance (Darwin 1876; Tomlinson 1966; Ghiselin 1969). When the relationship between resource allocation and fitness decelerates with further investment in any one sex, hermaphroditism should be favored over separate sexes (i.e., dioecy in plants, gonochorism in animals [Charnov et al. 1976]). The conditions associated with decelerating fitness functions (and hence the evolution of hermaphroditism) in plants are as follows: (1) limited seed dispersal, resulting in sibling competition (Charnov et al. 1976; Vamosi et al. 2007); (2) animal pollination, which is generally more efficient than wind/water pollination, and thus increases competition among related pollen grains; and (3) small adult size, because smaller plants may be more pollen limited (Charnov 1982; Klinkhamer et al. 1997).

Theoretical predictions about how hermaphroditism should (co)vary with life history and ecology in plants are well supported. Hermaphroditism is more common in temperate climates (Bawa and Opler 1975; Vamosi et al. 2003; Wang et al. 2020) and higher latitudes (Baker and Cox 1984; Moeller et al. 2017), where opportunities for mating may be more limited than at lower latitu (da Cunha et al. 2022). Seeds of hermaphrodites are typically dispersed by wind or water over short distances, whereas seeds of dioecious plants are often animal‐dispersed further away from their parents (Bawa 1980; Sakai and Weller 1999; Vamosi et al. 2003). Most hermaphrodites are pollinated by specialist insects with more efficient pollination patterns, relative to wind and water pollination more common to dioecious species (Beach 1981; Lloyd 1982; Charlesworth 1993; Renner and Ricklefs 1995; Vamosi et al. 2003). Last, hermaphroditic plants are typically smaller than dioecious species (Bawa 1980; Sakai and Weller 1999; Wang et al. 2020), either to avoid inbreeding at larger sizes (Baker 1955) or because stronger pollen limitation in smaller plants relative to larger plants favors the reallocation of resources from female to male function as a hermaphrodite when small (Charnov 1982; Andersson 1988; Lawrence 1993; Eppley and Pannell 2007). Overall, these macroevolutionary patterns are strongly suggestive that theory has done a good job of identifying the drivers of hermaphroditism. One way of testing whether the proposed theoretical drivers of hermaphroditism in plants are robust and universal is to test for analogous patterns in animals. To date, however, hermaphroditism in animals has been studied from a very different perspective.

Much of the theory developed for the evolution of hermaphroditism in animals has focused on the social drivers of sex change in sequential hermaphrodites (Ghiselin 1969; Warner et al. 1975; Munday et al. 2006). For example, bluehead wrasse, *Thalassoma bifasciatum*, form groups consisting of a dominant male and several smaller females—if the male is removed, the next largest female becomes the dominant male (Warner and Swearer 1991). The social drivers of sequential hermaphroditism in fishes are better understood than the divers of simultaneous hermaphroditism in other animals (Avise and Mank 2009). However, the drivers of sequential hermaphroditism are only likely to apply in species with clear social structures—more general theories about the evolution of simultaneous hermaphroditism in animals are less well tested (but see Reid 1990; Hart et al. 1997; Jarne and Auld 2006; Eppley and Jesson 2008; Iyer and Roughgarden 2008; Schärer 2009; Erisman et al. 2013; Leonard 2013a, 2013b; Pla et al. 2021). Nevertheless, there are some elements of theories developed for plants that conceptually overlap and may apply to animals: namely, theory focused on local resource and local gamete competition. Below, we discuss the potential life‐history and ecological correlates of simultaneous hermaphroditism (hereafter referred to as “hermaphroditism”) in animals as predicted by theory developed (mostly) for plants.

## OFFSPRING DISPERSAL POTENTIAL

In plants, low seed dispersal favors reallocation of resources from female to male function as a hermaphrodite (Charnov et al. 1976; Vamosi et al. 2007). The degree to which offspring dispersal potential covaries with hermaphroditism in other groups remains unclear. In marine organisms, species with planktonic larval development disperse offspring much further than species that do not (Lester et al. 2007). For those species with a planktonic larval stage, larvae that feed during development generally spend much more time in the plankton for a given temperature regime (Marshall et al. 2018), have greater dispersal (Vance 1973; Jablonski and Lutz 1983; Strathmann 1985; Shanks et al. 2003; Álvarez‐Noriega et al. 2020; but see Ewers‐Saucedo and Pappalardo 2019), and higher population connectivity (Olsen et al. 2020) than species with planktonic nonfeeding larvae. If larval dispersal affects local resource competition among offspring, it is reasonable to expect hermaphroditism to be most common in marine invertebrates with aplanktonic development, least common in species with feeding larvae, and intermediate in species with nonfeeding larvae (Heath 1979). There are indications this is the case—comparative tests in specific groups support this idea (Strathmann et al. 1984; Rouse and Fitzhugh 1994; Hart et al. 1997; Schärer 2009), allowing us to test whether these patterns exist more generally across taxa.

## FERTILIZATION MODE

Fertilization mode (i.e., whether the fertilization of eggs occurs inside the body or outside in an aquatic medium) has some analogous features with pollination mode. Just as animal pollination favors hermaphroditism in plants as a means of reducing gamete competition, internal fertilization in animals may also favor hermaphroditism (Charnov 1982). Fertilization tends to be more efficient in internal fertilizers (Levitan and Petersen 1995), so male fitness may be a diminishing function of sperm production (Charnov 1982). Furthermore, female fitness may be a diminishing function of egg production in internal fertilizers if retained eggs are more likely to compete for fertilization than broadcasted eggs of external fertilizers (Henshaw et al. 2014). Both scenarios—increased local gamete competition within either sperm or eggs—should favor hermaphroditism in internal fertilizers. Finally, internal fertilization is more associated with restricted offspring dispersal in some groups (Monro and Marshall 2015), so hermaphroditism should be favored in this group for multiple reasons. Hermaphroditism is indeed associated with internal fertilization in some specific clades (Strathmann and Strathmann 1982; Rouse and Fitzhugh 1994; Hart et al. 1997; Kupriyanova et al. 2001), but whether these patterns exist across a broader range of clades is largely unknown.

## BODY SIZE

Smaller species should be more likely to be hermaphroditic (Charnov 1982; Klinkhamer et al. 1997; Munday et al. 2006). Body size and hermaphroditism may have coevolved for different reasons in plants: (1) selection for dioecy at larger sizes because larger plants are more likely to self‐fertilize than smaller plants, and thus experience stronger selection for reproductive modes that prevent selfing; (Baker 1955; Maynard Smith 1978; de Jong and Klinkhamer 2005) and (2) selection for hermaphroditism at smaller sizes, because small plants are more pollen limited than large plants, which increases competition among related ovules for fertilization (Andersson 1988; Lawrence 1993; Eppley and Pannell 2007) and favors the reallocation of resources from female to male function as a hermaphrodite when small (Charnov 1982). Analogous theory in animals makes similar predictions—external fertilizers experience more sperm limitation when small, so smaller species should be hermaphrodites with internal fertilization, whereas larger species should be gonochoristic with external fertilization (Henshaw et al. 2014). Despite clear predictions, and longstanding interest in the covariance between body size and hermaphroditism in animals (Strathmann and Strathmann 1982; Hendler and Littman 1986; Rouse and Fitzhugh 1994; Hart et al. 1997; Kupriyanova et al. 2001), it has not been tested systematically.

## BIOGEOGRAPHY

Theory from plants suggests two reasons why we might expect hermaphroditism to covary with latitude in animals. First, if mating opportunities are rarer at the poles than at the tropics (as they are in plants), hermaphroditism may be favored at higher latitudes over lower latitudes. Second, dispersal potential and fertilization mode covary with latitude in marine invertebrates: aplanktonic development (Thorson 1936, 1950; Mileikovsky 1971; Laptikhovsky 2006; Marshall et al. 2012) and external fertilization (Monro and Marshall 2015) are more prevalent at higher latitudes than lower latitudes. Thus, the covariance of hermaphroditism with latitude may arise because of latitudinal patterns in these other drivers of hermaphroditism. Studies of biogeographical patterns in hermaphroditism in animals beyond specific clades (Longhurst 1955; Baird et al. 2009; Mathers et al. 2013; Oyarzún et al. 2020; Pla et al. 2021) remain rare.

Here, we test whether the well‐established theoretical drivers of hermaphroditism in plants also apply to animals. We formally test the (co)variation of hermaphroditism with larval developmental mode, fertilization mode, body size, and latitude across nearly 1200 marine invertebrates spanning three phyla (Molluscs, Annelids, and Echinoderms).

## Methods

### DATA COLLECTION AND CLASSIFICATION OF LIFE‐HISTORY MODES

We compiled data for reproductive mode, larval developmental mode, fertilization mode, geographic location (latitude), and adult size for 1153 species of marine annelids (328 spp.), echinoderms (386 spp.), and molluscs (439 spp.). Many of the species in our dataset came from previously published meta‐analyses on various marine invertebrate life‐history traits (e.g., Marshall et al. 2012; Monro and Marshall 2015), supplemented with additional data on hermaphroditism from the literature.

Unfortunately, we could not follow a formal Preferred Reporting Items for Systematic Reviews and Meta‐Analyses (PRISMA) approach (Page et al. 2021) because data for life history, latitude, and body size are rarely reported in a single source. Instead, once we found out if a species in our dataset was gonochoristic or hermaphroditic, we then searched for more information on the life history and ecology of that species. Information was collected from studies from ISI Web of Science (http://www.webofknowledge.com/WOS) and Google Scholar (https://scholar.google.com/) based on the following search terms: “[*Genus species*]” together with terms “fertiliz*,” “fertilizat* mode,” “development*,” “developmental mode,” “larva*,” “planktotroph*,” “lecithotroph*,” “direct develop*,” “weight,” “size,” “adult size,” “mass,” “adult mass,” or “latitud*.” Within those selected articles, we also explored relevant citations to identify as many studies as possible.

We classed species as simultaneous hermaphrodites if individuals were reported as having both male and female sex organs at the time of collection, and gonochoristic if individuals were reported as having either male or female sex organs at the time of collection. For most of the species in our dataset, we found no evidence for intraspecific variation in reproductive mode. However, in studies where hermaphroditism was reported as rare, or when a single hermaphrodite was found in a primarily gonochoristic population (e.g., Patent 1970; Komatsu and Oguro 1972; Campodónico et al. 2004), we classified the species as gonochoristic. When individuals were reported as sequential hermaphrodites (e.g., Crump and Emson 1978; Hendler 1979), or when sequential hermaphroditism could be inferred from taxonomy (e.g., all known species of gastropods in the genus, *Crepidula* are sequential hermaphrodites [Coe 1948]), we classified the species as a sequential hermaphrodite. Most (77%) of the hermaphrodites in our dataset are simultaneous (cf. sequential) hermaphrodites but we acknowledge that sequential hermaphroditism is likely underestimated in our dataset. Sequential hermaphrodites are either male or female at any instant in time, so longer term studies are required to determine whether apparently gonochoristic/dioecious individuals are in fact gonochoristic/dioecious or sequentially hermaphroditic—such studies are relatively rare. To test whether hermaphroditism type influenced our results, we analyzed our data with gonochores and either simultaneous or sequential hermaphrodites only and compared those results to our analyses where both types of hermaphroditism were included.

We recorded each species’ mode of larval development, classified into one of three categories (sensu Marshall et al. 2012): (1) aplanktonic, for species that are brooded within the adult or develop in benthic egg masses, lack a free‐living planktonic embryo/larval stage, and emerge as juveniles; (2) planktonic nonfeeding, for species with pelagic larvae that do not require exogenous resources to complete development; and (3) planktonic feeding, for species with pelagic larvae that must feed to complete development.

We classified fertilization mode as external if eggs were reported as being fertilized outside the body of the female, and internal if eggs were reported as being fertilized within the body (sensu Monro and Marshall 2015). Under this classification, sperm casters (i.e., species that release sperm into the sea but retain eggs internally) and species with true copulation or pseudocopulation were considered internal fertilizers. Unfortunately, we could not test for patterns in hermaphroditism among different types of internal fertilization—this level of detail is not often reported, either because it is not appreciated for its ecological consequences, or because species’ exact mode of internal fertilization remains unresolved (Monro and Marshall 2015).

We collected data for adult mass (i.e., grams total wet weight of sexually mature individuals) and geographic coordinates for collection sites from the literature, supplemented with information from field guides and online databases (e.g., World Register of Marine Species: http://www.marinespecies.org/; SeaLifeBase: https://www.sealifebase.ca/). When adult sizes were reported as lengths or dry masses, we converted to wet weight based on conversion factors from Brey et al. (2010) and Robinson et al. (2010).

### STATISTICAL ANALYSES

We characterized how hermaphroditism covaries with developmental mode, fertilization mode, adult size, and latitude. Preliminary analyses suggested that hermaphroditism varies according to phyla (*χ* ^2^_phyla_ = 374.52, df = 2, *P* < 0.01), therefore we analyzed each phylum separately.

Within each phylum, some combinations of developmental and fertilization mode are exceedingly rare in nature and absent in our database (Table 1). Therefore, we only tested for interactions between different combinations of life‐history modes for which we had sufficient replication (Table S1). Specifically, annelids and molluscs with aplanktonic development are almost always internal fertilizers (86% and 98%, respectively), so we did not test covariances between hermaphroditism and fertilization mode for this developmental mode for these phyla. Furthermore, we did not examine the drivers of hermaphroditism in echinoderms with planktonic feeding development because all the species in this group are gonochores with external fertilization.

**Table 1:** Species records used in this study, organized by phylum, reproductive mode, fertilization mode, and developmental mode

			Developmental Mode		
Phylum	Reproductive Mode	Fertilization Mode	Aplanktonic	Planktonic Nonfeeding	Planktonic Feeding
Annelida	Hermaphroditic	Internal	5	29	4
		External		11	6
	Gonochoristic	Internal	20	72	27
		External	4	76	74
Echinodermata	Hermaphroditic	Internal	11	1	
		External	2	3	
	Gonochoristic	Internal	17	5	
		External	26	122	199
Mollusca	Hermaphroditic	Internal	33	38	185
		External	1	5	15
	Gonochoristic	Internal	28	6	33
		External		42	53

We analyzed our data with both phylogenetically controlled models and models with no phylogenetic control (which we will call “standard models”). Our standard models are better for describing the observed patterns in nature—these are the analyses that provide information about the probability of a species being a hermaphrodite given it has a certain trait or latitude. However, standard models inflate type I error rates if related species tend to resemble each other (Felsenstein 1985). Phylogenetically controlled analyses account for such nonindependence by estimating regression coefficients from a tree of phylogenetic associations among species (Harvey and Pagel 1991; Ives 2018). Both types of analyses are of interest, but for different reasons. For example, if one wishes to know whether smaller organisms are more likely to be hermaphroditic than larger organisms, then the standard models are most appropriate, but if one wishes to know whether there is an association between size and hermaphroditism over and above a shared evolutionary history among species, then the phylogenetically controlled analyses are most appropriate.

We used a multistep approach for our statistical analyses that largely followed the logic of Monro and Marshall (2015). First, we evaluated the overall significance of model effects using analysis of deviance tests based on *χ* ^2^ distributions (package “car” version 3.0‐10 [Fox and Weisberg 2019]), reducing models in which interactions were not significant (*P* > 0.05). Next, we evaluated the significance of regression coefficients (once the appropriate model had been selected from the previous step) using Wald tests (standard models) and bootstrapped 95% confidence intervals (phylogenetically controlled models), and compared coefficients between our two types of models to determine the role of phylogeny in driving the patterns that we observed. Below, we discuss model parameters and explain the differences between our two model types in more detail.

### STANDARD LOGISTIC REGRESSIONS

We first fit a standard logistic regression model (with reproductive mode as a binary response variable, developmental mode or fertilization mode as a categorical predictor, and latitude or natural‐log transformed body size as covariates) to all species for each phylum separately. The effects of latitude and body size were analyzed separately, due to insufficient representation of body sizes across latitudes in our dataset. We evaluated the effects of fertilization mode and its interaction with latitude or mass within developmental modes when such combinations were sufficiently replicated (Table S1).

**Table 2:** Standard logistic regressions testing the drivers of hermaphroditism in marine invertebrates. Main effects were evaluated using analysis of deviance tests. For full results and interactive effects of life history and ecology on hermaphroditism, see Tables S6 and S7. (*P* < 0.05 bolded)

Trait	Phylum	df	*χ* ^2^	*P*‐value
Developmental mode	Annelida	2	8.15	**0.02**
	Echinodermata	2	65.35	**<0.01**
	Mollusca	2	15.10	**<0.01**
	Annelida	1	12.13	**<0.01**
Fertilization mode	Echinodermata	1	42.79	**<0.01**
	Mollusca	1	138.57	**<0.01**
	Annelida	1	29.36	**<0.01**
Adult mass	Echinodermata	1	50.84	**<0.01**
	Mollusca	1	49.33	**<0.01**
	Annelida	1	5.74	**0.02**
Latitude	Echinodermata	1	0.32	0.57
	Mollusca	1	16.92	**<0.01**

### PHYLOGENETICALLY CONTROLLED LOGISTIC REGRESSIONS

For our phylogenetically controlled logistic regressions, we included the same model parameters as mentioned above, plus an added effect of phylogeny. Models were fitted and bootstrapped 1000 times using the “phyloglm” function in the “phylolm” package version 2.6.2 (Ho and Ané 2014), and model terms are significant when 95% confidence intervals from 1000 bootstrapped replicates do not overlap zero (Ives and Garland 2010). Following Ives and Garland (2010), we determined there to be phylogenetic signal (“*a*” parameter [Ives and Garland 2010]) in our response when 95% confidence intervals around *a* do not overlap <−4 (Tables S7–S9).

We extracted our phylogenies from the Open Tree of Life (Hinchliff et al. 2015) with the package “rotl” version 3.0.11 (Michonneau et al. 2016), and constructed phylogenetic trees with the package “phytools” version 0.7‐80 (Revell 2012 [see the Supporting Information for phylogenetic trees]). Branch lengths for the phylogeny were unknown, so we scaled branch lengths using Grafen's method (Grafen 1989) in the “ape” package version 5.6‐1 (Paradis and Schliep 2019).

To test whether phylogenetic resolution affected our results, we randomly resolved all polytomies in our phylogenetic trees 1000 times (we resolved polytomies with the “multi2di” function in the “ape” package and performed bootstrapping outside of the “phylolm” package) and reanalyzed our phylogenetic models, generating 1000 estimates for our model parameters based on phylogenies with randomly resolved polytomies. We then compared these estimates to our original models—if our original estimate fell within the range of estimates generated from models with randomly resolved polytomies, it demonstrates that our findings are robust to uncertainty in how polytomies are resolved in the phylogeny.

We completed all analyses in RStudio version 1.4.1717 (RStudio Team 2021). Figures were created using the “ggplot2” package version 3.3.5 (Wickham 2016), and all data and code for analyses are deposited in the Dryad Digital Repository: https://doi.org/10.5061/dryad.76hdr7t0v (Jarvis et al. 2022). Unless otherwise stated, the results for standard and phylogenetic logistic regressions were qualitatively identical.

## Results

Hermaphroditism was more common in molluscs in our database (63% of species), relative to annelids (17%) and echinoderms (4%). Internal fertilizers were more likely to be hermaphroditic than external fertilizers within each phylum (Tables 1 and 2; Fig. S1). Internal fertilizers were 15%, 30%, and 60% more likely to be hermaphroditic than external fertilizers for annelids, echinoderms, and molluscs, respectively (Table 3). Below, we explore patterns within phyla and developmental modes in more detail.

**Table 3:** Standard and phylogenetically controlled regressions testing the drivers of hermaphroditism in marine invertebrates. Intercepts were excluded for brevity, with estimates (on logit scale) for developmental mode and fertilization mode in reference to species with aplanktonic offspring development and internal fertilization, respectively. Estimates are considered significant based on Wald tests for standard regressions (*P* < 0.05 bolded), and when 95% confidence intervals from 1000 bootstrapped replicates do not overlap zero for phylogenetically controlled regressions (bolded [Ives and Garland 2010]). For full results of the interactive effects of life history and ecology on hermaphroditism, see Tables S8 and S9

		Standard Regressions		Phylogenetically Controlled Regressions
Trait	Phylum	Coefficient (SE)	*P*‐value	Coefficient ± SE (95% CI)
Developmental mode	Annelida			
	Planktonic nonfeeding	0.26 (0.52)	0.62	0.13 ± 0.42 (−0.52, 0.67)
	Planktonic feeding	–0.74 (0.59)	0.21	–0.71 ± 0.51 (−1.49, 0.03)
	Echinodermata			
	Planktonic nonfeeding	–2.46 (0.53)	**<0.01**	**–2.90 ± 0.77 (−4.75, −0.63)**
	Planktonic feeding	–19.78 (1.24 × 10^3^)	0.99	**–5.16 ± 1.48 (−19.21, −0.77)**
	Mollusca			
	Planktonic nonfeeding	–0.28 (0.33)	0.4	0.33 ± 0.27 (0.00, 0.59)
	Planktonic feeding	0.61 (0.28)	**0.03**	0.35 ± 0.25 (0.00, 0.60)
Fertilization mode	Annelid	–1.06 (0.32)	**<0.01**	**–1.34 ± 0.37 (−2.21, −0.71)**
	Echinodermata	–3.63 (0.58)	**<0.01**	**–3.52 ± 0.73 (−5.58, −2.26)**
	Mollusca	–2.85 (0.28)	**<0.01**	**–0.71 ± 0.34 (−1.19, −0.46)**
Adult mass	Annelid	–0.23 (0.05)	**<0.01**	**–0.20 ± 0.05 (−0.29, −0.04)**
	Echinodermata	–0.61 (0.10)	**<0.01**	**–0.54 ± 0.10 (−0.72, −0.12)**
	Mollusca	–0.23 (0.03)	**<0.01**	–0.03 ± 0.02 (−0.06, 0.00)
Latitude	Annelid	–0.03 (0.01)	**0.02**	**–0.03 ± 0.01 (−0.06, −0.01)**
	Echinodermata	0.01 (0.01)	0.57	0.01 ± 0.01 (−0.01, 0.04)
	Mollusca	–0.03 (0.01)	**<0.01**	–0.01 ± 0.00 (−0.01, 0.00)

### DEVELOPMENTAL MODE AND HERMAPHRODITISM

Hermaphroditism covaried with developmental mode, but patterns in molluscs were different to those in the other two phyla, and depended on the inclusion of phylogeny (Tables 2 and 3). In annelids and echinoderms, hermaphrodites were rare in species with feeding larvae and relatively common in species with aplanktonic development (Tables 1 and 2; Fig. S2). In contrast, molluscs with feeding larvae were most likely to be hermaphroditic, but accounting for phylogeny weakened this pattern (Table 3).

### BODY SIZE, LATITUDE, AND HERMAPHRODITISM

**Figure 1:**
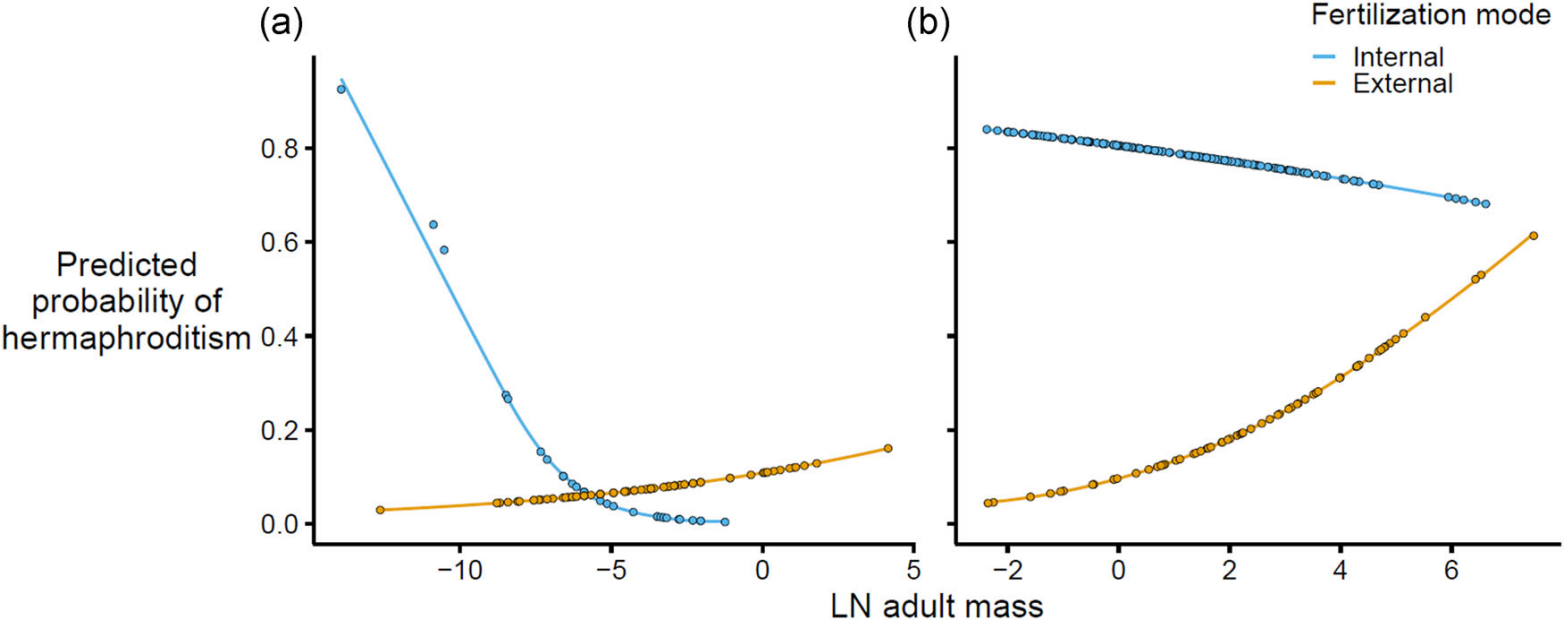
Relationship between body size and the prevalence of hermaphroditism according to fertilization mode in species with feeding larvae for (a) annelids and (b) molluscs. Gradients are lines of best fit from standard logistic regressions, and points represent fitted values for each species to show the distribution of data across the size range.

Generally, hermaphrodites were smaller than gonochores (Tables 2 and 3). The key exceptions were both annelids and molluscs with feeding larvae, where hermaphrodites were smaller in internal fertilizers and larger in external fertilizers (annelids: *χ* ^2^_fertilization mode × adult mass_ = 8.18; molluscs: *χ* ^2^_fertilization mode × adult mass_ = 6.55; df = 1 and *P* < 0.01 for both; Fig. 1).

**Figure 2:**
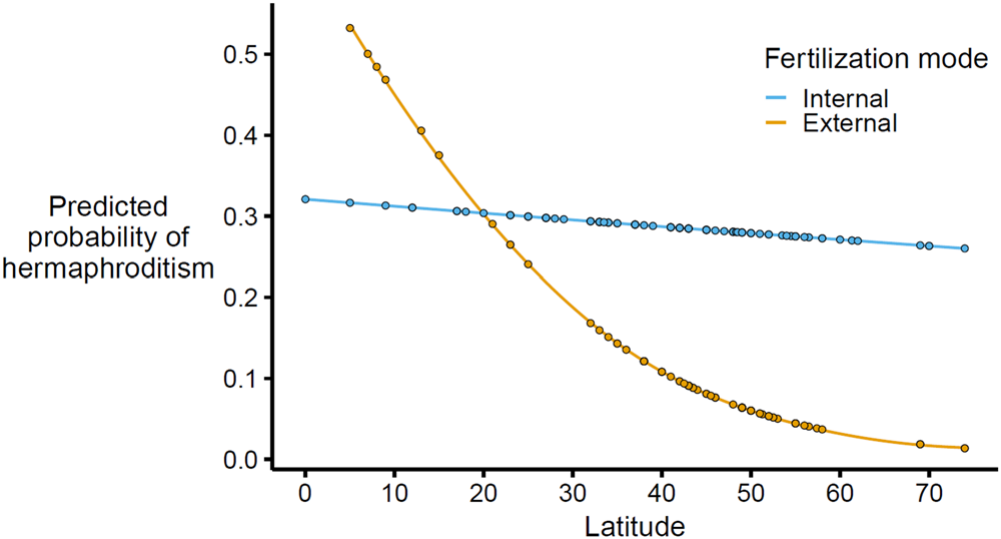
Latitudinal gradients in hermaphroditism, fitted by fertilization mode in annelids with nonfeeding larvae. The gradients are lines of best fit from standard logistic regressions, and points represent fitted values for each species to show the distribution of data across the latitudinal range.

In annelids, hermaphrodites were rarer at the poles than at the tropics (Table 2). Exploring the annelids in more detail revealed some exceptions to the overall pattern—at lower latitudes, hermaphrodites were more common in external fertilizers than in internal fertilizers, but at higher latitudes the opposite was true (*χ* ^2^_fertilization mode × latitude_ = 4.30, df = 1, *P* = 0.04; Fig. 2). Molluscs showed a similar pattern to that of annelids (hermaphroditism was rarer at the poles), but accounting for phylogeny weakened this relationship (Table 3). In echinoderms, latitude did not covary with hermaphroditism at all (Table 3).

### INFLUENCE OF PHYLOGENETIC RESOLUTION

In total, 75 of our 76 parameter estimates generated from models with randomly resolved polytomies were qualitatively the same as our original estimates, suggesting that most of the patterns we observed do not depend on how the polytomies were resolved (Tables S2 and S3). Only one estimate—the effect of latitude in molluscs with feeding larvae—depended on how the polytomies were resolved, so we are hesitant to make strong inferences in this case. Whether latitudinal gradients in hermaphroditism in molluscs differ from those of other animals is unclear, and better‐resolved phylogenies in molluscs are needed to understand this effect.

### PATTERNS IN SIMULTANEOUS VERSUS SEQUENTIAL HERMAPHRODITES

Across all phyla, our main finding that internal fertilizers are more likely to be hermaphrodites than external fertilizers is consistent for sequential and simultaneous hermaphrodites (Tables S4 and S5). For all other effects, in echinoderms, patterns are consistent across hermaphroditism types, and in annelids, patterns are weaker in sequential hermaphrodites—in both cases, this is because sequential hermaphrodites make up such a small fraction of the dataset (Table S4). In molluscs, patterns in developmental mode differed between hermaphroditism types—sequential hermaphrodites behave more like annelids and echinoderms (species with aplanktonic development are more likely to be hermaphroditic than species with nonfeeding and feeding larvae), whereas simultaneous hermaphrodites show the opposite pattern (Table S5). There was no relationship between adult size and hermaphroditism in sequentially‐ hermaphroditic molluscs (Table S4).

### SUMMARY

In terms of both consistency and strength of relationship, fertilization mode was the principal factor that varied with hermaphroditism. Developmental mode was also important, but the patterns varied among phyla. Hermaphrodites were generally smaller than gonochores, but hermaphroditism depends on both adult size and fertilization mode in annelids and molluscs with feeding larvae. Hermaphroditism was less common at the poles in annelids and molluscs, but there was no covariance between latitude and hermaphroditism in echinoderms. Our results were largely consistent whether we accounted for phylogenetic relationships among species, and all but one of the patterns we observed were robust to how we resolved polytomies. Most of our results were qualitatively identical for simultaneous and sequential hermaphrodites, aside from patterns for developmental mode and adult size in molluscs.

## Discussion

Theory developed to understand hermaphroditism in plants successfully predicts many patterns of hermaphroditism in animals, implying analogous selective forces drive the evolution of hermaphroditism in both kingdoms. Putting our findings with those for plants, hermaphroditism is generally associated with limited offspring dispersal (wind dispersal/aplanktonic development), more efficient pollination/fertilization modes (animal pollination/internal fertilization), and smaller adult size (likely because sperm limitation at smaller sizes favors hermaphroditism when small [Henshaw et al. 2014]). However, biogeographical patterns in animal hermaphroditism contradict those of plants—hermaphroditism increases with latitude in plants but decreases with latitude in animals. Together, our results suggest that a comprehensive and general theory of the evolutionary drivers of hermaphroditism should be possible, although some patterns are easier to reconcile than others. Below, we explore these important exceptions as well as the implications of the patterns we observe.

### IMPLICATIONS FOR INBREEDING IN ANIMALS

Our results allow us to make a number of predictions about how hermaphroditism should covary with inbreeding, some of which are already supported. Hermaphrodites are typically more inbred than gonochores (Olsen et al. 2020), so it is reasonable to expect groups that are more likely to be hermaphroditic should also be more inbred. For example, we would predict that internal fertilizers and species with aplanktonic development should be more inbred than species with external fertilization and/or planktonic larvae. In a comprehensive meta‐analysis of over 148 species of marine invertebrates across 12 phyla, Olsen et al. (2020) confirmed our predictions: species with internal fertilization and aplanktonic offspring development are more inbred. However, because hermaphroditism covaries with fertilization mode and developmental mode, our interpretation of this relationship becomes more complicated—inbreeding may covary directly with hermaphroditism, or indirectly with traits associated with hermaphroditism (internal fertilization and aplanktonic development). An important next step is to compare levels of inbreeding between hermaphrodites and gonochores within fertilization and developmental modes. Such an analysis would help tease apart the relative effects of hermaphroditism, fertilization mode, and developmental mode on inbreeding.

We would also predict that inbreeding covaries with size and biogeography in animals but as far as we are aware, this remains untested in marine animals. The only test of biogeographical patterns in inbreeding in animals thus far focused exclusively on terrestrial and freshwater species and found no such patterns (De Kort et al. 2021), but marine organisms should now be explored.

### DRIVERS OF HERMAPHRODITISM IN FISHES

Most studies on hermaphroditism in fishes have focused on the social drivers of sequential hermaphroditism (Warner 1975), but the life‐history drivers identified here are also likely to be important. Although simultaneous hermaphroditism is generally rare in fishes, in theory, it should be driven by competition for resources or among gametes in similar ways as in plants and marine invertebrates (Charnov 1982; Avise and Mank 2009). If we accept that offspring dispersal potential drives hermaphroditism, we would expect fishes that lay demersal eggs with limited dispersal (Kasimatis and Riginos 2016) to be more likely to be hermaphroditic than fishes that broadcast planktonic eggs. In terms of size, we would expect hermaphroditism to be more common in live‐bearing fishes, which are generally smaller and thus may experience greater sperm limitation than their egg‐laying counterparts (Wourms and Lombardi 1992; but see Goodwin et al. 2002). Whether simultaneous hermaphroditism covaries with life history in fishes is unclear, and more systematic attempts to map the drivers of simultaneous hermaphroditism in fishes are needed (Petersen 2006; Pla 2019).

### DRIVERS OF HERMAPHRODITISM IN OTHER TAXA

We found consistent drivers of hermaphroditism across three phyla but there are other groups of hermaphroditic clades that could improve our understanding of the life‐history and ecological correlates of hermaphroditism in animals. For example, some groups of planktonic invertebrates including chaetognaths, ctenophores, and larvaceans are exclusively hermaphroditic (Krumbach 1927; Pianka 1974; Diebel and Lowen 2012), and may allow us to determine if patterns in hermaphroditism differ between benthic and planktonic animals. Furthermore, corals and bryozoans are largely hermaphroditic (Reed 1987; Baird et al. 2009), and because they are colonial organisms, they may offer closer analogues to plants in terms of the selective pressures that drive the evolution of hermaphroditism. For example, if larger plants are more likely to self‐fertilize, and thus have separate sexes to avoid selfing (Maynard Smith 1978), and colonial animals are more likely to self‐fertilize than solitary animals, then we might expect the association between small size and hermaphroditism to be driven by inbreeding avoidance in colonial animals (Jarne and Auld 2006) and sperm limitation in solitary species. Whether our findings apply to patterns in animal hermaphroditism more generally is unclear, and tests in other taxa are needed.

### IMPORTANCE OF MULTIPLE PREDICTORS

Our study highlights the importance of testing multiple predictors simultaneously when evaluating the drivers of hermaphroditism. For example, we found that hermaphroditism (co)varies with fertilization mode and latitude/mass, and that these patterns differ among developmental modes. By considering multiple predictors, we were able to build upon previous findings for how hermaphroditism is predicted to vary with fertilization mode (Iyer and Roughgarden 2008) and offspring developmental mode (Heller 1993) independently in marine invertebrates.

### DRIVERS OF HERMAPHRODITISM IN MOLLUSCS

Many of the patterns in hermaphroditism we observe in molluscs differ from those of the other two phyla and from our theoretical expectations for how hermaphroditism should covary with life history and ecology, but we suspect that our sample of molluscs was insufficiently representative. A large portion (40%) of the molluscs in our dataset are sea slugs, with relatively few (19%) species of bivalves, so we acknowledge that our findings may not reflect patterns of hermaphroditism in molluscs more generally. Overall, the drivers of hermaphroditism in molluscs are less robust and less well‐resolved than in the annelids and echinoderms in our dataset. Therefore, we are inclined to treat our findings with regard to molluscs with caution until additional data on the incidence of hermaphroditism in this group can be assembled for a more representative sample.

### BIOGEOGRAPHICAL PATTERNS IN HERMAPHRODITISM

Contrary to biogeographical patterns in plants, hermaphroditism tends to decline with latitude in the marine invertebrates we studied. Beyond our compilation, we find mixed support for latitudinal gradients in hermaphroditism—similar patterns to ours occur in corals and fishes (Baird et al. 2009; Pla et al. 2021), but some crustaceans show similar patterns to plants (Longhurst 1955; Mathers et al. 2013). Because life history also (co)varies strongly with latitude (Marshall et al. 2012; Monro and Marshall 2015), hermaphroditism may be driven more by life history than biogeography in the marine invertebrates we considered. For example, in molluscs, latitudinal patterns in hermaphroditism disappear when we account for fertilization mode. Although mate limitation appears to favor hermaphroditism at higher latitudes in plants, whether similar forces drive latitudinal gradients in animal hermaphroditism is unclear. It will be interesting to see whether our predictions for biogeographical patterns in hermaphroditism are supported in other animal groups, or whether they show similar patterns to plants.

### ROLE OF SEQUENTIAL VERSUS SIMULTANEOUS HERMAPHRODITISM

Our results allow us to compare differences in patterns of sequential and simultaneous hermaphroditism in molluscs to those of other animals. For example, Pla et al. (2021) found that sequentially hermaphroditic fishes are more common at the tropics and more associated with reef habitats than simultaneous hermaphrodites. We find a different latitudinal pattern in molluscs—hermaphrodites are more common at the tropics than at the poles, regardless of hermaphroditism type, but whether sequential hermaphrodites are more commonly associated with reef habitats than simultaneous hermaphrodites is unknown. The association between small size and hermaphroditism in simultaneously hermaphroditic molluscs is congruent with theory developed in plants (de Jong and Klinkhamer 2005). However, there is no relationship between size and hermaphroditism in sequentially hermaphroditic molluscs, suggesting that the theory developed to explain the evolution of sequential hermaphroditism in fishes (Warner et al. 1975; Munday et al. 2006) may not apply to molluscs. Overall, whether the life‐history and ecological drivers of hermaphroditism in animals differ between simultaneous and sequential hermaphrodites is unclear, and tests in other taxa are needed.

## Conclusions

We applied a predictive framework developed in plants to test whether hermaphroditism is driven by similar forces across the tree of life. Our findings support many of the longstanding hypotheses that hermaphroditism is driven by fertilization/pollination mode, offspring dispersal potential, adult size, and biogeography (Ghiselin 1969; Heath 1979; Strathmann et al. 1984; Rouse and Fitzhugh 1994; Hart et al. 1997), and that local competition (for either resources or among gametes) may drive the evolution of hermaphroditism in both plants and animals. Correlational approaches cannot unequivocally determine evolutionary chains of causality—although the patterns we observed are consistent with theory, the ultimate drivers of hermaphroditism remain unclear. That we find consistent covariances between our traits of interest despite potential clade‐specific idiosyncrasies implies that the traits coevolve or are strongly, consistently correlated with additional, as yet unidentified, drivers of hermaphroditism. Overall, the broadness of our findings provides more support for Darwin's (1876) supposition from over a century ago: hermaphroditism evolves in response to organisms’ life history and ecology—not the other way around.

## AUTHOR CONTRIBUTIONS

DJM conceived the study. GCJ compiled and standardized the dataset. GCJ analyzed the data. GCJ wrote the first draft of the manuscript. All authors contributed substantially to revisions.

## CONFLICT OF INTEREST

The authors declare no conflict of interest.

## DATA ARCHIVING

All raw data and code for analyses are deposited in the Dryad Digital Repository: https://doi.org/10.5061/dryad.76hdr7t0v.

Associate Editor: Erik E. Sotka

Handling Editor: Andrew G. McAdam

## Supporting information

Additional supporting information may be found online in the Dryad Digital Repository for this article: https://doi.org/10.5061/dryad.76hdr7t0v.
