# Correction to: Macroevolutionary patterns in marine hermaphroditism

**DOI:** 10.1093/evolut/qpad127

**Published:** 2023-08-01

This is a correction to: George C. Jarvis and others, Macroevolutionary patterns in marine hermaphroditism, *Evolution*, Volume 76, Issue 12, 1 December 2022, Pages 3014–3025, https://doi.org/10.1111/evo.14639

In the originally published version of this manuscript, the authors’ in-text reporting of results underestimated the percent differences in the likelihood of hermaphroditism between internal and external fertilizers within each phylum. While the error does not affect the statistical results or the overall findings (in all phyla, internal fertilizers are more likely to be hermaphroditic than external fertilizers), amendments have been made to the values of the percentages listed in the **Results** section, first paragraph, third sentence which should read: “Internal fertilizers were 35, 4.4, and 2.4 times more likely to be hermaphroditic than external fertilizers for echinoderms, molluscs, and annelids, respectively (Table 3, Fig. S1)” instead of: “Internal fertilizers were 15%, 30%, and 60% more likely to be hermaphroditic than external fertilizers for annelids, echinoderms, and molluscs, respectively (Table 3).”

This error has been corrected within the article.

