# Supplementary material for: Macroevolutionary patterns in marine hermaphroditism
Source: Evolution. 2022 Oct 13;76(12):3014–25. doi: 10.1111/evo.14639 (PMC10091813; doi:10.1111/evo.14639)
Supplement: Supplementary file 1 — Additional supporting information may be found online in the Dryad Digital Repository for this article: https://doi.org/10.5061/dryad.76hdr7t0v. [file EVO-76-3014-s002.pdf]

Supplemental material

Supplementary table 1. Combinations of life-history traits and latitude tested in our analyses. Shaded cells represent combinations that were not tested because they were absent or poorly represented in our dataset (see Methods for details).

Phylum	Trait(s)	Developmental mode		
		Aplanktonic	Planktonic non-feeding	Planktonic feeding
Annelida	Fertilization mode (F)			
	Adult mass (M)			
	F x M			
Echinodermata	F			
	M			
	F x M			
Mollusca	F			
	M			
	F x M			
Annelida	F			
	Latitude (L)			
	F x L			
Echinodermata	F			
	L			
	F x L			
Mollusca	F			
	L			
	F x L			

Supplementary table 2. Tests of phylogenetic uncertainty on the covariation between hermaphroditism, and single effects of fertilization mode, developmental mode, adult mass, and latitude. Values represent estimates from our models, with the range of estimates generated from 1000 bootstrapped models where polytomies in the phylogenetic trees were randomly resolved shown in parenthesis. The shaded cells represent the cases where our estimates fall outside of the range of bootstrapped estimates, and our qualitative result depends on how the polytomies were resolved. Parameter estimates for developmental mode and fertilization mode are in reference to species with aplanktonic development and internal fertilization, respectively.

Parameter	Phylum		
	Annelida	Echinodermata	Mollusca
Developmental mode			
Planktonic non-feeding	0.1300 (-0.4565, 0.3472)	-2.9000 (-3.0211, -0.6282)	0.3500 (-0.2267, 0.5361)
Planktonic feeding	-0.7100 (-1.4007, -0.4888)	-5.1600 (-5.2669, -1.0019)	0.3300 (-0.0973, 0.5626)
Fertilization mode	-1.3600 (-1.5858, -0.9064)	-3.5400 (-3.8065, -3.3232)	-0.7100 (-1.3238, 0.1611)
Adult mass	-0.2000 (-0.2291, 0.0087)	-0.5400 (-0.6430, -0.4211)	-0.0300 (-0.0386, 0.0512)
Latitude	-0.0300 (-0.0612, -0.0131)	0.0100 (0.0080, 0.0298)	-0.0050 (-0.0080, 0.0074)
α	38.6200 (51.9405, 54.5905)	54.2900 (50.3577, 54.5748)	3.0600 (4.1960, 5.9684)
a (-log[α])	-3.6538 (-3.9501, -3.9999)	-3.9943 (-3.9192, -3.9996)	-1.1184 (-1.4341, -1.7865)

Supplementary table 3. Tests of phylogenetic uncertainty on the covariation between hermaphroditism, fertilization mode, developmental mode, adult mass, and latitude. The shaded cell represents the single case where our estimates fall outside of the range of bootstrapped estimates, and our qualitative result depends on how the polytomies were resolved. Coefficients for fertilization mode ('Fert') and its combination with other parameters is in reference to internal fertilizers. See Supplemental table 2 legend for further details.

Phylum	Parameter(s)	Model type	Developmental mode		
			Aplanktonic	Planktonic non-feeding	Planktonic feeding
Annelida	Mass	Full	0.0000 (-0.2291, 0.0087)	-0.1800 (-0.3249, -0.0884)	-0.2400 (-0.6779, -0.5074)
	Fert	Full	--	-0.0800 (-1.1202, 0.7575)	1.6100 (3.8159, 4.7827)
	Fert x Mass	Full	--	0.0800 (-0.0508, 0.1672)	0.3000 (0.6596, 0.9382)
	Fert	Reduced	--	-0.7800 (-1.0207, 0.4114)	--
	Mass	Reduced	--	-0.1000 (-0.2295, -0.0831)	--
Echinodermata	Mass	Full	-0.3200 (-0.3876, -0.2575)	-0.1900 (-0.4252, -0.0114)	--
	Fert	Full	-1.5000 (-1.7142, -1.2554)	-2.1000 (-2.0838, 0.1329)	--
	Fert x Mass	Full	0.0700 (0.0097, 0.1629)	-0.4500 (-0.5976, 0.1179)	--
	Mass	Reduced	-0.3200 (-0.3806, -0.2718)	-0.5400 (-0.6999, -0.0513)	--
	Fert	Reduced	-1.5100 (-1.9193, -1.3336)	-1.3600 (-2.4246, -0.1633)	--
Mollusca	Mass	Full	0.0500 (0.0396, 0.0879)	0.0000 (-0.0975, 0.0022)	-0.0500 (-0.0819, -0.0190)
	Fert	Full	--	0.0200 (-0.7236, -0.0064)	-2.7100 (-3.5829, -0.2335)
	Fert x Mass	Full	--	-0.0200 (0.0004, 0.2152)	0.3100 (-0.1101, 0.4862)
	Fert	Reduced	--	0.0100 (-3.7255, -0.0280)	--
	Mass	Reduced	--	0.0000 (-0.0180, 0.2750)	--
Annelida	Latitude	Full	0.0500 (0.0732, 0.0740)	-0.0200 (-0.0245, 0.0068)	-0.0400 (-0.0456, -0.0368)

	Fert	Full	--	1.2000 (1.0415, 1.2048)	-1.2680 (-1.2688, -0.7008)
	Fert x Latitude	Full	--	-0.0600 (-0.0663, -0.0429)	0.0300 (0.0083, 0.0383)
	Fert	Reduced	--	--	-0.7800 (-0.7928, 0.0480)
	Latitude	Reduced	--	--	-0.0200 (-0.0710, -0.0199)
Echinodermata	Latitude	Full	-0.0460 (-0.0493, -0.0415)	0.0140 (0.0148, 0.0315)	--
	Fert	Full	-2.7690 (-2.7690, -2.7685)	-0.0200 (-0.0701, 1.0371)	--
	Fert x Latitude	Full	0.0310 (0.0304, 0.0388)	-0.0200 (-0.0480, -0.0190)	--
	Latitude	Reduced	-0.0361 (-0.0363, -0.0344)	0.0200 (-0.0131, 0.0293)	--
	Fert	Reduced	-1.9190 (-1.9195, -1.9188)	-2.1300 (-2.1318, -1.5238)	--
Mollusca	Latitude	Full	0.0000 (-0.0251, 0.0047)	-0.0300 (-0.0326, -0.0298)	-0.0200 (-0.0203, -0.0064)
	Fert	Full	--	-8.4087 (-8.4087, -8.4084)	-2.0824 (-2.0831, -2.0814)
	Fert x Latitude	Full	--	0.1210 (0.1216, 0.1260)	0.0200 (0.0127, 0.0420)
	Fert	Reduced	--	0.0100 (-4.2547, 0.0128)	-2.9500 (-4.2547, 0.0128)
	Latitude	Reduced	--	0.0000 (-0.0051, 0.0251)	-0.0300 (-0.0051, 0.0251)

Supplementary table 4. Standard logistic regressions testing the effects of hermaphroditism type (simultaneous vs. sequential) on patterns in reproductive mode in marine invertebrates. Orange cells represent cases where patterns for sequential hermaphrodites differed from simultaneous hermaphrodites. Sample sizes for each hermaphroditism type are shown in parenthesis (sample sizes by phylum: Annelids [simultaneous. n = 42, sequential. n = 13], Echinoderms [sim. n = 16, seq. n = 9], and Molluscs [sim. n = 217, seq. n = 62]). For further details, see Table 2.

Trait	Phylum	Simultaneous and seqential hermaphrodites (n = 359)		Simultaneous hermaphrodites only (n = 275)		Sequential hermaphrodites only (n = 84)	
		χ^2	*P*	χ^2	*P*	χ^2	*P*
Developmental mode	Annelida	8.15	**0.02**	22.93	< **0.05**	3.15	0.21
	Echinodermata	65.35	< **0.01**	49.05	< **0.05**	20.16	< **0.05**
	Mollusca	15.10	< **0.01**	23.85	< **0.05**	19.64	< **0.05**
Fertilization mode	Annelid	12.13	< **0.01**	15.91	< **0.05**	0.03	0.86
	Echinodermata	42.79	< **0.01**	25.81	< **0.05**	19.10	< **0.05**
	Mollusca	138.57	< **0.01**	138.04	< **0.01**	37.37	< **0.01**
Adult mass	Annelid	29.36	< **0.01**	33.46	< **0.01**	1.33	0.25
	Echinodermata	50.84	< **0.01**	36.67	< **0.01**	16.96	< **0.01**
	Mollusca	49.33	< **0.01**	71.99	< **0.01**	0.02	0.9
Latitude	Annelid	5.74	< **0.01**	5.16	**0.02**	0.82	0.37
	Echinodermata	0.32	0.57	0.39	0.53	0.01	0.91
	Mollusca	16.92	< **0.01**	14.78	< **0.01**	8.30	< **0.01**

Supplementary table 5. Estimates for standard (± SE) logistic regressions testing the effects of hermaphroditism type (simultaneous vs. sequential) on patterns in reproductive mode. Orange cells represent cases where patterns depended on hermaphroditism type. For further details, see Table 3 and Supplemental table 4 legends.

Trait	Phylum	Simultaneous and sequential hermaphrodites (n = 359)		Simultaneous hermaphrodites only (n = 275)		Sequential hermaphrodites only (n = 84)	
		Coefficient (± SE)	*P*	*Coefficient (± SE)*	*P*	*Coefficient (± SE)*	*P*
Developmental mode	Annelida						
	Planktonic non-feeding	0.26 (0.52)	0.62	0.38 (0.57)	**0.02**	-0.43 (1.14)	0.71
	Planktonic feeding	-0.74 (0.59)	0.21	-2.13 (0.90)	0.50	0.64 (1.08)	0.55
	Echinodermata						
	Planktonic non-feeding	-2.46 (0.53)	< **0.01**	-3.02 (0.78)	< **0.05**	-1.77 (0.73)	< **0.05**
	Planktonic feeding	-19.78 (1.24 x 10^3)	0.99	-19.42 (1.24 x 10^3)	0.99	-19.57 (2.04 x 10^3)	0.99
	Mollusca						
	Planktonic non-feeding	-0.28 (0.33)	0.4	0.70 (0.40)	0.08	-0.71 (0.34)	0.08
	Planktonic feeding	0.61 (0.28)	**0.03**	1.49 (0.37)	< **0.05**	-2.25 (0.59)	< **0.05**
Fertilization mode	Annelid	-1.06 (0.32)	< **0.01**	-1.41 (0.38)	< **0.05**	-0.10 (0.57)	0.87
	Echinodermata	-3.63 (0.58)	< **0.01**	-3.60 (0.72)	< **0.01**	-3.67 (0.87)	< **0.05**
	Mollusca	-2.85 (0.28)	< **0.01**	-3.18 (0.34)	< **0.01**	-2.10 (0.39)	< **0.01**
Adult mass	Annelid	-0.23 (0.05)	< **0.01**	-0.29 (0.05)	< **0.01**	-0.09 (0.08)	0.25
	Echinodermata	-0.61 (0.10)	< **0.01**	-0.65 (0.13)	< **0.01**	-0.54 (0.14)	< **0.01**
	Mollusca	-0.23 (0.03)	< **0.01**	-0.29 (0.04)	< **0.01**	0.01 (0.06)	0.9
Latitude	Annelid	-0.03 (0.01)	**0.02**	-0.03 (0.01)	**0.02**	-0.02 (0.02)	0.36
	Echinodermata	0.01 (0.01)	0.57	-0.01 (0.01)	0.53	0.00 (0.02)	0.91
	Mollusca	-0.03 (0.01)	< **0.01**	-0.03 (0.01)	< **0.01**	-0.03 (0.01)	< **0.01**

Supplementary table 6. Standard logistic regressions testing the interactive effects of fertilization mode, offspring developmental mode, adult mass (shaded cells), and latitude on hermaphroditism in marine invertebrates. Main effects and their interactions were evaluated using analysis of deviance tests, and models were reduced when interactions were not significant ($P > 0.05$). Dashed cells represent combinations of life-history traits that were not tested because they were rare or absent in our dataset. df = 1 for all. (***P* < 0.05**)

Phylum	Trait	Aplanktonic		Planktonic non-feeding		Planktonic feeding	
		χ^2	*P*-value	χ^2	*P*-value	χ^2	*P*-value
	Fertilization mode (F)	--	--	0.05	0.82	5.16	**0.02**
Annelida	Adult mass (M)	0.40	0.53	14.85	**< 0.01**	8.99	**< 0.01**
	F x M	--	--	0.00	0.99	8.18	**< 0.01**
	F	3.33	0.07	1.56	0.21	--	--
Echinodermata	M	6.55	**0.01**	0.54	0.46	--	--
	F x M	0.03	0.86	0.04	0.85	--	--
	F	--	--	39.53	**< 0.01**	55.69	**< 0.01**
Mollusca	M	0.17	0.68	1.66	0.20	0.94	0.33
	F x M	--	--	3.38	0.07	6.55	**0.01**
	F	--	--	1.20	0.27	0.19	0.66
Annelida	Latitude (L)	2.19	0.14	0.07	0.79	1.03	0.31
	F x L	--	--	4.30	**0.04**	0.03	0.86
	F	1.46	0.23	0.00	0.99	--	--
Echinodermata	L	7.82	**0.01**	0.09	0.76	--	--
	F x L	0.16	0.69	0.17	0.68	--	--
	F	--	--	14.61	**< 0.01**	2.53	0.11
Mollusca	L	2.35	0.13	0.19	0.66	0.63	0.43
	F x L	--	--	3.35	0.07	1.71	0.19

Supplementary table 7. Estimates for standard (± SE) and phylogenetically-controlled (± SE with bootstrapped 95% confidence intervals) logistic regressions testing the drivers of hermaphroditism in marine invertebrates. Model intercepts were excluded for brevity, with estimates of coefficients in reference to species with aplanktonic offspring development and internal fertilization for tests of developmental and fertilization mode, respectively. Values for α and a (= -log[α]) represent parameter estimates ± 95% confidence intervals, testing for phylogenetic signal in the residuals of the phylogenetically-controlled logistic regressions (Ives and Garland 2010). Significant phylogenetic signal in the residuals is indicated by 95% confidence intervals around a that do not overlap < -4 (**bolded** [Ives & Garland 2010]). Note that coefficient estimates are on a logit scale.

Trait	Phylum	Standard regressions		Phylogenetically-controlled regressions		
		Coefficient (± SE)	*P*-value	Coefficient ± SE (95% CI)	α (95% CI)	*a* (95% CI)
Developmental mode	Annelida				14.34 (5.48, 35.46)	**-2.66 (-1.70, -3.57)**
	Planktonic non-feeding	0.26 (0.52)	0.62	0.13 ± 0.42 (-0.52, 0.67)		
	Planktonic feeding	-0.74 (0.59)	0.21	-0.71 ± 0.51 (-1.49, 0.03)		
	Echinodermata				21.88 (0.02, 54.59)	-3.09 (3.91, -4.00)
	Planktonic non-feeding	-2.46 (0.53)	**< 0.01**	**-2.90 ± 0.77 (-4.75, -0.63)**		
	Planktonic feeding	-19.78 (1.24e^3)	0.99	**-5.16 ± 1.48 (-19.21, -0.77)**		
	Mollusca				1.64 (0.32, 3.25)	**-0.49 (1.14, -1.18)**
	Planktonic non-feeding	-0.28 (0.33)	0.4	0.33 ± 0.27 (0.00, 0.59)		
	Planktonic feeding	0.61 (0.28)	**0.03**	0.35 ± 0.25 (0.00, 0.60)		
Fertilization mode	Annelid	-1.06 (0.32)	**< 0.01**	**-1.34 ± 0.37 (-2.21, -0.71)**	7.60 (1.21, 40.45)	**-2.03 (-0.19, -3.70)**
	Echinodermata	-3.63 (0.58)	**< 0.01**	**-3.52 ± 0.73 (-5.58, -2.26)**	20.65 (3.28, 54.56)	-3.03 (-1.19, -4.00)

	Mollusca	-2.85 (0.28)	< **0.01**	**-0.71 ± 0.34 (-1.19, -0.46)**	1.94 (0.92, 4.18)	**-0.66 (0.08, -1.43)**
Adult mass	Annelid	-0.23 (0.05)	< **0.01**	**-0.20 ± 0.05 (-0.29, -0.04)**	45.00 (2.87, 54.58)	-3.81 (-1.05, -4.00)
	Echinodermata	-0.61 (0.10)	< **0.01**	**-0.54 ± 0.10 (-0.72, -0.12)**	53.58 (0.09, 54.59)	-3.98 (2.41, -4.00)
	Mollusca	-0.23 (0.03)	< **0.01**	-0.03 ± 0.02 (-0.06, 0.00)	**2.15 (1.00, 3.91)**	**-0.77 (0.00, -1.36)**
Latitude	Annelid	-0.03 (0.01)	**0.02**	**-0.03 ± 0.01 (-0.06, -0.01)**	11.40 (1.00, 16.84)	**-2.43 (0.00, -2.82)**
	Echinodermata	0.01 (0.01)	0.57	0.01 ± 0.01 (-0.01, 0.04)	33.71 (1.00, 36.27)	**-3.52 (0.00, -3.59)**
	Mollusca	-0.03 (0.01)	< **0.01**	-0.01 ± 0.00 (-0.01, 0.00)	1.00 (1.00, 1.00)	**0.00 (0.00, 0.00)**

Supplementary table 8. Estimates for standard and phylogenetically-controlled logistic regressions testing the interactive effects of fertilization mode, offspring developmental mode, and adult mass on hermaphroditism in marine invertebrates. For further details, see Supplementary table 7 legend.

Phylum	Developmental mode	Standard regressions		Phylogenetically-controlled regressions		
		Coefficient (± SE)	*P*-value	Coefficient ± SE (95% CI)	α (95% CI)	*a* (95% CI)
Annelida	Aplanktonic				16.38 (0.05, 54.55)	-2.80 (3.00, -4.00)
	Adult mass (M)	-0.09 (0.14)	0.55	0.00 ± 0.13 (-0.28, 0.28)		
	Planktonic non-feeding				9.86 (1.01, 26.75)	**-2.29 (-0.01, -3.29)**
	Fertilization mode (F)	-0.22 (0.96)	0.82	-0.12 ± 0.81 (-1.22, 0.89)		
	M	-0.28 (0.08)	**< 0.01**	**-0.18 ± 0.08 (-0.31, -0.06)**		
	F x M	0.00 (0.14)	0.99	0.04 ± 0.12 (-0.15, 0.23)		
	Planktonic feeding				54.57 (0.02, 54.59)	-4.00 (3.91, -4.00)
	F	4.29 (2.39)	0.07	0.76 ± 1.30 (-0.56, 4.92)		
	M	-0.64 (0.29)	**0.02**	-0.23 ± 0.17 (-0.82, 0.00)		
	F x M	0.75 (0.32)	**0.02**	0.30 ± 0.21 (0.00, 0.95)		
Echinodermata	Aplanktonic				12.51 (0.04, 55.94)	-2.53 (3.22, -4.02)
	F	-1.55 (0.91)	0.09	-1.50 ± 0.86 (-3.01, 0.00)		
	M	-0.55 (0.25)	**0.03**	-0.32 ± 0.20 (-0.75, 0.05)		
	F x M	0.08 (0.43)	0.86	0.07 ± 0.38 (-0.66, 0.83)		
	Planktonic non-feeding				53.81 (0.00, 54.53)	-3.99 (> 4.00, -4.00)
	F	-2.14 (1.75)	0.22	-2.09 ± 1.26 (-3.35, 0.50)		

	M	-1.24 (3.19)	0.70	-0.19 ± 0.55 (-1.49, 0.44)		
	F x M	0.56 (3.20)	0.17	-0.45 ± 0.60 (-1.11, 1.13)		
Mollusca	Aplanktonic				1.04 (0.00, 4.33)	**-0.04 (> 4.00, -1.47)**
	M	0.05 (0.13)	0.68	0.00 ± 0.04 (-0.08, 0.06)		
	Planktonic non-feeding				0.89 (0.00, 12.08)	**0.12 (> 4.00, -2.49)**
	F	-4.10 (0.84)	**< 0.01**	0.02 ± 0.21 (-3.42, 0.32)		
	M	-0.18 (0.15)	0.22	0.00 ± 0.02 (-0.73, 0.34)		
	F x M	0.45 (0.26)	0.08	-0.02 ± 0.05 (-0.42, 0.81)		
	Planktonic feeding				0.93 (0.47, 3.32)	**0.07 (0.76, -1.20)**
	F	-3.65 (0.25)	**< 0.01**	**-3.10 ± 0.97 (-3.99, -1.89)**		
	M	-0.10 (0.10)	0.33	-0.10 ± 0.08 (-0.24, 0.08)		
	F x M	0.46 (0.18)	**0.01**	**0.39 ± 0.20 (0.04, 0.67)**		

Supplementary table 9. Estimates for standard and phylogenetically-controlled logistic regressions testing the interactive effects of fertilization mode, offspring developmental mode, and latitude on hermaphroditism in marine invertebrates. For further details, see Supplementary table 7 legend.

Phylum	Developmental mode	Standard regressions		Phylogenetically-controlled regressions		
		Coefficient (± SE)	*P*-value	Coefficient ± SE (95% CI)	α (95% CI)	*a* (95% CI)
Annelida	Aplanktonic				**1.00 (1.00, 9.74)**	**0.00 (0.00, -2.28)**
	Latitude (L)	0.07 (0.05)	0.18	**0.07 ± 0.04 (0.03, 0.12)**		
	Planktonic non-feeding				1.00 (0.00, 1.00)	**0.00 (> 4.00, 0.00)**
	Fertilization mode (F)	1.20 (1.10)	0.27	1.20 ± 0.94 (0.00, 1.21)		
	L	0.00 (0.02)	0.79	-0.01 ± 0.01 (-0.02, 0.01)		
	F x L	-0.06 (0.03)	**0.04**	-0.06 ± 0.02 (-0.08, 0.00)		
	Planktonic feeding				1.00 (0.00, 1.00)	**0.00 (> 4.00, 0.00)**
	F	-1.27 (2.95)	0.67	-1.27 ± 1.56 (-1.27, 0.00)		
	L	-0.06 (0.06)	0.37	-0.04 ± 0.03 (-0.05, 0.00)		
	F x L	0.01 (0.07)	0.87	0.03 ± 0.03 (0.00, 0.04)		
Echinodermata	Aplanktonic				1.03 (0.00, 1.03)	**-0.03 (> 4.00, -0.03)**
	F	-2.77 (2.33)	0.23	-2.77 ± 1.65 (-2.77, 0.00)		
	L	-0.07 (0.03)	**0.02**	-0.05 ± 0.02 (-0.06, 0.00)		
	F x L	0.02 (0.05)	0.69	0.03 ± 0.03 (0.00, 0.05)		
	Planktonic non-feeding				1.00 (0.00, 1.00)	**0.00 (> 4.00, 0.00)**
	F	-0.07 (5.36)	0.23	-0.07 ± 5.18 (-0.07, 0.00)		

	L	0.03 (0.11)	0.77	0.01 ± 0.11 (0.00, 0.03)		
	F x L	-0.05 (0.12)	0.68	-0.02 ± 0.12 (-0.03, 0.00)		
Mollusca	Aplanktonic				1.04 (0.02, 1.07)	**-0.04 (3.91, -0.07)**
	L	-0.02 (0.02)	0.13	0.00 ± 0.00 (-0.01, 0.01)		
	Planktonic non-feeding				1.00 (0.00, 1.00)	**0.00 (> 4.00, 0.00)**
	F	-8.41 (2.66)	< **0.01**	-8.41 ± 2.14 (-8.41, 0.00)		
	L	-0.01 (0.03)	0.66	-0.03 ± 0.02 (-0.05, 0.00)		
	F x L	0.09 (0.05)	0.08	0.12 ± 0.04 (0.00, 0.14)		
	Planktonic feeding				1.04 (0.00, 1.04)	**-0.04 (> 4.00, -0.04)**
	F	-1.45 (0.92)	0.12	-1.44± 0.73 (-1.44, 0.00)		
	L	0.01 (0.02)	0.43	-0.02 ± 0.01 (-0.03, 0.01)		
	F x L	-0.03 (0.02)	0.19	0.01 ± 0.02 (-0.02, 0.03)		

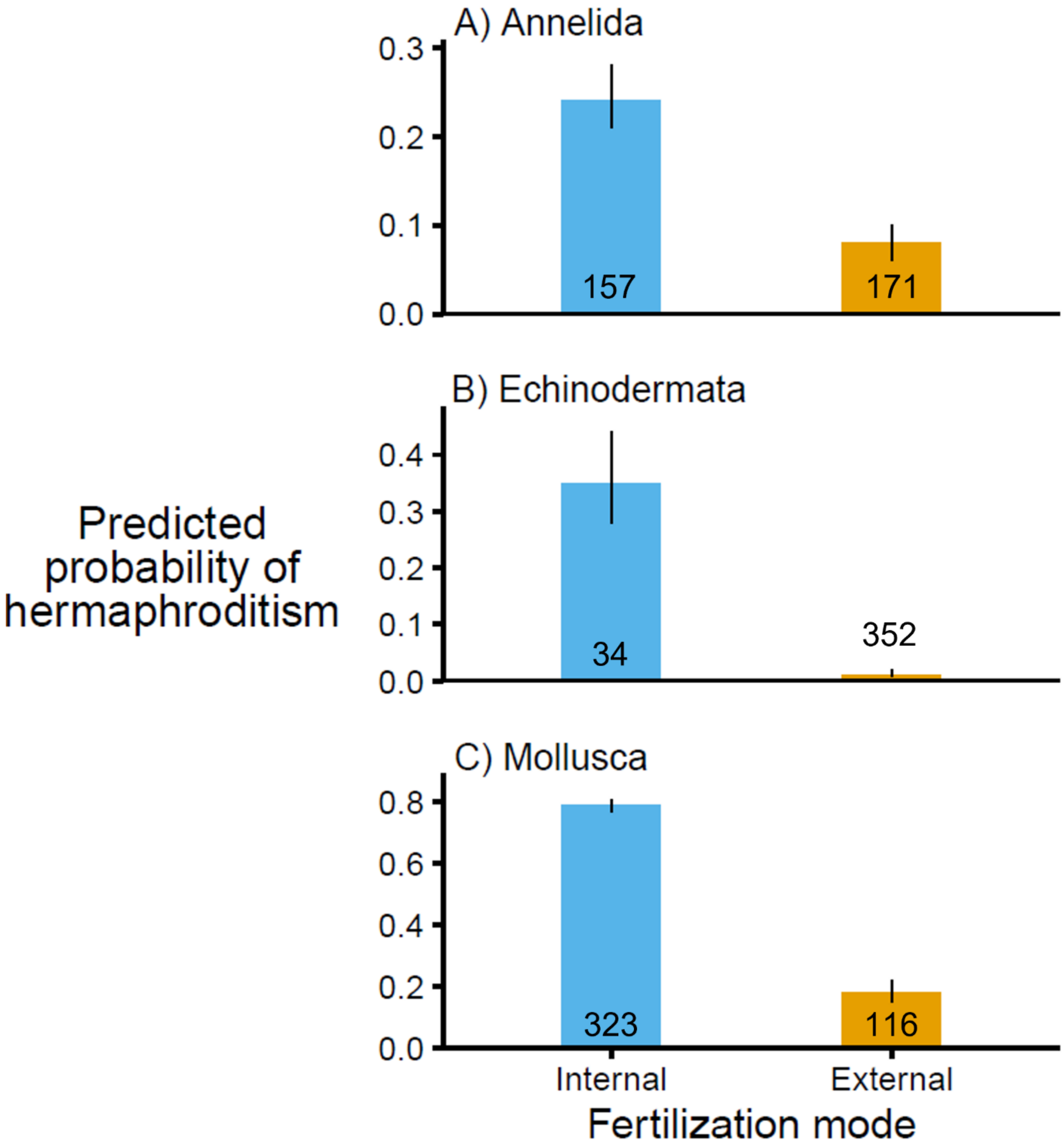

Supplementary figure 1. Prevalence of hermaphroditism according to fertilization mode for three phyla. Bars represent average (± SE) predicted probabilities of hermaphroditism from standard regressions, and numbers represent the number of species for each mode. Note that scales differ among panels.

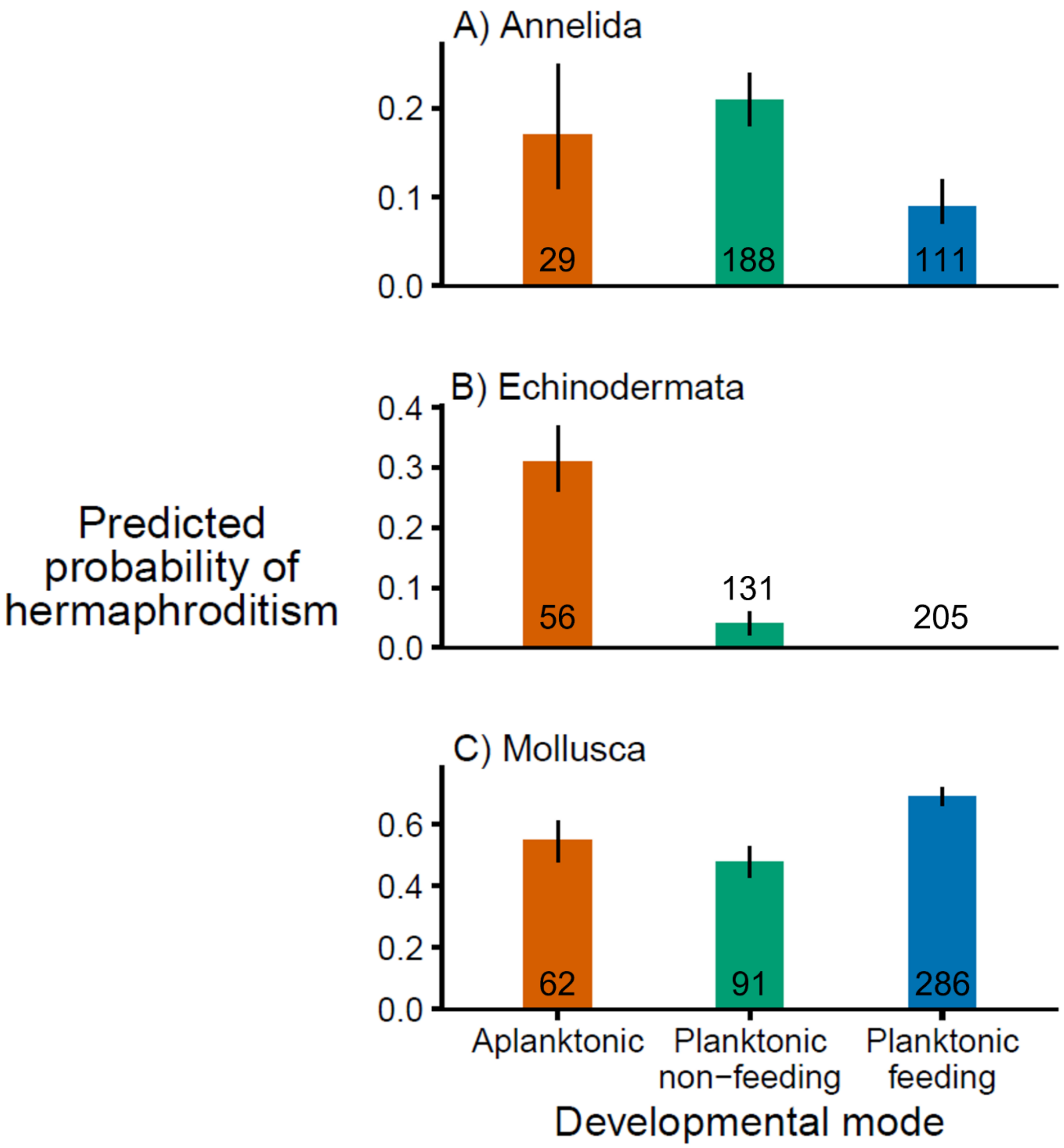

Supplementary figure 2. Prevalence of hermaphroditism accordinig to developmental mode among three phyla. Bars represent average (± SE) predicted probabilities of hermaphroditism from standard regressions, and numbers represent the number of species for each mode. Note that scales differ among panels.

Literature cited

Ives, A. R., and T. Garland. 2010. Phylogenetic logistic regression for binary dependent variables. Syst. Biol. 59:9–26.
