# Supplementary material for: Macroevolutionary patterns in marine hermaphroditism
Source: Evolution. 2022 Oct 13;76(12):3014–25. doi: 10.1111/evo.14639 (PMC10091813; doi:10.1111/evo.14639)
Supplement: Supplementary file 3 [file EVO-76-3014-s001.zip › raw_data_references_trees/references_raw_data.pdf]

References for raw data

Abele, D., C. Tesch, P. Wencke, and H. O. Pörtner. 2001. How does oxidative stress relate to thermal tolerance in the Antarctic bivalve *Yoldia eightsi*? Antarct. Sci. 13:111–118.

Achituv, Y., and R. Devavault. 1972. Nouvelles recherches sur l'hermaphrodisme de *Fromia ghardaqana* Mrtsn (Echinoderme, Astéride). Cah. Biol. Mar. 13:433–442.

Adiyodi, K. G., and R. G. Adiyodi. 1989. Reproduction Biology of Invertebrates - Volume IV. Fertilization, Development, and Parental Care (Part A). John Wiley & Sons Ltd., New York, USA.

Adiyodi, K. G., and R. G. Adiyodi. 1990. Reproduction Biology of Invertebrates - Volume IV. Fertilization, Development, and Parental Care (Part B). John Wiley & Sons Ltd., New York, USA.

Agatsuma, Y. 2013. *Hemicentrotus pulcherrimus*, *Pseudocentrotus depressus*, and *Heliocidaris crassispina*. Pp. 461–473 *in* J. M. Lawrence, ed. Sea Urchins: Biology and Ecology. Elsevier.

Aguiar, T. M., and C. S. Gomes Santos. 2018. Reproductive biology of *Alitta succinea* (Annelida: Nereididae) in a Brazilian tropical lagoon. Invertebr. Biol. 137:17–28.

Ahn, I. Y., and J. H. Shim. 1998. Summer metabolism of the Antarctic clam, *Laternula elliptica* (King and Broderip) in Maxwell Bay, King George Island and its implications. J. Exp. Mar. Bio. Ecol. 224:253–264.

Åkesson, B. 1976. Morphology and life cycle of *Ophryotrocha diadema*, a new polychaete species from California. Ophelia 15:23–35.

Åkesson, B. 1974. Morphology and life history of *Ophryotrocha maculata* sp. n. (Polychaeta, Dorvilleidae). Zool. Scr. 2:141–144.

Åkesson, B. 1973. Reproduction and larval morphology of five *Ophryotrocha* species (Polychaeta, Dorvilleidae). Zool. Scr. 2:145–155.

Åkesson, B. 1967. The embryology of the polychaete *Eunice kobiensis*. Acta Zool. 48:141–192.

Åkesson, B. 1962. The embryology of *Tomopteris helgolandica* (Polychaeta). Acta Zool. 43:135–199.

Aktipis, S. W., E. Boehm, and G. Giribet. 2011. Another step towards understanding the slit-limpets (Fissurellidae, Fissurelloidea, Vetigastropoda, Gastropoda): A combined five-gene molecular phylogeny. Zool. Scr. 40:238–259.

Allen, J. D., and R. D. Podolsky. 2007. Uncommon diversity in developmental mode and larval form in the genus *Macrophiothrix* (Echinodermata: Ophiuroidea). Mar. Biol. 151:85–97.

Allen, M. J. 1959. Embryological development of the polychaetous annelid, *Diopatra cuprea* (Bosc). Biol. Bull. 116:339–361.

Allen, M. J. 1964. Embryological development of the syllid, *Autolytus fasciatus* (Bosc) (Class Polychaeta). Biol. Bull. 127:187–205.

Allen, M. J. 1957. The breeding of polychaetous annelids near Parguera, Puerto Rico. Biol. Bull. 113:49–57.

Allen, R. M., P. J. Krug, and D. J. Marshall. 2009. Larval size in *Elysia stylifera* is determined by extra-embryonic provisioning but not egg size. Mar. Ecol. Prog. Ser. 389:127–137.

Allen, R. M., and D. J. Marshall. 2010. The larval legacy: Cascading effects of recruit phenotype on post-recruitment interactions. Oikos 119:1977–1983.

Alvarado, J. J., and F. A. Solís-Marín. 2014. Echinoderm research and diversity in Latin America.

Amati, B., and I. Nofroni. 2015. The recent Rissoidae of the Mediterranean Sea. Notes on the

genus *Onoba* s.s. H. Adams et A. Adams, 1852 (Gastropoda Prosobranchia). Biodivers. J. 6:467–480.
Amemiya, S., and R. B. Emlet. 1992. The development and larval form of an echinothurioid echinoid, *Asthenosoma ijimai*, revisited. Biol. Bull. 182:15–30.
Andrews, J. C., and D. T. Anderson. 1962. The development of the polychaete *Galeolaria caespitosa* Lamarck (fam. Serpulidae). Proc. Linn. Soc. New South Wales 87:185–188.
Angeloni, L., J. W. Bradbury, and R. S. Burton. 2003. Multiple mating, paternity, and body size in a simultaneous hermaphrodite, *Aplysia californica*. Behav. Ecol. 14:554–560.
Ansell, A. D. 1973. Oxygen consumption by the bivalve *Donax vittatus* (da Costa). J. Exp. Mar. Bio. Ecol. 11:311–328.
Anthes, N., and N. K. Michiels. 2007. Reproductive morphology, mating behavior, and spawning ecology of cephalaspid sea slugs (Aglajidae and Gastropteridae). Invertebr. Biol. 126:335–365.
Arellano, S. M., A. L. Van Gaest, S. B. Johnson, R. C. Vrijenhoek, and C. M. Youn. 2014. Larvae from deep-sea methane seeps disperse in surface waters. Proc. R. Soc. B Biol. Sci. 281.
Arkronrat, W., C. Printrakoon, and V. Oniam. 2016. Occurrence of ragged sea hare (*Bursatella leachii* de Blainville, 1817) in marine shrimp ponds, Prachuap Khiri Khan province, Thailand. Pp. 835–840 *in* 1, ed. Agricultural Innovation for Global Value Chain, Proceedings of 54th Kasetsart University Annual Conference. Kasetsart University, Thailand.
Arnaud, P. M. 1974. Contribution a la bionomie marine benthique des regions antarctiques et subantarctiques. Tethys 6:465–656.
Arntz, W. E., T. Brey, D. Gerdes, M. Gorny, J. Gutt, S. Hain, and M. Klages. 1992. Patterns of life history and population dynamics of benthic invertebrates under the high Antarctic conditions of the Weddel Sea. Pp. 221–230 *in* G. Colombo, I. Ferrari, V. U. Ceccherelli, and R. Rossi, eds. Marine eutrophication and population dynamics; Proc 25th European Marine Biology Symposium. Olsen & Olsen, Fredensborg, Denmark.
Arranz, K., U. Labarta, M. J. Fernández-Reiriz, and E. Navarro. 2016. Allometric size-scaling of biometric growth parameters and metabolic and excretion rates. A comparative study of intertidal and subtidal populations of mussels (*Mytilus galloprovincialis*). Hydrobiologia 772:261–275.
Artüz, M. L., O. B. Artüz, M. Sakınç, B. Yalçın, and B. E. Erdoğan. 2019. Fewer species but more existing individuals: Testing the hypothesis 'Pessimum Conditions Rule' based on long-term data of species composition of benthic fauna and environmental variables in the Sea of Marmara, Turkey. J. Environ. Toxicol. Stud. 3:1–18.
Astall, C. M., and M. B. Jones. 1991. Respiration and biometry in the sea cucumber *Holothuria forskali*. J. Mar. Biol. Assoc. United Kingdom 71:73–81. Monash University.
Atkins, D. 1959. The early growth stages and adult structure of the lophophore of *Macandrevia cranium* (Müller) (Brachiopoda, Dallinidae). J. Mar. Biol. Assoc. United Kingdom 38:335–350. Monash University.
Atwood, D. G. 1973. Larval development in the asteroid *Echinaster echinophorus*. Biol. Bull. 144:1–11.
Audouin, J. V, and H. Milne Edwards. 1833. Classification des Annélides et description de celles qui habitent les côtes de la France [Part 2]. Ann. des Sci. Nat. Paris 1:187–247.
Auld, J. R., W. Chester, P. Jarne, U. De Montpellier, and U. P. Valéry. 2016. Sex and Recombination in Snails. Pp. 49–60 *in* R. M. Kliman, ed. Encyclopedia of Evolutionary Biology. Elsevier.
Baba, K. 1957. The species of the genus *Elysia* from Japan. Publ. Seto Mar. Biol. Lab. 6:69–

74.
Baeta, M., E. Galimany, and M. Ramón. 2016. Growth and reproductive biology of the sea star *Astropecten aranciacus* (Echinodermata, Asteroidea) on the continental shelf of the Catalan Sea (northwestern Mediterranean). Helgol. Mar. Res. 70. BioMed Central.
Bailey, T. G., J. J. Torres, M. J. Youngbluth, and G. P. Owen. 1994. Effect of decompression on mesopelagic gelatinous zooplankton: A comparison of in situ and shipboard measurements of metabolism. Mar. Ecol. Prog. Ser. 113:13–28.
Baker, A. N. 1998. The rediscovery of *Echinus elevatus* Hutton 1872 in New Zealand, and a new name for *Acanthotrema* Baker and Rowe 1990 (Echinodermata: Echinoidea). J. R. Soc. New Zeal. 28:281–286.
Balaparameswara Rao, M. 1980. Studies on the oxygen consumption of a tropical intertidal limpet Cellana radiata (Born): Effect of body size and tidal rhythm. Hydrobiologia 71:175–179.
Båmstedt, U. 1979. Seasonal variation in the respiratory rate and ETS activity of deep-water zooplankton from the Swedish west coast. Cycl. Phenom. Mar. Plants Anim. 267–274.
Banse, K. 1979. Sabellidae (Polychaeta) principally from the northeast Pacific Ocean. J. Fish. Res. Board Canada 36:869–882.
Baptista, L. V. C. 2017. Phylogenetic analysis of the family Rissoidae (Mollusca : Gastropoda) in the Azores Archipelago (NE Atlantic) Phylogenetic analysis of the family Rissoidae (Mollusca : Gastropoda) in the Azores Archipelago (NE Atlantic). University of Porto.
Barker, M. F. 1978. Descriptions of the larvae of *Stichaster australis* (Verrill) and *Coscinasterias calamaria* (Gray) (Echinodermata: Asteroidea) from New Zealand, obtained from laboratory culture. Biol. Bull. 154:32–46.
Barker, M. F. 2013. Evechinus chloroticus. Elsevier.
Barnes, H., and M. Barnes. 1965. Egg size, nauplius size, and their variation with local, geographical, and specific factors in some common cirripedes. J. Anim. Ecol. 34:391.
Battaglene, S. C., J. E. Seymour, C. Ramofafia, and I. Lane. 2002. Spawning induction of three tropical sea cucumbers, *Holothuria scabra*, *H. fuscogilva* and *Actinopyga mauritiana*. Aquaculture 207:29–47.
Baumiller, T. K., and M. Labarbera. 1989. Metabolic rates of caribbean crinoids (Echinodermata), with special reference to deep-water stalked and stalkless taxa. Comp. Biochem. Physiol. -- Part A Physiol. 93:391–394.
Baums, I. B., M. W. Miller, and A. M. Szmant. 2003. Ecology of a corallivorous gastropod, *Coralliophila abbreviata*, on two seleractinian hosts. II. Feeding, respiration and growth. Mar. Biol. 142:1093–1101.
Baur, B. 1998. Sperm competition in molluscs. Pp. 255–305 *in* T. R. Birkhead and A. P. Møller, eds. Sperm Competition and Sexual Selection. Academic Press.
Bayne, B. L., C. J. Bayne, T. C. Carefoot, and R. J. Thompson. 1976. The physiological ecology of *Mytilus californianus* Conrad - 1. Metabolism and energy balance. Oecologia 22:211–228.
Bayne, B. L., and C. Scullard. 1977. Rates of nitrogen excretion by species of *Mytilus* (Bivalvia: Mollusca). J. Mar. Biol. Assoc. United Kingdom 57:355–369. Monash University.
Beaumont, A. R., and D. A. Barnes. 1992. Aspects of veliger larval growth and byssus drifting of the spat of *Pecten maximus* and *Aequipecten* (*Chlamys*) *opercularis*. ICES J. Mar. Sci. 49:417–423.
Beeman, R. D. 1977. Gastropoda: Opisthobranchia. Reprod. Mar. Invertebr. IV:115–179. ACADEMIC PRESS, INC.
Begum, S., L. Basova, J. Strahl, A. Sukhotin, O. Heilmayer, E. Philipp, T. Brey, and D.

Abele. 2009. A metabolic model for the ocean quahog *Arctica islandica* - effects of animal mass and age, temperature, salinity, and geography on respiration rate. J. Shellfish Res. 28:533–539.
Behrens, D. W., and A. Hermosillo. 2005. Eastern Pacific Nudibranchs: A guide to the Opisthobranchs from Alaska to Central America. Monterey, CA.
Beis, I., A. Manousis, and J. Barrett. 1980. Studies on the respiration of the polychaete *Ophelia bicornis*. Comp. Biochem. Physiol. 67A:303–305.
Ben-Eliahu, M. N., and H. A. Ten Hove. 1989. Redescription of *Rhodopsis pusilla* Bush, a little known but widely distributed species of Serpulidae (Polychaeta). Zool. Scr. 18:381–395.
Bentley, M. G., and K. Serries. 1992. Sperm ultrastructure in two species of the polychaete genus *Harmothoe* (Polynoidae). Helgoländer Meeresuntersuchungen 46:171–183.
Bergan, P. 1953. On the anatomy and reproduction biology in *Spiororbis* Daudin. Nytt Mag. Zool. 1:1–26.
Bergquist, D. C., C. Fleckenstein, E. B. Szalai, J. Knisel, and C. R. Fisher. 2004. Environment drives physiological variability in the cold seep mussel *Bathymodiolus childressi*. Limnol. Oceanogr. 49:706–715.
Berkeley, E., and C. Berkeley. 1954. Additions to the polychaete fauna of Canada, with comments on some older records. J. Fish. Res. Board Canada 11:454–471.
Berkman, P. A., T. R. Waller, and S. P. Alexander. 1991. Unprotected larval development in the Antarctic scallop *Adamussium colbecki* (Mollusca: Bivalvia: Pectinidae). Antarct. Sci. 3:151–157.
Berrill, N. J. 1977. Functional morphology and development of segmental inversion in sabellid polychaetes. Biol. Bull. 153:453–467.
Beu, A. G. 2012. Marine Mollusca of the last 2 million years in New Zealand. Part 5. Summary. J. R. Soc. New Zeal. 42:1–47.
Bhaud, M., and J. Duchêne. 1996. Change from planktonic to benthic development: is life cycle evolution an adaptive answer to the constraints of dispersal? Oceanol. Acta 19:335–346.
Bhaud, M. R. 1998. Species of *Spiochaetopterus* (Polychaeta, Chaetopteridae) in the Atlantic-Mediterranean biogeographic area. Sarsia 83:243–263.
Biggs, D. C. 1977. Respiration and ammonium excretion by open ocean gelatinous zooplankton. Limnol. Oceanogr. 22:108–117.
Biggs, D. C. 1982. Zooplankton excretion and NH4+ cycling in near-surface waters of the Southern Ocean. I. Ross sea, austral summer 1977-1978. Polar Biol. 1:55–67.
Birkeland, C., F.-S. Chia, and R. R. Strathmann. 1971. Development, substratum selection, delay of metamorphosis and growth in the seastar, *Mediaster aequalis* Stimpson. Biol. Bull. 141:99–108.
Bishop, J. D. D., and A. J. Pemberton. 2006. The third way: Spermcast mating in sessile marine invertebrates. Integr. Comp. Biol. 46:398–406.
Black, R., S. J. Turner, and M. S. Johnson. 1994. The early life history of *Bembicium vittatum* Philippi, 1846 (Gastropoda: Littorinidae). The Veliger 37:393–399.
Blake, E. A., and C. L. Van Dover. 2005. The reproductive biology of *Amathys lutzi*, an ampharetid polychaete from hydrothermal vents on the Mid-Atlantic Ridge. Invertebr. Biol. 124:254–264.
Blake, J. A. 2017a. Larval development of Polychaeta from the northern California coast. Fourteen additional species together with seasonality of planktic larvae over a 5-year period. J. Mar. Biol. Assoc. United Kingdom 97:1081–1133.
Blake, J. A. 1993. Life history analysis of five dominant infaunal polychaete species from the continental slope off North Carolina. J. Mar. Biol. Assoc. United Kingdom 73:123–141.

Monash University.
Blake, J. A. 2017b. Polychaeta orbiniidae from Antarctica, the Southern Ocean, the abyssal Pacific Ocean, and off South America.
Blake, J. A. 2009. Redescription of *Capitella capitata* (Fabricius) from West Greenland and designation of a neotype (Polychaeta, Capitellidae). Zoosymposia 2:55–80.
Blake, J. A. 1969. Reproduction and larval development of *Polydora* from Northern New England (Polychaeta: Spionidae). Ophelia 7:1–63.
Blake, J. A. 1971. Revision of the genus *Polydora* from the east coast of North America (Polychaeta: Spionidae). Smithson. Contrib. to Zool. 1–32.
Blake, J. A. 1975a. The larval development of Polychaeta from the northern California coast. I. *Cirriformia spirabrancha* (Family Cirratulidae). Trans. Am. Microsc. Soc. 94:179.
Blake, J. A. 1974. The larval development of polychaeta from the northern California coast. II. *Nothria elegans* (Family Onuphidae). Ophelia 13:43–61.
Blake, J. A. 1975b. The larval development of polychaeta from the northern California coast. III Eighteen species of Errantia.
Blake, J. A. 1980. The larval development of polychaeta from the northern California coast. IV. *Leitoscoloplos pugettensis* and *Scoloplos acmeceps* (Family Orbiniidae). Ophelia 19:1–18.
Blake, J. A., and P. L. Arnofsky. 1999. Reproduction and larval development of the spioniform Polychaeta with application to systematics and phylogeny. Hydrobiologia 402:57–106.
Blake, J. A., and K. H. Woodwick. 1975. Reproduction and larval development of *Pseudopolydora paucibranchiata* (Okuda) and *Pseudopolydora kempi* (Southern) (Polychaeta: Spionidae). Biol. Bull. 149:109–127.
Blankley, W. O. 1984. Ecology of the starfish Anasterias rupicola at Marion Island (Southern Ocean). Mar. Ecol. Prog. Ser. 18:131–137.
Bochert, R. 1997. *Marenzelleria viridis* (Polychaeta: Spionidae): A review of its reproduction. Aquat. Ecol. 31:163–175.
Bookhout, C. G. 1957. The development of *Dasybranchus caducus* (Grube) from the egg to the preadult. J. Morphol. 100:141–185.
Bookhout, C. G., and E. C. Horn. 1949. The development of *Axiothella mucosa* (Andrews). J. Morphol. 84:145–183.
Borges, J. C. S., J. R. M. C. Silva, A. J. S. Rocha, B. E. Jensch-Junior, L. N. Pressinotti, M. Passos, V. Gomes, P. C. Branco, and V. N. Phan. 2012. Energetic metabolic differences between tropical (*Lytechinus variegatus*) and polar (*Sterechinus neumayeri*) echinoderms. Pesqui. Antártica Bras. 5:71–79.
Borges, M., R. A. dos S. Alitto, and A. C. Z. Amaral. 2015. From baby to adult: ontogenetic series of nine species of Ophiuroidea from Atlantic Southwestern. Rev. Biol. Trop. 63:361–381.
Bork, E., and K. Kenton. 2017. *Aplysiopsis enteromorphae*. P. *in* T. C. Hiebert, B. A. Butler, and A. L. Shanks, eds. Oregon Estuarine Invertebrates: Rudys' Illustrated Guide to Common Species. University of Oregon Libraries and Oregon Institute of Marine Biology, Charleston, OR.
Bos, A. R., J. C. E. Alipoyo, L. T. Cardona, G. S. Gumanao, and F. N. Salac. 2008. Population structure of common Indo-Pacific sea stars in the Davao Gulf, Philippines. UPV J. Nat. Sci. 13:11–24.
Bos, A. R., G. S. Gumanao, B. Mueller, and M. M. Saceda. 2013. Size at maturation, sex differences, and pair density during the mating season of the Indo-Pacific beach star *Archaster typicus* (Echinodermata: Asteroidea) in the Philippines. Invertebr. Reprod. Dev. 57:113–119.

Bosch, I. 1989. Contrasting modes of reproduction in two Antarctic asteroids of the genus *Porania*, with a description of unusual feeding and non-feeding larval types. Biol. Bull. 177:77–82.
Bosch, I., K. A. Beauchamp, M. E. Steele, and J. S. Pearse. 1987. Development, metamorphosis, and seasonal abundance of embryos and larvae of the Antarctic sea urchin *Sterechinus eumayeri*. Biol. Bull. 173:126–135.
Bosch, I., and J. S. Pearse. 1990. Developmental types of shallow-water asteroids of McMurdo Sound, Antarctica.
Boudouresque, C. F., and M. Verlaque. 2013. *Paracentrotus lividus*. Pp. 297–327 *in* J. M. Lawrence, ed. Sea Urchins: Biology and Ecology. Elsevier.
Bourgoin, A., and M. Guillou. 1990. Variations in the reproductive cycle of *Acrocnida brachiata* (Echinodermata: Ophiuroidea) according to environment in the Bay of Douarnenez (Brittany). J. Mar. Biol. Assoc. United Kingdom 70:57–66. Monash University.
Boyden, C. R. 1972. The behaviour, survival and respiration of the cockles *Cerastoderma edule* and *C. glaucum* in air. J. Mar. Biol. Assoc. United Kingdom 52:661–680. Monash University.
Brafield, A. E. 1968. The oxygen consumption of an echiuroid, *Bonellia viridis* Rolando. J. Exp. Biol. 48:427–434.
Brand, A. R., and D. J. Morris. 1984. The respiratory responses of the dog cockle *Glycymeris glycymeris* (L.) to declining environmental oxygen tension. J. Exp. Mar. Bio. Ecol. 83:89–106.
Brey, T., C. Müller-Wiegmann, Z. M. C. Zittier, and W. Hagen. 2010. Body composition in aquatic organisms - A global data bank of relationships between mass, elemental composition and energy content. J. Sea Res. 64:334–340. Elsevier B.V.
Bright, M., and F. Lallie. 2010. The biology of vestimentiferan tubeworms. Pp. 213–265 *in* R. N. Gibson, R. J. A. Atkinson, and J. D. M. Gordon, eds. Oceanography and Marine Biology: An Annual Review. Taylor & Francis.
Britaev, T. A., L. A. Medvedeva, and V. I. Radashevsky. 1986. Reproduction and development of a symbiotic polychaete, *Arctonoe vittata* (Polynoidae), in the Vostok Bay of the Sea of Japan. Zool. Zhurnal 65:713–725.
Britayev, T. A. 1991. Life cycle of the symbiotic scaleworm *Arctonoe vittata* (Polychaeta: Polynoidae). Ophelia Suppl. 5:305–312.
Britz, P. J., T. Hecht, and S. Mangold. 1997. Effect of temperature on growth, feed consumption and nutritional indices of *Haliotis midae* fed a formulated diet. Aquaculture 152:191–203.
Brockington, S. 2001. The seasonal energetics of the Antarctic bivalve *Laternula elliptica* (King and Broderip) at Rothera Point, Adelaide Island. Polar Biol. 24:523–530.
Brockington, S., and L. S. Peck. 2001. Seasonality of respiration and ammonium excretion in the Antarctic echinoid *Sterechinus neumayeri*. Mar. Ecol. Prog. Ser. 219:159–168.
Brown, A. C., A. D. Ansell, and A. Trevallion. 1978. Oxygen consumption by *Bullia* (Dorsanum) *melanoides* (Deshayes) and *Bullia digitalis* Meuschen (Gastropoda, Nassaridae)-an example of non-acclimation. Comp. Biochem. Physiol. -- Part A Physiol. 61:123–125.
Brown, A. C., and F. M. da Silva. 1979. The effects of temperature on oxygen consumption in *Bullia digitalis meuschen* (Gastropoda, Nassaridae). Comp. Biochem. Physiol. -- Part A Physiol. 62:573–576.
Brown, N. P., and S. D. Eddy. 2015. Echinoderm aquaculture. John Wiley& Sons, Hoboken, NJ, USA.
Brown, W. I., and J. M. Shick. 1979. Bimodal gas exchange and the regulation of oxygen

uptake in holothurians. Biol. Bull. 156:272–288.
Brueggeman, P. 1998. Echinodermata - Asteroidea: seastars.
Buckland-Nicks, J., G. Gibson, and R. Koss. 2002. Phylum Mollusca: Polyplacophora, Aplacophora, Scaphopoda. Pp. 246–290 *in* C. M. Young, M. A. Sewell, and M. E. Rice, eds. Atlas of marine invertebrate larvae. Academic Press, San Diego, California.
Burcham, D., and N. L. Caruso. 2015. Abundance, size, and occurrence of *Arbacia stellata* in Orange County, California. Calif. Fish Game 101:184–187.
Burnell, O. W., B. D. Russell, A. D. Irving, and S. D. Connell. 2013. Eutrophication offsets increased sea urchin grazing on seagrass aused by ocean warming and acidification. Mar. Ecol. Prog. Ser. 485:37–46.
Buzhinskaja, G. N., L. L. Jørgensen, and L. L. Jørgensen. 1997. Redescription of *Trochochaeta carica* (Birula, 1897) (Polychaeta, Trochochaetidae) with notes on reproductive biology and larvae. Sarsia 82:69–75.
Bybee, D. R., J. H. Bailey-Brock, and C. S. Tamaru. 2006. Evidence for sequential hermaphroditism in *Sabellastarte spectabilis* (Polychaeta: Sabellidae) in Hawai'i. Pacific Sci. 60:541–547.
Byrne, M. 1991a. Life history traits of Caribbean ophiuroids that brood their young. P. 299 *in* Biology of Echinodermata. CRC.
Byrne, M. 1991b. Reproduction, development and population biology of the Caribbean ophiuroid *Ophionereis olivacea*, a protandric hermaphrodite that broods its young. Mar. Biol. 111:387–399.
Byrne, M. 1992. Reproduction of sympatric populations of *Patiriella gunnii*, </i>P. calcar</i> and *P. exigua* in New South Wales, asterinid seastars with direct development. Mar. Biol. 114:297–316.
Byrne, M., M. W. Hart, A. Cerra, and P. Cisternas. 2003. Reproduction and larval morphology of broadcasting and viviparous species in the *Cryptasterina* species complex. Biol. Bull. 205:285–294.
Byrne, M., F. W. E. Rowe, L. M. Marsh, and C. L. Mah. 2017. Class Asteroidea. Pp. 231–294 *in* T. D. O'Hara and M. Byrne, eds. Australian Echinoderms: Biology, Ecology and Evolution. CSIRO Publishing.
Calosi, P., S. P. S. Rastrick, C. Lombardi, H. J. de Guzman, L. Davidson, M. Jahnke, A. Giangrande, J. D. Hardege, A. Schulze, J. I. Spicer, and M. C. Gambi. 2013. Adaptation and acclimatization to ocean acidification in marine ectotherms: An in situ transplant experiment with polychaetes at a shallow CO2 vent system. Philos. Trans. R. Soc. B Biol. Sci. 368:1–15.
Calvo, M., J. Templado, and P. E. Penchaszadeh. 1998. Reproductive biology of the gregarious Mediterranean vermetid gastropod *Dendropoma petraeum*. J. Mar. Biol. Assoc. United Kingdom 78:525–549.
Cameron, J. L., and P. V Fankboner. 1989. Reproductive biology of the commercial sea cucumber *Parastichopus californicus* (Stimpson) ecology of development, recruitment, and the juvenile life stage. J. Exp. Mar. Bio. Ecol. 127:43–67.
Campbell, R. D. 1974. Cnidaria. Pp. 133–199 *in* Reproduction of Marine Invertebrates - Acoelomate and Pseudocoelomate Metazoans.
Cañete, J., and R. Ambler. 1992. Desarrollo intracapsular del mesogastrópodo comestible *Calyptraea* (*Trochita*) *trochiformis* (Born, 1778), en Chile. Rev. Chil. Hist. Nat. 65:255–266.
Capa, M., P. Hutchings, and R. Peart. 2012. Systematic revision of Sabellariidae (Polychaeta) and their relationships with other polychaetes using morphological and DNA sequence data.
Carey, N., S. Dupont, B. Lundve, and J. D. Sigwart. 2014. One size fits all: Stability of

metabolic scaling under warming and ocean acidification in echinoderms. Mar. Biol. 161:2131–2142.
Carey, N., A. Galkin, P. Henriksson, J. G. Richards, and J. D. Sigwart. 2013a. Variation in oxygen consumption among 'living fossils' (Mollusca: Polyplacophora). J. Mar. Biol. Assoc. United Kingdom 93:197–207.
Carey, N., and J. D. Sigwart. 2014. Size matters: Plasticity in metabolic scaling shows body-size may modulate responses to climate change. Biol. Lett. 10:1–4.
Carey, N., J. D. Sigwart, and J. G. Richards. 2013b. Economies of scaling: More evidence that allometry of metabolism is linked to activity, metabolic rate and habitat. J. Exp. Mar. Bio. Ecol. 439:7–14. Elsevier B.V.
Carpizo-Ituarte, E., and M. G. Hadfield. 1998. Stimulation of metamorphosis in the polychaete *Hydroides elegans* Haswell (Serpulidae). Biol. Bull. 194:14–24.
Carrasco, F. D. 1983. Description of adults and larvae of a new deep water species of *Hyalinoecia* (Polychaeta, Onuphidae) from the southeastern Pacific Ocean. J. Nat. Hist. 17:87–93.
Carrera-Parra, L. F. 2006. Revision of *Lumbrineris* de Blainville, 1828 (Polychaeta: Lumbrineridae). Zootaxa 1828:1–64.
Carson, H. S., and B. T. Hentschel. 2006. Estimating the dispersal potential of polychaete species in the Southern California Bight: Implications for designing marine reserves. Mar. Ecol. Prog. Ser. 316:105–113.
Carvalho, A. L. P. S., and C. R. R. Ventura. 2002. The reproductive cycle of *Asterina stellifera* (Möbius) (Echinodermata: Asteroidea) in the Cabo Frio region, southeastern Brazil. Mar. Biol. 141:947–954.
Castilla, J. C., and J. Cancino. 1976. Spawning behaviour and egg capsules of *Concholepas concholepas* (Mollusca: Gastropoda: Muricidae). Mar. Biol. 37:255–263.
Castritsi-Catharios, J., H. Miliou, and J. Pantelis. 2005. Experimental sponge fishery in Egypt during recovery from sponge disease.
Cazaux, C. 1972. Développement larvaire d'annélides polychètes (Bassin d'Arcachon). Arch. Zool. exp. gén. 113:71–108.
Cazaux, C. 1969. Etude morphologique du developpement larvaire d' annelides polychetes (Bassin d'Arcachon) II. Phyllodocidae, Syllidae, Nereidae. Arch. Zool. Exp. Gen. 110:145–202.
Cazaux, C. 1967. Larval development of *Glycera convoluta* Keferstein. Vie Milieu Ser. A-Biologie Mar. 18:559–571.
Cazaux, C. 1970. Recherches sur l'écologie et le développement larvaires des Polychètes de la région d'Arcachon. L'Université de Bordeaux.
Cellario, C., and L. Fenaux. 1990. *Paracentrotus lividus* (Lamarck) in culture (larval and benthic phases): Parameters of growth observed during two years following metamorphosis. Aquaculture 84:173–188.
Chaitanawisuti, N., and A. Kritsanapuntu. 2000. Growth and production of hatchery-reared juvenile spotted babylon *Babylonia areolata* Link 1807 cultured to marketable size in intensive flowthrough and semi-closed recirculating water systems. Aquac. Res. 31:415–419.
Chandramouli, K. H., D. Reish, H. Zhang, P. Y. Qian, and T. Ravasi. 2015. Proteomic changes associated with successive reproductive periods in male polychaetous *Neanthes arenaceodentata*. Sci. Rep. 5:1–10. Nature Publishing Group.
Chao, S. M., C. P. Chen, and P. S. Alexander. 1995. Reproductive cycles of tropical sea cucumbers (Echinodermata: Holothuroidea) in southern Taiwan. Mar. Biol. 122:289–295.
Chao, S. M., and C. C. Tsai. 1995. Reproduction and population dynamics of the fissiparous

brittle star *Ophiactis savignyi* (Echinodermata: Ophiuroidea). Mar. Biol. 124:77–83.
Charef, A., N. Zamouri Langar, and I. H. Gharsallah. 2012. Stock size assessment and spatial distribution of bivalve species in the Gulf of Tunis. J. Mar. Biol. Assoc. United Kingdom 92:179–186.
Charles, F., E. Jordana, J. M. Amouroux, A. Grémare, M. Desmalades, and L. Zudaire. 2003. Reproduction, recruitment and larval metamorphosis in the serpulid polychaete *Ditrupa arietina* (O.F. Müller). Estuar. Coast. Shelf Sci. 57:435–443.
Charlina, N. A., I. Y. Dolmatov, and I. C. Wilkie. 2009. Juxtaligamental system of the disc and oral frame of the ophiuroid *Amphipholis kochii* (Echinodermata: Ophiuroidea) and its role in autotomy. Invertebr. Biol. 128:145–156.
Chatzinikolaou, E., and C. A. Richardson. 2010. Parental size and environmental conditions affect egg capsule production by *Nassarius reticulatus* (Linnaeus 1758) (Gastropoda: Nassariidae). J. Exp. Mar. Bio. Ecol. 390:14–21. Elsevier B.V.
Cheung, S. G., and K. W. Fan. 1999. Thermal acclimation of respiration in the green mussel *Perna viridis* (L.). Asian Mar. Biol. 16:197–201.
Chevaldonne, P., and D. Jollivet. 1993. Videoscopic study of deep-sea hydrothermal vent alvinellid polychaete populations: biomass estimation and behaviour. Mar. Ecol. Prog. Ser. 95:251–262.
Chia, F.-S. 1965. Development of a deep-sea cushion star, *Pteraster tesselatus*. Proc. Calif. Acad. Sci. 34:505–510.
Chia, F.-S., L. R. McEdward, R. L. Miller, T. E. Schroeder, R. L. Shimek, S. T. Smiley, and R. R. Strathmann. 1987. Phylum Echinodermata Class Asteroidea. Pp. 535–555 *in* M. F. Strathmann, ed. Reproduction and Development of Marine Invertebrates of the Northern Pacific Coast. University of Washington Press, Seattle, WA.
Chia, F. 1971. Oviposition, fecundity, and larval development of three sacoglossan opisthobranchs from the Northumberland coast, England. Veliger 13:319--325.
Chia, F. S., and R. Koss. 1978. Development and metamorphosis of the planktotrophic larvae of *Rostanga pulchra* (Mollusca: Nudibranchia). Mar. Biol. 46:109–119.
Childress, J. J., A. J. Arp, and C. R. Fisher. 1984. Metabolic and blood characteristics of the hydrothermal vent tube-worm *Riftia pachyptila*. Mar. Biol. 83:109–124.
Chiva, M., N. Saperas, and E. Ribes. 2011. Complex chromatin condensation patterns and nuclear protein transitions during spermiogenesis: Examples from mollusks. Tissue Cell 43:367–376.
Choe, S. 1960. On the life history of the polychaete worm, *Diopatra neapolitana* Delle Chiaje. Bull. Japanese Soc. Sci. Fish. 26:430–437.
Choi, H. K., H. Kim, and S. M. Yoon. 2018. *Timarete posteria*, a new cirratulid species from Korea (Annelida, Polychaeta, Cirratulidae). Zookeys 2018:1–15.
Christensen, A. B., and J. M. Colacino. 2000. Respiration in the burrowing brittlestar, *Hemipholis elongata* Say (Echinodermata, Ophiuroidea): A study of the effects of environmental variables on oxygen uptake. Comp. Biochem. Physiol. - A Mol. Integr. Physiol. 127:201–213.
Christie, G. 1985. A comparative study of the reproductive cycles of three Northumberland populations of *Chaetozone setosa* (Polychaeta: Cirratulidae). J. Mar. Biol. Assoc. United Kingdom 65:239–254. Monash University.
Christie, G. 1986. Observations on the reproductive biology of *Trichobranchus glacialis* Malmgren, 1866 (Polychaeta: Trichobranchidae). Sarsia 71:259–263.
Christie, G. 1984. The reproductive biology of a Northumberland population of *Sphaerodorum gracilis* (rathke, 1843) (polychaeta: Sphaerodoridae). Sarsia 69:117–121.
Christie, G. 1982. The reproductive cycles of two species of *Pholoe* (Polychaeta: Sigalionidae) off the Northumberland coast. Sarsia 67:283–292.

Chu, J.-W., and L. A. Levin. 1989. Photoperiod and temperature regulation of growth and reproduction in *Streblospio benedicti* (Polychaeta: Spionidae). Invertebr. Reprod. Dev. 15:131–142.

Chuang, S. H. 1977. Larval development in *Discinisca* (inarticulate brachiopod). Integr. Comp. Biol. 17:39–53.

Chughtai, I. 1986. Fine structure of spermatozoa in *Perkinsiana rubra* and *Pseudopotamilla reniformis* (Sabellidae: Polychaeta). Acta Zool. 67:165–171.

Chung, I. F., Y. M. Huang, T. H. Lee, and L. L. Liu. 2010. Reproduction of the bath sponge *Spongia ceylonensis* (Dictyoceratida: Spongiidae) from Penghu, Taiwan. Zool. Stud. 49:601–607.

Çinar, M. E., K. Fauchald, and E. Dagli. 2014. Occurrence of *Diopatra marocensis* (Annelida, Onuphidae) in the eastern Mediterranean. Zookeys 11:1–11.

Cipriani, R., and P. Penchaszadeh. 1993. How does *Strombina* reproduce? evidence from two Venezuelan species (Prosobranchia: Columbellidae). The Veliger 36:178–184.

Cisternas, P. A., and M. Byrne. 2005. Evolution of abbreviated development in the ophiuroid *Ophiarachnella gorgonia* involves heterochronies and deletions. Can. J. Zool. 83:1067–1078.

Clark, A. H. 1951. The brittle-stars of the United States Navy Antarctic Expedition 1947-48. J. Washingt. Acad. Sci. 41:26–30.

Clark, A. H. 1950. The feather-stars, sea-urchins, and sea-stars of the United States Navy Antarctic Expedition 1947-48. J. Washingt. Acad. Sci. 40:335–337.

Clark, H. L. 1904. The echinoderms of the Woods Hole region. Fish. Bull. Fish Wildl. Serv. United States 22:545–576.

Clark, K. B. 1975a. Nudibranch life cycles in the Northwest Atlantic and their relationship to the ecology of fouling communities. Helgoländer Wissenschaftliche Meeresuntersuchungen 27:28–69.

Clark, K. B. 1975b. Nudibranch life cycles in the Northwest Atlantic and their relationship to the ecology of fouling communities. Helgoländer Wissenschaftliche Meeresuntersuchungen 27:28–69.

Clark, K. B., and D. R. Franz. 1969. Occurrence of the sacoglossan opisthobranch *Hermaea dendritica* Alder & Hancock in New England. Veliger 12:174–175.

Clark, K. B., and A. Goetzfried. 1978. Zoogeographic influences on development patterns of North Atlantic ascoglossa and nudibranchia, with a discussion of factors affecting egg size and number. J. Molluscan Stud. 44:283–294.

Clark, K. B., and K. R. Jensen. 1981. A comparison of egg size, capsule size, and development patterns in the order Ascoglossa (Sacoglossa) (Mollusca: Opisthobranchia). Int. J. Invertebr. Reprod. 3:57–64.

Clausen, L. K. B., K. N. Andersen, T. L. Hygum, A. Jørgensen, and N. Møbjerg. 2014. First record of cysts in the tidal tardigrade *Echiniscoides sigismundi*. Helgol. Mar. Res. 68:531–537.

Cloney, R. A. 1987. Phylum Urochordata, Class Ascidiacea. Pp. 607–639 *in* M. F. Strathmann, ed. Reproduction and Development of Marine Invertebrates of the Northern Pacific Coast. University of Washington Press, Seattle, WA.

Coe, W. R. 1949. Divergent methods of development in morphologically similar species of prosobranch gastropods. J. Morphol. 84:383–399.

Coe, W. R. 1948. Nutrition and sexuality in protandric gastropods of the genus *Crepidula*. Biol. Bull. 94:158–160.

Coe, W. R. 1938. Sexual phases in the gastropod *Crucibulum spinosum*. J. Morphol. 63:345–361.

Collin, R. 2004. Phylogenetic effects, the loss of complex characters, and the evolution of

development in calyptraeid gastropods. Evolution. 58:1488–1502.
Collin, R. 2013. Phylogenetic patterns and phenotypic plasticity of molluscan sexual systems. Integr. Comp. Biol. 53:723–735.
Collin, R. 2010. Repeatability of egg size in two marine gastropods: brood order and female size do not contribute to intraspecific variation. Mar. Ecol. Prog. Ser. 410:89–96.
Collin, R. 2000. Sex change, reproduction, and development of *Crepidula adunca* and *Crepidula lingulata* (Gastropoda: Calyptraeidae). Veliger 43:24–33.
Collin, R. 2003a. Worldwide patterns in mode of development in calyptraeid gastropods. Mar. Ecol. Prog. Ser. 247:103–122.
Collin, R. 2003b. Worldwide patterns in mode of development in calyptraeid gastropods. Mar. Ecol. Prog. Ser. 247:103–122.
Conand, C. 1993. Reproductive biology of the holothurians from the major communities of the New Caledonian Lagoon. Mar. Biol. Int. J. Life Ocean. Coast. Waters 116:439–450.
Conand, C. 1981. Sexual cycle of three commercially important holothurian species (Echinodermata) from the lagoon of New Caledonia. Bull. Mar. Sci. 31:523–543.
Coppard, S. E. 2008. A comparative analysis of the spatangoid echinoid genera *Brissopsis* and *Metalia*: A new genus and species of spatangoid (Echinodermata: Echinoidea: Brissopsidae) from the Philippines and the reassignment of *Brissopsis persica* to <i>Met. Zootaxa 1–23.
Cordes, E. E., D. C. Bergquist, M. L. Redding, and C. R. Fisher. 2007. Patterns of growth in cold-seep vestimenferans including *Seepiophila jonesi*: A second species of long-lived tubeworm. Mar. Ecol. 28:160–168.
Cottin, D., J. Ravaux, N. Léger, S. Halary, J. Y. Toullec, P. M. Sarradin, F. Gaill, and B. Shillito. 2008. Thermal biology of the deep-sea vent annelid *Paralvinella grasslei*: In vivo studies. J. Exp. Biol. 211:2196–2204.
Coulon, P., M. Jangoux, and P. Bulteel. 1992. Respiratory rate and assessment of secondary production in the *Holothuroid Holothuria tubulosa* (Echinodermata) from Mediterranean seagrass beds. Mar. Ecol. 13:63–68.
Cragg, S. M. 2016. Biology and Ecology of Scallop Larvae. Pp. 31–83 *in* S. E. Shumway and G. J. Parsons, eds. Developments in Aquaculture and Fisheries Science. Elsevier B.V.
Creese, R. G. 1980. Reproductive cycles and fecundities of two species of *Siphonaria* (Mollusca: Pulmonata) in South-Eastern Australia. Mar. Freshw. Res. 31:37–47.
Crisp, M. 1977. The development of the serpulid *Pomatoleios kraussii* (Annelida, Polychaeta). J. Zool. 183:147–160.
Crisp, M., J. Davenport, and S. E. Shumway. 1978. Effects of feeding and of chemical stimulation on the oxygen uptake of *Nassarius reticulatus* (Gastropoda: Prosobranchia). J. Mar. Biol. Assoc. United Kingdom 58:387–399.
Crump, R. G., and M. F. Barker. 1985. Sexual and asexual reproduction in geographically separated populations of the fissiparous asteroid *Coscinasterias calamaria* (Gray). J. Exp. Mar. Bio. Ecol. 88:109–127.
Crump, R. G., and R. H. Emson. 1978. Some aspects of the population dynamics of *Asterina gibbosa* (Asteroidea). J. Mar. Biol. Assoc. United Kingdom 58:451–466.
Crumrine, L. 2001. Polychaeta. Pp. 39–76 *in* A. L. Shanks, ed. ldentification Guide to Larval Marine Invertebrates of the Pacific Northwest. Oregon Institute of Marine Biology.
Cruz, R. A., and A. V Giusti. 1990. Intracapsular development of *Crepidula marginalis* (Gastropoda: Calyptraeidae). Rev. Biol. Trop. 38:289–294.
Curtis, M. A. 1977. Life cycles and population dynamics of marine benthic polychaetes from the Disko Bay area of West Greenland. Ophelia 16:9–58.
Dales, R. P. 1951a. Notes on the reproduction and early development of the cirratulid *Tharyx marioni* (St Joseph). J. Mar. Biol. Assoc. United Kingdom 30:113–117.

Dales, R. P. 1951b. Observations on the structure and life history of *Autolytus prolifer* (O.F. Müller). J. Mar. Biol. Assoc. United Kingdom 30:119–128.
Dales, R. P. 1961. Oxygen uptake and irrigation of the burrow by three terebellid polychaetes: *Eupolymnia*, *Thelepus*, and *Neoamphitrite*. Phys. Zool. 34:306–311.
Dales, R. P. 1952. The larval development and ecology of *Thoracophelia mucronate* (Treadwell). Biol. Bull. 102:232–242.
Dales, R. P., C. P. Mangum, and J. C. Tichy. 1970. Effects of changes in oxygen and carbon dioxide concentrations on ventilation rhythms in onuphid polychaetes. J. Mar. Biol. Assoc. United Kingdom 50:365–380. Monash University.
Dall, W., B. J. Hill, P. C. Rothlisberg, and D. J. Sharples. 1990. The Biology of Penaeidae. Ch. 7 - Reproduction. Academic Press, London, England.
Daly, J. M. 1972. The maturation and breeding biology of *Harmothoë imbricata* (Polychaeta: Polynoidae). Mar. Biol. 12:53–66.
Dangott, L. J., and R. C. Terwilliger. 1986. The role of extracellular hemoglobins in the oxygen consumption of the burrowing polychaete, *Euzonus mucronata* (Treadwell). J. Exp. Mar. Bio. Ecol. 97:193–204.
Dartnall, A. J. 1970. Some species of *Asternia* from Flinders, Victoria. Vic. Nat. 87:19–22.
Dartnall, A. J., M. Byrne, J. Collins, and M. W. Hart. 2003. A new viviparous species of asterinid (Echinodermata, Asteroidea, Asterinidae) and a new genus to accommodate the species of pantropical exiguoid sea stars. Zootaxa 359:1.
Davenport, J., and E. R. Trueman. 1985. Oxygen uptake and buoyancy in zooplanktonic organisms from the tropical eastern atlantic. Comp. Biochem. Physiol. -- Part A Physiol. 81:857–863.
Day, J. H. 1934. Development of *Scolecolepis fuliginosa* (Claparède). J. Mar. Biol. Assoc. United Kingdom 19:633–654.
Day, R. L., and J. A. Blake. 1979. Reproduction and larval development of *Polydora giardi* Mesnil (Polychaeta: Spionidae). Biol. Bull. 156:20–30.
Dayton, P. K., G. A. Robilliard, R. T. Paine, and L. B. Dayton. 1974. Biological accommodation in the benthic community at McMurdo Sound, Antarctica. Ecol. Monogr. 44:105–128.
De Cubber, L., S. Lefebvre, T. Lancelot, L. Denis, and S. M. Gaudron. 2019. Annelid polychaetes experience metabolic acceleration as other Lophotrochozoans: Inferences on the life cycle of *Arenicola marina* with a Dynamic Energy Budget model. Ecol. Modell. 411.
de Jesús-Flores, C., S. A. Salazar-González, and S. I. Salazar-Vallejo. 2016. Morphological distinction between estuarine polychaetes: *Laeonereis culveri* and *L. nota* (Phyllodocida: Nereididae). Rev. Biol. Trop. 64:189–201.
De Jorge, F. B., and J. A. Petersen. 1968. Sex differences and maturation influence on the chemical composition in *Chaetopterus variopedatus* Rénier 1804 (polychaeta). Comp. Biochem. Physiol. 27.
de Silva, H. 1967. Studies on the biology of Spirorbinae (Polychaeta). J. Zool. 152:269–279.
Dean, D., and J. A. Blake. 1966. Life-history of *Boccardia hamata* (Webster) on the east and west coasts of North America. Biol. Bull. 130:316–330.
Debelius, H. 2001. Nudibranchs and sea snails: Indo-Pacific field guide. 3rd ed. Unterwasserarchiv, IKAN, Germany.
DeFreese, D. E., and K. B. Clark. 1983. Analysis of reproductive energetics of Florida Opisthobranchia (Mollusca: Gastropoda). Int. J. Invertebr. Reprod. 6:1–10.
Dehnel, P. A. 1955. Rates of growth of gastropods as a function of latitude. Physiol. Zool. 28:115–144.
Dehnel, P. A., and D. C. Kong. 1979. The effect of temperature on developmental rates in the

nudibranch *Cadlina luteomarginata*. Can. J. Zool. 57:1835–1844.
Delroisse, J., D. Fourgon, and I. Eeckhaut. 2013. Reproductive cycles and recruitment in *Ophiomastix venosa* and *Ophiocoma scolopendrina*, two co-existing tropical ophiuroids from the barrier reef of Toliara (Madagascar). Cah. Biol. Mar. 54:593–603.
Demaintenon, M. J. 2001. Analysis of reproductive system ontogeny and homology in *Nassarius vibex* (Gastropoda: Buccinidae: Nassariinae). J. Molluscan Stud. 67:37–49.
Desai, B. N. 1966. The biology of *Monodonta lineata* (da Costa). Proc. Malacol. Soc. London 37:1–17.
Desbruyères, D., P. Chevaldonné, A. M. Alayse, D. Jollivet, F. H. Lallier, C. Jouin-Toulmond, F. Zal, P. M. Sarradin, R. Cosson, J. C. Caprais, C. Arndt, J. O'Brien, J. Guezennec, S. Hourdez, R. Riso, F. Gaill, L. Laubier, and A. Toulmond. 1998. Biology and ecology of the "Pompeii worm" (*Alvinella pompejana* Desbruyeres and Laubier), a normal dweller of an extreme deep-sea environment: A synthesis of current knowledge and recent developments. Deep. Res. Part II Top. Stud. Oceanogr. 45:383–422.
Deshmukh, R. S. 1979. On the oxygen consumption of the estuarine mollusc *Meretrix meretrix* under various conditions. J. Mar. Biol. Assoc. India 21:1–9.
Devaney, D. M. 1970. Studies on ophiocomid brittlestars. I. A new genus (*Clarkcoma*) of Ophiocominae with a reevaluation of the genus *Ophiocoma*. Smithson. Contrib. to Zool. 1–41.
Dirnberger, J. M. 1993. Dispersal of larvae with a short planktonic phase in the polychaete *Spirorbis spirillum* (Linnaeus). Bull. Mar. Sci. 52:898–910.
Dixon, D. R. 1981. Reproductive biology of the serpulid *Ficopomatus* (*Mercierella*) *enigmaticus* in the Thames Estuary, S.E. England. J. Mar. Biol. Assoc. United Kingdom 61:805–815.
Doi, T. E. R. U. O. 1976. Some aspects of feeding ecology of the sea stars, genus *Astropecten*. Publ. from Amakusa Mar. Biol. Lab. 4:1–19.
Dong, Y., S. Dong, X. Tian, F. Wang, and M. Zhang. 2006. Effects of diel temperature fluctuations on growth, oxygen consumption and proximate body composition in the sea cucumber *Apostichopus japonicus* Selenka. Aquaculture 255:514–521.
Donnelly, J., H. Kawall, S. P. Geiger, and J. J. Torres. 2004. Metabolism of Antarctic micronektonic crustacea across a summer ice-edge bloom: Respiration, composition, and enzymatic activity. Deep. Res. Part II Top. Stud. Oceanogr. 51:2225–2245.
Dons, C. 1933. Om vekst og forplanting hos *Miroserpula inflata*. Det K. Nor. Vidensk. Selsk. Forh. Trondhjem 6:35–37.
Dornellas, A. P. S. 2012. Description of a new species of *Calliostoma* (Gastropoda, Calliostomatidae) from Southeastern Brazil. Zookeys 224:89–106.
Dorsett, D. A. 1961. The reproduction and maintenance of *Polydora ciliata* (Johnst.) at Whitstable. J. Mar. Biol. Assoc. United Kingdom 41:383–396. Monash University.
Dorsey, J. 1978. A new species of Syllides (Polychaeta: Syllidae) with notes on *Amblyosyllis speciosa* Izuka from San Clemente Island, California. Bull. South. Calif. Acad. Sci. 77:22–27.
Downey, M. E. 1986. Revision of the Atlantic Brisingida (Echinodermata:Asteroidea), with description of a new genus and family. Smithson. Contrib. to Zool. 1–57.
Drumm, D. T., K. P. Maslenikov, R. Van Syoc, J. W. Orr, R. R. Lauth, D. E. Stevenson, and T. W. Pietsch. 2016. An annotated checklist of the marine macroinvertebrates of Alaska. NOAA Prof. Pap. NMFS 19:289.
Dualan, I. V, and J. D. Williams. 2011. Palp growth, regeneration, and longevity of the obligate hermit crab symbiont Dipolydora commensalis (Annelida: Spionidae). Invertebr. Biol. 130:264–276.
Duchêne, J. C. 1984. Reproductive biology of *Boccardia polybranchia* (Carazzi) in

Kerguelen (Subantarctic Province). Polar Biol. 2:251–257.
Dupont, S., W. Thorndyke, M. C. Thorndyke, and R. D. Burke. 2009. Neural development of the brittlestar *Amphiura filiformis*. Dev. Genes Evol. 219:159–166.
Dye, A. H. 1991. Feed preferences of *Nucella crassilabrum* and juvenile *Concholepas concholepas* (gastropoda: Muricidae) from a rocky shore in Southern Chile. J. Molluscan Stud. 57:301–307.
Dye, A. H. 1979. The effect of acute and long term temperature changes on the respiration of two sand-dwelling bivalves. Comp. Biochem. Physiol. -- Part A Physiol. 63:405–409.
Dye, A. H., and L. McGwynne. 1980. The effect of temperature and season on the respiratory rates of three psammolittoral gastropods. Comp. Biochem. Physiol. -- Part A Physiol. 66:107–111.
Eckelbarger, K. J. 1977a. californica from southern California (Polychaeta: Sabellariidae), with a key to the sabellariid larvae of Florida and a review of development in the family. Bull. Mar. Sci. 27:241–255.
Eckelbarger, K. J. 1977b. Larval development of *Sabellaria floridensis* from Florida and *Phragmatopoma californica* from southern California (Polychaeta: Sabellariidae), with a key to the sabellariid larvae of Florida and a review of the development in the family. Bull. Mar. Sci. 27:241–255.
Eckelbarger, K. J. 1974. Population biology and larval development of the terebellid polychaete *Nicolea zostericola*. Mar. Biol. 27:101–113.
Eckelbarger, K. J., and J. P. Grassle. 1983. Ultrastructural differences in the eggs and ovarian follicle cells of *Capitella* (Polychaeta) sibling species. Biol. Bull. 165:379–393.
Eckelbarger, K. J., and S. A. Rice. 1988. Ultrastructure of oogenesis in the holopelagic polychaetes *Rhynchonerella angelini* and *Alciopa reynaudii* (Polychaeta: Alciopidae). Mar. Biol. 98:427–439.
Eckelbarger, K. J., and C. M. Young. 1994. Reproduction, Larval Biology, and Recruitment of the Deep-Sea Benthos. Columbia University Press.
Eckelbarger, K. J., and C. M. Young. 1999. Ultrastructure of gametogenesis in a chemosynthetic mytilid bivalve (*Bathymodiolus childressi*) from a bathyal, methane seep environment (northern Gulf of Mexico). Mar. Biol. 135:635–646.
Eckelbarger, K. J., C. M. Young, E. Ramirez Llodra, S. Brooke, and P. Tyler. 2001. Gametogenesis, spawning behavior, and early development in the “iceworm” *Hesiocaeca methanicola* (Polychaeta: Hesionidae) from methane hydrates in the Gulf of Mexico. Mar. Biol. 138:761–775.
Edmonds, S., K. Fauchald, C. Glasby, M. Grygier, P. Hutchings, H. Paxton, G. Rouse, E. Southward, C. Watson Russel, and R. Wilson. 2000. Class Polychaeta. Pp. 2–420 *in* Polychaetes and Allies: The Southern Synthesis. Fauna of Australia.
Edmonds, S. J. 2000a. Phylum Echiura. Pp. 2–30 *in* Polychaetes and Allies: The Southern Synthesis. Fauna of Australia.
Edmonds, S. J. 2000b. Phylum Sipuncula. Pp. 2–37 *in* Polychaetes and Allies: The Southern Synthesis. Fauna of Australia.
Eeckhaut, I., and M. Jangoux. 1993. Life cycle and mode of infestation of *Myzostoma cirriferum* (Annelida), a symbiotic myzostomid of the comatulid crinoid *Antedon bifida* (Echinodermata). Dis. Aquat. Organ. 15:207–217.
El-Bawab, F. 2020a. Phylum Crustacea, Pennant (1777). Pp. 475–711 *in* P. Gonzalez, ed. Invertebrate Embryology and Reproduction. Academic Press Inc., London, UK.
El-Bawab, F. 2020b. Phylum Porifera. Pp. 116–139 *in* P. Gonzalez, ed. Invertebrate Embryology and Reproduction. Elsevier Inc., London, UK.
Elbarhoumi, M., P. Scaps, C. Djediat, and F. Zghal. 2014. Ultrastructural study of oogenesis in *Marphysa sanguinea* (Annelida: Polychaeta: Eunicida) from the Lagoon of Tunis.

Sci. Mar. 78:99–113.
Eleaume, M., N. Ameziane, T. Baumiller, J.-P. Feral, and B. David. 2003. Development mode, egg size, larval size and some evolutionary considerations in comatulids (Crinoidea; Echinodermata). Pp. 307–315 *in* Echinoderm research 2001: Proceedings of the Sixth European Conference on Echinoderm Research. Banyuls-sur-Mer, France.
Emerson, C. W., T. E. Minchinton, and J. Grant. 1988. Population structure, biomass, and respiration of *Mya arenaria* L. on temperate sandflat. J. Exp. Mar. Bio. Ecol. 115:99–111.
Emerson, R. R. 1975. The biology of a population of *Diopatra ornata* at Santa Catalina Island, California. University of Southern California.
Emig, C. C. 1997. Ecology of the inarticulated brachiopods. Pp. 473–525 *in* R. L. Kaesler, ed. Treatise on invertebrate paleontology. Part H. Brachiopoda revised. The Geological Society of America, Inc., Lawrence, Kansas.
Emlet, R. B. 1995. Developmental mode and species geographic range in regular sea urchins (Echinodermata: Echinoidea). Evolution. 49:476.
Emlet, R. B. 2006. Direct development of the brittle star *Amphiodia occidentalis* (Ophiuroidea, Amphiuridae) from the northeastern Pacific Ocean. Invertebr. Biol. 125:154–171.
Emlet, R. B., L. R. McEdward, and R. R. Strathmann. 1987. Echinoderm larval ecology viewed from the egg. Pp. 55–136 *in* M. Jangoux and J. M. Lawrence, eds. Echinoderm Studies. A. A. Balkema Publishers, Rotterdam, Netherlands.
Emson, R. H., and R. G. Crump. 1979. Description of a new species of *Asterina* (Asteroidea), with an account of its ecology. J. Mar. Biol. Assoc. United Kingdom 59:77–94. Monash University.
Emson, R. H., C. M. Young, and G. L. J. Paterson. 1993. A fire worm with a sheltered life: studies of *Benthoscolex cubanus* Hartman (Amphinomidae), an internal associate of the bathyal sea-urchin *Archeopneustes hystrix* (A. Agassiz, 1880). J. Nat. Hist. 27:1013–1028.
Engstrom, N. A. 1982. Brooding behaviour and reproductive biology of a subtidal Puget Sound sea cucumber, *Cucumaria lubrica* (Clark, 1901) (Echinodermata: Holothuroidea). Pp. 447–450 *in* Echinoderms: Proceedings of the International Conferencex. Balkema, A, Tampa Bay.
Ereskovsky, A. V. 2000. Reproduction cycles and strategies of the cold-water sponges *Halisarca dujardini* (Demospongiae, Halisarcida), *Myxilla incrustans* and *Iophon piceus* (Demospongiae, Poecilosclerida) from the White Sea. Biol. Bull. 198:77–87.
Ereskovsky, A. V., and D. B. Tokina. 2007. Asexual reproduction in homoscleromorph sponges (Porifera; Homoscleromorpha). Mar. Biol. 151:425–434.
Ereskovsky, A. V. 2010. The Comparative Embryology of Sponges. Springer Science and Business Media B.V., New York, USA.
Estcourt, I. N. 1966. The life history and breeding biology of *Nicon aestuariensis* Knox (Annelida, Polychaeta). Trans. R. Soc. New Zealand, Zool. 7:179–194.
Etter, R. J. 1989. Life history variation in the intertidal snail *Nucella lapillus* across a wave-exposure gradient. Ecology 70:1857–1876.
Eyster, L. S. 1979. Reproduction and developmental variability in the opisthobranch *Tenellia pallida*. Mar. Biol. 51:133–140.
Fadlaoui, S., J. P. Lechapt, and C. Retiere. 1995. Larval development of the onuphid *Diopatra marocensis* (annelida: Polychaeta) from the Atlantic coast of Morocco. J. Mar. Biol. Assoc. United Kingdom 75:957–966. Monash University.
Fahey, S. J., and A. R. Carroll. 2007. Natural products isolated from species of Halgerda Bergh, 1880 (Mollusca: Nudibranchia) and their ecological and evolutionary

implications. J. Chem. Ecol. 33:1226–1234.
Falconetti, C., D. Fredj-Reygrobellet, and G. Fredj. 1977. Induction of gamete shedding and 1st stages of larval development in fissiparous asteroid *Schlerasterias richardi*. Mar. Biol. 39:171–178.
Falk-Petersen, I. B., and J. R. Sargent. 1982. Reproduction of asteroids from Balsfjorden, Northern Norway: Analyses of lipids in the gonads of *Ctenodiscus crispatus*, *Asterias lincki* and *Pteraster militaris*. Mar. Biol. 69:291–298.
Falkner, I., S. Barbosa, and M. Byrne. 2013. Reproductive biology of four ophiocomid ophiuroids in tropical and temperate Australia-reproductive cycle and oogenic strategies in species with different modes of development. Invertebr. Reprod. Dev. 57:189–199.
Falkner, I., M. Byrne, and M. A. Sewell. 2006. Maternal provisioning in *Ophionereis fasciata* and *O. schayeri*: Brittle stars with contrasting modes of development. Biol. Bull. 211:204–207.
Farfan, B. C., and F. B. Ramirez. 1988. Spawning and ontogeny of *Bulla gouldiana* (Gastropoda: Opisthobranchia: Cephalaspidea). The Veliger 31:114–119.
Farke, H., and E. M. Berghuis. 1979. Spawning, larval development and migration of *Arenicola marina* under field conditions in the western Wadden Sea. Netherlands J. Sea Res. 13:529–535.
Fauchald, K. 1982a. Description of *Mooreonuphis jonesi* new species of onuphid polychaete from shallow water in Bermuda with comments on variability and population ecology. Proc. Biol. Soc. Washingt. 95:807–825.
Fauchald, K. 1983a. Life diagram patterns in benthic polychaetes. Proc. Biol. Soc. Washingt. 96:160–177.
Fauchald, K. 1982b. Revision of *Onuphis*, *Nothria*, and *Paradiopatra* (Polychaeta: Onuphidae) based upon type material. Smithson. Contrib. to Zool. 356:120.
Fauchald, K. 1983b. Size increase in eunicean polychaetes. P. 964 *in* American Zoologist.
Fell, P. E. 1974. Porifera. Pp. 51–132 *in* A. C. Giese and J. S. Pearse, eds. Reproduction of Marine Invertebrates. John Hopkins University Press, New York, USA.
Fenaux, L. 1970. Maturation of the gonads and seasonal cycle of the planktonic larvae of the ophiuroid Amphiura chiajei Forbes. Biol. Bull. 138:262–271.
Feng, W., N. Nakabayashi, K. Narita, E. Inomata, M. N. Aoki, and Y. Agatsuma. 2019. Reproduction and population structure of the sea urchin *Heliocidaris crassispina* in its newly extended range: The Oga Peninsula in the Sea of Japan, northeastern Japan. PLoS One 14:e0209858.
Féral, J.-P., and P. Magniez. 1988. Relationship between rates of oxygen consumption and somatic and gonadal size in the subantarctic echinoid *Abatus cordatus* from Kerguelen. 6th Int. Echinoderm Conf. 581–587.
Fernald, R. L., M. G. Hadfield, S. C. Kempf, N. McLean, and S. V Millen. 1987. Phylum Mollusca, Class Gastropoda, Subclass Opisthobranchia. Pp. 268–302 *in* M. F. Strathmann, ed. Reproduction and Development of Marine Invertebrates of the Northern Pacific Coast. University of Washington Press, Seattle, WA.
Ferrand, J. G., C. Vadon, D. Doumenc, and A. Guille. 1988. The effect of depth on the reproductive cycle of *Brissopsis lyrifera* (Echinoidea, Echinodermata) in the Gulf of Lions, Mediterranean Sea. Mar. Biol. 99:387–392.
Fischer, A. 1999. Reproductive and developmental phenomena in annelids: A source of exemplary research problems. Hydrobiologia 402:1–20.
Fish, J. D., and S. Fish. 2011. A Student's Guide to the Seashore. 3rd ed. Cambridge University Press, Cambridge, UK.
Fisher, C. R., W. K. Fitt, and R. K. Trench. 1985. Photosynthesis and respiration in *Tridacna gigas* as a function of irradiance and size. Biol. Bull. 169:230–245.

Fisher, W. K. 1940. Asteroidea.
Fitt, W. K., and R. K. Trench. 1981. Spawning, development, and acquisition of zooxanthellae by *Tridacna squamosa* (Mollusca, Bivalvia). Biol. Bull. 161:213–235.
Flagor, T. E., and P. E. Bourdeau. 2018. First record of the predatory snail *Acanthinucella spirata* (Blainville, 1832) north of its known range. Mar. Biodivers. Rec. 11. Marine Biodiversity Records.
Fonseca, J. G., F. Laranjeiro, D. B. Freitas, I. B. Oliveira, R. J. M. Rocha, J. Machado, M. Hinzmann, C. M. Barroso, and S. Galante-Oliveira. 2020. Impairment of swimming performance in *Tritia reticulata* (L.) veligers under projected ocean acidification and warming scenarios. Sci. Total Environ. 731:139187. The Authors.
Foster, G. G., and A. N. Hodgson. 1995. Annual reproductive cycles of three sympatric species of intertidal holothurians (Echinodermata) from the coast of the Eastern Cape Province of South Africa. Invertebr. Reprod. Dev. 27:49–59.
Foster, N. M. 1971. Spionidae (Polychaeta) of the Gulf of Mexico and the Caribbean Sea. Stud. Fauna Curacao other Caribb. Islands 36:1–183.
Fourgon, D., I. Eeckhaut, D. Vaitilingon, and M. Jangoux. 2005. Lecithotrophic development and metamorphosis in the Indo-West Pacific brittle star *Ophiomastix venosa* (Echinodermata : Ophiuroidea). Invertebr. Reprod. Dev. 47:155–165.
Fox, H. M. 1936. The activity and metabolism of poikilothermal animals in different latitudes.—I. Proc. Zool. Soc. London 106:945–955.
Franz, D. R. 1971. Development and metamorphosis of the gastropod *Acteocina canaliculata* (Say). Trans. Am. Microsc. Soc. 90:174–182.
Franzén, Å. 1983. Ultrastructural studies of spermatozoa in three bivalve species with notes on evolution of elongated sperm nucleus in primitive spermatozoa. Gamete Res. 7:199–214.
Fraser, K. P. P., L. S. Peck, and A. Clarke. 2004. Protein synthesis, RNA concentrations, nitrogen excretion, and metabolism vary seasonally in the antarctic holothurian *Heterocucumis steineni* (Ludwig 1898). Physiol. Biochem. Zool. 77:556–569.
Freeman, K. A. 2001. Aquaculture and related biological attributes of abalone species in Australia – a review. Fish. Res. Rep. 128:1–48.
Fretter, V. 1951. Observation on the life history and functional morphology of *Cerithiopsis tubercularis* (Montagu) and *Triphora perversa* (L.). J. Mar. Biol. Assoc. United Kingdom 29:567–586. Monash University.
Fretter, V. 1948. The structure and life history of some minute prosobranchs of rock pools: *Skeneopsis planorbis* (Fabricius), *Omalogyra atomus* (Philippi), *Rissoella diaphana* (Alder) and *Rissoella opalina* (Jeffreys). J. Mar. Biol. Assoc. United Kingdom 27:597–632. Monash University.
Fretter, V., and M. C. Pilkington. 1970. Prosobranchia: Veliger larvae of Taenioglossa and Stenoglossa. ICES Identif. Leafl. Plankt. 2–26.
Fuchs, J., and A. Altenburger. 2017. Brachiopoda. Pp. 573–576 *in* C. Castellani and M. Edwards, eds. Marine Plankton: A practical guide to ecology, methodology, and taxonomy. Oxford University Press, Oxford, U.K.
Fuji, A., and M. Hashizume. 1974. Energy budget for a Japanese common scallop, *Patinopecten yessoensis* (Jay), in Mutsu Bay. Bull. Fac. Fish. Sci. Hokkaido Univ. 25:7–19.
Gage, J. D., R. M. Anderson, P. A. Tyler, R. Chapman, and E. Dolan. 2004. Growth, reproduction and possible recruitment variability in the abyssal brittle star *Ophiocten hastatum* (Ophiuroidea: Echinodermata) in the NE Atlantic. Deep. Res. Part I Oceanogr. Res. Pap. 51:849–864.
Gage, J. D., and P. A. Tyler. 1982. Growth and reproduction of the deep-sea brittlestar

Ophiomusium lymani. Oceanol. Acta 5:73–84.
Galasso, H. L., M. Richard, S. Lefebvre, C. Aliaume, and M. D. Callier. 2018. Body size and temperature effects on standard metabolic rate for determining metabolic scope for activity of the polychaete *Hediste (Nereis) diversicolor*. PeerJ 6:e5675.
Gallager, S. M., R. D. Turner, and C. J. Berg. 1981. Physiological aspects of wood consumption, growth, and reproduction in the shipworm <i>Lyrodus pedicellatus<i> Quatrefages (Bivalvia:Teredinidae). J. Exp. Mar. Bio. Ecol. 52:63–77.
Gallardo, C. S. 1979. Developmental pattern and adaptations for reproduction in *Nucella crassilabrum* and other muricacean gastropods. Biol. Bull. 157:453–463.
Gallardo, C. S. 1977. Two modes of development in the morphospecies *Crepidula dilatata* (Gastropoda: Calyptraeidae) from Southern Chile. Mar. Biol. 39:241–251.
Gallucci, V. F., and B. B. Gallucci. 1982. Reproduction and ecology of the hermaphroditic cockle *Clinocardium nuttallii* (Bivalvia: Cardiidae) in Garrison Bay. Mar. Ecol. Prog. Ser. 7:137–145.
Galtsoff, P. S., and V. L. Loosanoff. 1939. Natural history and method of controlling the starfish (*Asterias forbesi*, Desor). Bull. Bur. Fish. 49:75–132.
Gambi, M. C., and F. P. Patti. 1999. Reproductive biology of *Perkinsiana antarctica* (Kinberg) (Polychaeta, Sabellidae) in the Straits of Magellan (south America): Systematic and ecological implications. Sci. Mar. 63:253–259.
Gambi, M., F. Patti, G. Micaletto, and A. Giangrande. 2001. Diversity of reproductive features in some Antarctic polynoid and sabellid polychaetes, with a description of *Demonax polarsterni* sp. n. (Polychaeta, Sabellidae). Polar Biol. 24:883–891.
Garcia-Cisneros, A., C. Palacín, Y. Ben Khadra, and R. Pérez-Portela. 2016. Low genetic diversity and recent demographic expansion in the red starfish *Echinaster sepositus* (Retzius 1816). Sci. Rep. 6:1–16. Nature Publishing Group.
Garrido, O., and C. S. Gallardo. 1996. Ultrastructure of sperms in bivalve molluscs of the mytilidae family. Invertebr. Reprod. Dev. 29:95–102.
Gascoigne, T., and J. B. Sigurdsson. 1977. *Calliopaea oophaga* Lemche, 1974, a species new to the British fauna (Opisthobranchia: Sacoglossa). J. Molluscan Stud. 43:286–289.
Gasmi, H., O. Maamcha, T. Daas, and P. Scaps. 2016. First record of *Perinereis macropus* and *Perinereis cultrifera* (Annelida, Polychaeta) from rocky shores east Algeria, (SW Mediterranean sea). J. Entomol. Zool. Stud. 4:160–166.
Gemmill, J. F. 1920a. IX.—The development of the sea-anemones *Metridium dianthus* (Ellis) and *Adamsia palliata* (Bohad). Philos. Trans. R. Soc. London. Ser. B, Contain. Pap. a Biol. Character 209:351–375.
Gemmill, J. F. 1914. The development and certain points in the adult structure of the starfish *Asterias rubens* L. Philos. Trans. R. Soc. London. Ser. B, Contain. Pap. a Biol. Character 205:213–294.
Gemmill, J. F. 1920b. The development of the starfish *Crossaster papposus*. Q. J. Microsc. Sci. 64:155–187.
Gentil, F., J. C. Dauvin, and F. Ménard. 1990. Reproductive biology of the polychaete *Owenia fusiformis* Delle Chiaje in the Bay of Seine (eastern English Channel). J. Exp. Mar. Bio. Ecol. 142:13–23.
George, J. D. 1966. Reproduction and early development of the spionid polychaete *Scolecolepides viridis* (Verrill). Biol. Bull. 130:76–93.
George, S. B. 1994a. Population differences in maternal size and offspring quality for *Leptasterias epichlora* (Brandt) (Echinodermata: Asteroidea). J. Exp. Mar. Bio. Ecol. 175:121–131.
George, S. B. 1994b. The *Leptasterias* (Echinodermata, Asteroidea) species complex - Variation in reproductive investment. Mar. Ecol. Prog. Ser. 109:95–98.

Gerdes, D. 1983. The Pacific oyster *Crassostrea gigas*. Part II. Oxygen consumption of larvae and adults. Aquaculture 31:221–231.

Ghedini, G., C. R. White, and D. J. Marshall. 2018. Metabolic scaling across succession: Do individual rates predict community-level energy use? Funct. Ecol. 32:1447–1456.

Ghiselin, M. T. 1969. The evolution of hermaphroditism among animals.

Giangrande, A., and G. Cantone. 1990. Redescription and systematic position of *Pseudofabricia aberrans* Cantone, 1972 (Polychaeta, Sabellidae, Fabriciinae). Bolletino di Zool. 57:361–364.

Giangrande, A., and M. Licciano. 2008. Revision of the species of *Megalomma* (Polychaeta, Sabellidae) from the Mediterranean Sea, with the description of *M. messapicum* n. sp. Ital. J. Zool. 75:207–217.

Giangrande, A., M. Montresor, A. Cavallo, and M. Licciano. 2002a. Influence of *Naineris laevigata* (Polychaeta: Orbiniidae) on vertical grain size distribution, and dinoflagellate resting stages in the sediment. J. Sea Res. 47:97–108.

Giangrande, A., and A. Petraroli. 1991. Reproduction, larval development and post-larval growth of *Naineris laevigata* (Polychaeta, Orbiniidae) in the Mediterranean Sea. Mar. Biol. 111:129–137.

Giangrande, A., S. Quarta, and C. Caroppo. 1993. Observations of *Spio decoratus* (Polychaeta Spionidae) life history under laboratory conditions with taxonomic considerations. Oebalia 18:83–93.

Giangrande, A., M. Sciscioli, E. Lepore, M. Mastrodonato, P. Lupetti, and R. Dallai. 2002b. Sperm ultrastructure and spermiogenesis in two *Exogone species* (Polychaeta, Syllidae, Exogoninae). Invertebr. Biol. 121:339–349.

Gibbs, P. E. 1971. A comparative study of reproductive cycles in four polychaete species belonging to the family Cirratulidae. J. Mar. Biol. Assoc. United Kingdom 51:745–769.

Gibson, G. D. 2003. Larval development and metamorphosis in *Pleurobranchaea maculata*, with a review of development in the Notaspidea (Opisthobranchia). Biol. Bull. 205:121–132.

Gibson, G. D., and F.-S. Chia. 1989. Description of a new species of *Haminoea*, *Haminoea callidegenita* (Mollusca: Opisthobranchia), with a comparison with two other *Haminoea* species found in the northeast Pacific. Can. J. Zool. 67:914–922.

Gibson, P. H. 1977. Reproduction in the cirratulid polychaetes *Dodecaceria concharum* and *D. pulchra*. J. Zool. 182:89–102.

Giere, O., B. Ebbe, and C. Erséus. 2008. *Questa* (Annelida, Polychaeta, Orbiniidae) from Pacific regions - new species and reassessment of the genus Periquesta. Org. Divers. Evol. 7:304–319.

Giere, O., and C. Erséus. 1998. A systematic account of the Questidae (Annelida, Polychaeta), with description of new taxa. Zool. Scr. 27:345–360.

Giere, O. W., and N. W. Riser. 1981. Questidae—Polychaetes with oligochaetoid morphology and development. Zool. Scr. 10:95–103.

Giguere, M., G. Cliche, and S. Brulotte. 1994. Reproductive cycles of the sea scallop, *Placopecten magellanicus* (Gmelin), and the Iceland scallop, Chlamys islandica (O.F. Muller), in Iles-de-la-Madeleine, Canada. J. Shellfish Res. 13:31–36.

Gil, D. G., G. Escudero, and H. E. Zaixso. 2011. Brooding and development of *Anasterias minuta* (Asteroidea: Forcipulata) in Patagonia, Argentina. Mar. Biol. 158:2589–2602.

Glasby, C. J. 1986. Population structure and reproductive biology of *Ceratonereis limnetica* (Polychaeta: Nereididae) at Lower Portland, Hawkesbury River, Australia. Mar. Biol. 90:589–595.

Glon, H., Y. Haruka, M. Daly, and M. Nakaoka. 2019. Temperature and salinity survival limits of the fluffy sea anemone, *Metridium senile* (L.), in Japan. Hydrobiologia

830:303–315. Springer International Publishing.
Goddard, J. 1983. The opisthobranchs of Cape Arago, Oregon, with notes on their biology and a summary of benthic opisthobranchs known from Oregon. University of Oregon.
Goddard, J. 1984. The opisthobranchs of Cape Arago, Oregon, with notes on their biology and a summary of benthic opisthobranchs known from Oregon. The Veliger 27:143–163.
Goddard, J. H. R. 2004. Developmental mode in benthic opisthobranch molluscs from the northeast Pacific Ocean: Feeding in a sea of plenty. Can. J. Zool. 82:1954–1968.
Goddard, J. H. R. 1992. Patterns of development in nudibranch molluscs from the Northeast Pacific ocean, with regional comparisons. University of Oregon.
Goddard, J. H. R. 1991. Unusually large polar bodies in an aeolid nudibranch: A novel mechanism for producing extra-embryonic yolk reserves. J. Molluscan Stud. 57:143–152.
Gohar, H. A. F., and G. N. Soliman. 1967. The biology and development of *Asteronotus cespitosus* (Van Hasselt) (Gastropoda, Nudibranchia). Publ. Mar. Biol. Stn. Al Ghardaqa (Red Sea) 14:177–195.
González-Araya, R., and R. Robert. 2018. Larval development and fatty acid composition of *Ostrea edulis* (L.) fed four different single diets from conditioning to pre-settlement. Aquac. Res. 49:1768–1781.
Gonzalez-Bernat, M. J., M. Lamare, S. Uthicke, and M. Byrne. 2013. Fertilisation, embryogenesis and larval development in the tropical intertidal sand dollar *Arachnoides placenta* in response to reduced seawater pH. Mar. Biol. 160:1927–1941.
Gonzalez, H., and I. Huerta. 1995. Consumo de oxígeno de *Concholepas concholepas* (Bruguiere, 1789) en relación a la temperatura y talla en sistema controlado.
Goodheart, J. A., R. A. Ellingson, X. G. Vital, H. C. G. Filho, J. B. McCarthy, S. M. Medrano, V. J. Bhave, K. García-Méndez, L. M. Jiménez, G. López, C. A. Hoover, J. D. Awbrey, J. M. De Jesus, W. Gowacki, P. J. Krug, and Á. Valdés. 2016. Identification guide to the heterobranch sea slugs (Mollusca: Gastropoda) from Bocas del Toro, Panama. Mar. Biodivers. Rec. 9:1–31. Marine Biodiversity Records.
Görlitz, S. 2011. The lugworm *Abarenicola affinis* (Arenicolidae, Polychaeta) in tidal flats of Otago, southern New Zealand. University of Otago.
Gosliner, T. M. 1995. Introduction and spread of *Philine auriformis* (Gastropoda: Opisthobranchia) from New Zealand to San Francisco Bay and Bodega Harbor. Mar. Biol. 122:249–255.
Gosliner, T. M. 1987. Nudibranchs of Southern Arfrica: A guide to opistobranch molluscs of Southern Africa.
Gotshall, D. W. 2005. Guide to marine invertebrates: Alaska to Baja California. 2nd ed. Sea Challengers.
Götze, E. 1938. Bau und Leben von *Caecum glabrum* (Montagu). Zool. Jahrbücher, Syst. 71:55–122.
Gouveneaux, A. 2016. Bioluminescence of Tomopteridae species (Annelida): multidisciplinary approach. Université catholique de Louvain.
Grahame, J. 1994. Energetics of growth and reproduction in two species of chink shells (*Lacuna*, Mollusca: Prosobranchia). Cah.Biol.Mar. 35:327–338.
Grahame, J. 1982. Energy flow and breeding in two species of *Lacuna*: Comparative costs of egg production and maintenance. Int. J. Invertebr. Reprod. 5:91–99.
Grahame, J. 1969. The biology of *Berthelinia caribbea* Edmunds. Bull. Mar. Sci. 19:868–879.
Grainger, E. H. 1964. Asteroidea of the Blue Dolphin Expeditions to Labrador. Proc. United States Natl. Museum 115:31–46.

Grange, L. J. 2005. Reproductive success in Antarctic marine invertebrates.
Grant, J., and P. J. Cranford. 1991. Carbon and nitrogen scope for growth as a function of diet in the sea scallop *Placopecten magellanicus*. J. Mar. Biol. Assoc. United Kingdom 71:437–450. Monash University.
Grassle, J. P., and J. F. Grassle. 1976. Sibling species in the marine pollution indicator *Capitella* (Polychaeta). Science. 192:567–569.
Greenwood, P. J. 1980. Growth, respiration and tentative energy budgets for two populations of the sea urchin Parechinus angulosus (Leske). Estuar. Coast. Mar. Sci. 10:347–367.
Greer, D. L. 1962. Studies on the embryology of *Pycnopodia helianthoides* (Brandt) Stimpson. Pacific Sci. 16:280–285.
Grehan, A., C. Retière, and B. Keegan. 1991. Larval development in the ampharetid *Melinna palmata* Grube (Polychaeta). Ophelia Suppl. 5:321–332.
Grémare, A., and J. Amouroux. 1988. Experimental study ingestion and metabolism rates of *Eupolymnia nebulosa*: influence of state of sexual maturation. Oceanol. Acta 11:299–305.
Griffiths, C. L., and J. A. King. 1979. Some relationships between size, food availability and energy balance in the ribbed mussel Aulacomya ater. Mar. Biol. 51:141–149.
Haaland, B., and T. A. Schram. 1982. Larval development and metamorphosis of *Gyptis rosea* (Malm) (Hesionidae, Polyghaeta). Sarsia 67:107–118.
Haaland, B., and T. A. Schram. 1983. Larval development and metamorphosis of *Ophiodromus flexuosus* (Delle Chiaje) (Hesionidae, Polychaeta). Sarsia 68:85–90.
Hadfield, M. G. 1979. Aplacophora. Reprod. Mar. Invertebr. V:1–25.
Hain, S., and P. M. Arnaud. 1992. Notes on the reproduction of high-Antarctic molluscs from the Weddell Sea. Polar Biol. 12:303–312.
Hamatani, I. 1960. Notes on veligers of Japanese opisthobranchs. Publ. Seto Mar. Biol. Lab. 8:307–315.
Hamatani, I. 1967. Notes on veligers of Japanese opisthobranchs (7). Publ. Seto Mar. Biol. Lab. 15:121–131.
Hamburger, K., F. Mohlenberg, A. Randlov, and H. U. Riisg. 1983. Size, oxygen consumption and growth in the mussel *Mytilus edulis*. Mar. Biol. 306:303–306.
Hamel, J. F., and J. H. Himmelman. 1992. Sexual dimorphism in the sand dollar *Echinarachnius parma*. Mar. Biol. 113:379–383.
Hannerz, L. 1956. Larval development of the polychaete families Spionidae Sars, Disomidae Mesnil, and Poecilochaetidae n. fam. in the Gullmar Fjord (Sweden). Zool. Bidr. fran Uppsala 31:1–204.
Hansen, J. P., D. Robertson-Andersson, and M. Troell. 2006. Control of the herbivorous gastropod *Fissurella mutabilis* (Sow.) in a land-based integrated abalone-seaweed culture. Aquaculture 255:384–388.
Harbo, R. M. 1997. Shells and shellfish of the Pacific Northwest - A field guide. Harbour Publishing.
Hardege, J. D., and H. D. Bartels-Hardege. 1995. Spawning behaviour and development of *Perinereis nuntia* var. *brevicirrus* (Annelida: Polychaeta). Invertebr. Biol. 114:39.
Hargrave, B. T., V. E. Kostylev, and C. M. Hawkins. 2004. Benthic epifauna assemblages, biomass and respiration in The Gully region on the Scotian Shelf, NW Atlantic Ocean. Mar. Ecol. Prog. Ser. 270:55–70.
Harper, E. M., and L. Peck. 2003. Predatory behaviour and metabolic costs in the Antarctic muricid gastropod *Trophon longstaffi*. Polar Biol. 26:208–217.
Harrigan, J. F., and D. L. Alkon. 1978. Larval rearing, metamorphosis, growth and reproduction of the eolid nudibranch *Hermissenda crassicornis* (Eschscholtz, 1831)(Gastropoda: Opisthobranchia). Biol. Bull. 154:430–439.

Harriott, V. J. 1985. Reproductive biology of three congeneric sea cucumber species, *Holothuria atra*, *H. impatiens* and *H. edulis*, at Heron Reef, Great Barrier Reef. Mar. Freshw. Res. 36:51–57.
Harris, L. G. 1975. Studies on the life history of two coral-eating nudibranchs of the genus *Phestilla*. Biol. Bull. 149:539–550.
Harris, L. G., M. Powers, and J. Ryan. 1980. Life history studies of the estuarine nudibranch *Tenellia fuscata* (Gould, 1870). Veliger 23:70–74.
Hartman, O. 1947. Allan Hancock Pacific expeditions: Polychaetous Annelids Parts V-VIII. Los Angeles, CA, USA.
Hartman, O. 1966. Polychaeta Myzostomidae and Sedentaria of Antarctica. 7th ed. American Geophysical Union, Baltimore, MD USA.
Hartman, O. 1944. Polychaetous annelids from California: Including the descriptions of two new genera and nine new species (Plates 19-26).
Havenhand, J. N. 1993. Egg to juvenile period, generation time, and the evolution of larval type in marine invertebrates. Mar. Ecol. Prog. Ser. 97:247–260.
Haycock, L. J. 2004. The reproduction and recruitment of the sand dollar *Arachnoides placenta* (L.)(Echinoidea: Echinodermata) from differing habitats on the North Queensland coast. James Cook University.
Hayward, P. J., and J. S. Ryland. 2017. Annelids. Pp. 583–605 *in* P. J. Hayward and J. S. Ryland, eds. Handbook of the Marine Fauna of North-West Europe. Oxford University Press.
Heacox, A. 1980. Reproduction and larval development of *Typosyllis pulchra* (Berkeley & Berkeley) (Polychaeta: Syllidae). Pacific Sci. 34:245–259.
Healy, J. M., K. P. Beames, and D. B. Barclay. 1998. Spermatozoa of the Australian ‘greenlip’abalone *Haliotis laevigata* Donovan: ultrastructure and comparison with other gastropods, especially other Haliotidae (Vetigastropoda, Molluscs). Invertebr. Reprod. Dev. 34:197–206.
Heath, H. 1917. The early development of a starfish, *Pateria* (*Asterina*) *mineata*. J. Morphol. 29:461–469.
Heffernan, P., and B. F. Keegan. 1988. The larval development of *Pholoe minuta* (Polychaeta: Sigalionidae) in Galway Bay, Ireland. J. Mar. Biol. Assoc. United Kingdom 68:339–350.
Heilmayer, O., C. Bremec, T. Brey, and M. Lasta. 2001. Why are there no Patagonic scallops (*Zygochlamys patagonica*) North of the Rio Plata Estuary? P. 103 *in* J. E. Illanes, ed. 2001 Scallop Odyssey: 13th International Pectinid Workshop. Universidad Catolica Delnorte, Coquimbo, Chile.
Heilmayer, O., and T. Brey. 2003. Saving by freezing? Metabolic rates of *Adamussium colbecki* in a latitudinal context. Mar. Biol. 143:477–484.
Heller, J. 2015. Sea Snails. Springer International Publishing, Switzerland.
Henderson, J. A., and J. S. Lucas. 1971. Larval development and metamorphosis of *Acanthaster planci* (Asteroidea). Nature 232:655–657.
Hendler, G. 1975. Adaptational significance of the patterns of ophiuroid development. Integr. Comp. Biol. 15:691–715.
Hendler, G. 1991a. Echinodermata: Ophiuroidea. Pp. 355–511 *in* A. C. Giese and J. S. Pearse, eds. Reproduction of Marine Invertebrates. Blackwell Scientific and The Boxwood Press, Pacific Grove, California.
Hendler, G. 1991b. Echinodermata: Ophiuroidea. Pp. 355–511 *in* A. C. Giese and J. S. Pearse, eds. Reproduction of Marine Invertebrates, Vol. VI. Blackwell Scientific and The Boxwood Press, Pacific Grove, California.
Hendler, G. 1979. Sex-reversal and viviparity in *Ophiolepis kieri*, n. sp. with notes on

viviparous brittlestars from the Caribbean (Echinodermata: Ophiuroidea). Proc. Biol. Soc. Washingt. 92:783–795.
Hendler, G., and M. Byrne. 1987. Fine structure of the dorsal arm plate of *Ophiocoma wendti*. 261–272.
Hendler, G., and D. R. Franz. 1982. The biology of a brooding seastar, *Leptasterias tenera*, in Block Island Sound. Biol. Bull. 162:273–289.
Hendler, G., and B. S. Littman. 1986. The ploys of sex: relationships among the mode of reproduction, body size and habitats of coral-reef brittlestars. Coral Reefs 5:31–42.
Hendler, G., and L. U. Tran. 2001. Reproductive biology of a deep-sea brittle star *Amphiura carchara* (Echinodermata: Ophiuroidea). Mar. Biol. 138:113–123.
Hermans, C. O. 1962. The method of swimming and release of gametes in the opheliid polychaete *Armandia brevis*. Am. Zool. 4:292.
Herold, R. C. 1969. Hermaphrodite specimen of the sand dollar, *Echinarachnius parma*. J. Fish. Res. Board Canada 26:1965–1966.
Herrando-Pérez, S., G. San Martín, and J. Núñez. 2001. Polychaete patterns from an oceanic island in the eastern Central Atlantic: La Gomera (Canary Archipelago). Cah. Biol. Mar. 42:275–287.
Herrera, N. D. 2013. Molecular phylogenetics and historical biogeography of cockles and giant clams (Bivalvia: Cardiidae). Florida State University.
Hess, H. C. 1993. The evolution of parental care in brooding spirorbid polychaetes: The effect of scaling constraints. Am. Nat. 141:577–596.
Hesse, R., and F. Doflein. 1914. *Tierbau und Tierleben*. Teubner Leipzig.
Hiebert, T. C. 2015a. *Armandia brevis*. P. *in* T. C. Hiebert, B. A. Butler, and A. L. Shanks, eds. Oregon Estuarine Invertebrates: Rudys' Illustrated Guide to Common Species. University of Oregon Libraries and Oregon Institute of Marine Biology, Charleston, OR.
Hiebert, T. C. 2015b. *Halosydna brevisetosa*. P. *in* T. C. Hiebert, B. A. Butler, and A. L. Shanks, eds. Oregon Estuarine Invertebrates: Rudys' Illustrated Guide to Common Species. University of Oregon Libraries and Oregon Institute of Marine Biology, Charleston, OR.
Hiebert, T. C. 2015c. *Leitoscoloplos pugettensis*. P. *in* T. C. Hiebert, B. A. Butler, and A. L. Shanks, eds. Oregon Estuarine Invertebrates: Rudys' Illustrated Guide to Common Species. University of Oregon Libraries and Oregon Institute of Marine Biology, Charleston, OR.
Hiebert, T. C. 2015d. *Magelona sacculata*. P. *in* T. C. Hiebert, B. A. Butler, and A. L. Shanks, eds. Oregon Estuarine Invertebrates: Rudys' Illustrated Guide to Common Species. University of Oregon Libraries and Oregon Institute of Marine Biology, Charleston, OR.
Highnam, K. C. 1977. Control of Ovarian Development in Invertebrates. Pp. 1–62 *in* Lord Zuckerman and B. J. Weir, eds. The Ovary. Academic Press Inc.
Hilário, A., C. M. Young, and P. A. Tyler. 2005. Sperm storage, internal fertilization, and embryonic dispersal in vent and seep tubeworms (Polychaeta: Siboglinidae: Vestimentifera). Biol. Bull. 208:20–28.
Hiratsuka, Y., and T. Uehara. 2007. Feeding rates and absorption efficiencies of four species of sea urchins (genus *Echinometra*) fed a prepared diet. Comp. Biochem. Physiol. - A Mol. Integr. Physiol. 148:223–229.
Hirche, H. J. 1984. Temperature and metabolism of plankton-I. Respiration of antarctic zooplankton at different temperatures with a comparison of antarctic and nordic krill. Comp. Biochem. Physiol. -- Part A Physiol. 77:361–368.
Hoagland, K. E. 1983. Ecology and larval development of *Crepidula protea* (Prosobranchia: Crepidulidae) from southern Brasil: a new type of egg capsule for the genus. Nautilus

(Philadelphia). 97:105–109.
Hoagland, K. E. 1986. Patterns of encapsulation and brooding in the Calyptraeidae (Prosobranchia, Mesogastropoda). Am. Malacol. Bull. 4:173–183.
Hoagland, K. E., and W. R. Coe. 1982. Larval development in *Crepidula maculosa* (Prosobranchia: Crepidulidae) from Florida. Nautilus (Philadelphia). 96:122.
Hobson, K. D. 1967. The feeding and ecology of two north Pacific *Abarenicola* species (Arenicolidae, Polychaeta). Biol. Bull. 133:343–354.
Hoggett, W. A. K. 1991. The genus *Macrophiothrix* (Ophiuroidea: Ophiotrichidae) in Australian waters. Invertebr. Syst. 4:1046–1077.
Holland, N. D. 1967. Gametogenesis during the annual reproductive cycle in a cidaroid sea urchin (*Stylocidaris affinis*). Biol. Bull. 133:578–590.
Hopcroft, R. R., D. B. Ward, and J. C. Roff. 1985. The relative significance of body surface and cloacal respiration in *Psolus fabricii* (Holothuroidea: Dendrochirotida). Can. J. Zool. 63:2878–2881.
Horn, P. L. 1985. Respiration in air and water of the chiton *Chiton pelliserpentis* from high and low zones of a sheltered shore. New Zeal. J. Mar. Freshw. Res. 19:11–19.
Horsford, I. A. N., H. Simon, M. Ishida, M. Archibald, J. Webber, R. O. Y. Morris, and A. Desouza. 2013. The morphology of the queen conch (*Strombus gigas*) from the Island of Barbuda – Implications for fisheries management. Pp. 450–457 *in* Proceedings of the 65th Gulf and Caribbean Fisheries Institute.
Hörstadius, S. 1939. Über die entwicklung von *Astropecten aranciacus* L. Pubbl. della Stn. Zool. di Napoli 17:221–312.
Houbrick, J. 1971. Some aspects of the anatomy, reproduction, and early development of *Cerithium nodulosum* (Bruguiere) (Gastropoda, Prosobranchia). Pacific Sci. 25:560–565.
Houbrick, R. S. 1973. Studies of the reproductive biology of the genus *Cerithium* (Gastropoda: Prosobranchia) In the Western Atlantic. Bull. Mar. Sci. 23:875–904.
Houlihan, D. F. 1979. Respiration in air and water of three mangrove snails. J. Exp. Mar. Bio. Ecol. 41:143–161.
Hourdez, S., R. E. Weber, B. N. Green, J. M. Kenney, and C. R. Fisher. 2002. Respiratory adaptations in a deep-sea orbiniid polychaete from Gulf of Mexico brine pool NR-1: Metabolic rates and hemoglobin structure/function relationships. J. Exp. Biol. 205:1669–1681.
Hsieh, H.-L., and J. L. Simon. 1987. Larval development of *Kinbergonuphis simoni*, with a summary of development patterns in the family Onuphidae (Polychaeta). Bull. Biol. Soc. Wash. 7:194–210.
Hsieh, H. L. 1995. *Laonome albicingillum*, a new fan worm species (Polychaeta, Sabellidae, Sabellinae) from Taiwan. Proc. Biol. Soc. Washingt. 108:130–135.
Hsieh, H. L., and J. L. Simon. 1991. Life history and population dynamics of *Kinbergonuphis simoni* (Polychaeta: Onuphidae). Mar. Biol. 110:117–125.
Huang, D., K. Fitzhugh, and G. W. Rouse. 2011. Inference of phylogenetic relationships within Fabriciidae (Sabellida, Annelida) using molecular and morphological data. Cladistics 27:356–379.
Huchette, S. M. H., J. P. Soulard, C. S. Koh, and R. W. Day. 2004. Maternal variability in the blacklip abalone, *Haliotis rubra* Leach (Mollusca: Gastropoda): effect of egg size on fertilisation success. Aquaculture 231:181–195.
Hudson, M. E., A. Turner, and M. A. Sewell. 2015. Comparative ultrastructure of spermatozoa from two regular and two irregular New Zealand echinoids. Invertebr. Biol. 134:341–351.
Hughes, D. J., E. S. Poloczanska, and J. Dodd. 2008. Survivorship and tube growth of reef-

building *Serpula vermicularis* (Polychaeta: Serpulidae) in two Scottish sea lochs. Aquat. Conserv. Mar. Freshw. Ecosyst. 18:117–129.
Hughes, R. N. 1970. An energy budget for a tidal-flat population of the bivalve *Scrobicularia plana* (Da Costa). J. Anim. Ecol. 39:357.
Hughes, R. N. 1971. Ecological energetics of *Nerita* (Archaeogastropoda, Neritacea) populations on Barbados, West Indies. Mar. Biol. 11:12–22.
Hughes, R. N., and H. P. I. Hughes. 1987. Spawning and hatching of Cypraecassis testiculus Linnaeus, 1758 (Tonnacea: Cassidae). The Veliger 29:256–259.
Hughes, S. J. M., H. A. Ruhl, L. E. Hawkins, C. Hauton, B. Boorman, and D. S. M. Billett. 2011. Deep-sea echinoderm oxygen consumption rates and an interclass comparison of metabolic rates in Asteroidea, Crinoidea, Echinoidea, Holothuroidea and Ophiuroidea. J. Exp. Biol. 214:2512–2521.
Huo, D., L. Sun, X. Ru, L. Zhang, C. Lin, S. Liu, X. Xin, and H. Yang. 2018. Impact of hypoxia stress on the physiological responses of sea cucumber *Apostichopus japonicus*: Respiration, digestion, immunity and oxidative damage. PeerJ 2018.
Hutchings, P. H. 1973. Age structure and spawning of a Northumberland population of *Melinna cristata* (Polychaeta: Ampharetidae). Mar. Biol. 18:218–227.
Hyman, L. H. 1955. Echinodermata. McGraw-Hill, New York.
Ichikawa, M. 1993. Saccoglossa (Opisthobranchia) from the Ryukyu Islands. Publ. Seto Mar. Biol. Lab. 36:119–139.
Idrisi, N., T. R. Capo, and J. E. Serafy. 2003. Postmetamorphic growth and metabolism of long-spined black sea urchin (Diadema antillarum) reared in the laboratory. Mar. Freshw. Behav. Physiol. 36:87–95.
Ikeda, T., and E. H. Fay. 1981. Metabolic activity of zooplankton from the Antarctic Ocean. Mar. Freshw. Res. 32:921–930.
Ikeda, T., and A. W. Mitchell. 1982. Oxygen uptake, ammonia excretion and phosphate excretion by krill and other Antarctic zooplankton in relation to their body size and chemical composition. Mar. Biol. 71:283–298.
Ikeda, T., and H. R. Skjoldal. 1989. Metabolism and elemental composition of zooplankton from the Barents Sea during early Arctic summer. Mar. Biol. 100:173–183.
Inglefield, E. A. 2014. A summer search for Sir John Franklin: with a peep into the polar basin. Cambridge University Press.
Ishiki, H. 1938. Histological studies on the sexual organs during sex-changes of *Crepidula aculeata* and *C. walshi*. J. Sci. Hiroshima Univ. 103–113.
Ituarte, C. 2009. Unusual modes of oogenesis and brooding in bivalves: The case of *Gaimardia trapesina* (Mollusca: Gaimardiidae). Invertebr. Biol. 128:243–251.
Ivleva, I. V. 1973. Quantitative correlation of temperature and respiration rate in poikilothermic animals. Pol. Achiwum Hydrobiol. 20:283–300.
Iwata, F., and M. Yamashita. 1982. Annual reproductive cycle of the brittle-star *Amphipholis kochii* (Echinodermata: Ophiuroidea), with special reference to the growth pattern of oocytes. Publ. Seto Mar. Biol. Lab. 27:143–153.
Jägersten, G. 1952. Studies on the morphology, larval development and biology of *Protodrilus*. Zool. Bidr. fran Uppsala 29:426–511.
Jakovcev-Todorovic, D., V. Djikanovic, S. Milosevic, and P. Cakic. 2006. Discovery of polychaete species Manayunkia caspica (Annenkova, 1929) in the Serbian sector of the Danube. Arch. Biol. Sci. za Biol. Nauk. 58:35P-36P.
James, D. 1972. Note on the development of the asteroid *Asterina burtoni* Gray. J. Mar. Biol. Assoc. India 14:883–884.
James, M. A., A. D. Ansell, M. J. Collins, G. B. Curry, L. S. Peck, and M. C. Rhodes. 1992. Biology of living brachiopods. Adv. Mar. Biol. 28:175–387.

James, M. R., M. A. Weatherhead, and A. H. Ross. 2001. Size-specific clearance, excretion, and respiration rates, and phytoplankton selectivity for the mussel Perna canaliculus at low levels of natural food. New Zeal. J. Mar. Freshw. Res. 35:73–86.

Jamieson, B. G. M., and G. W. Rouse. 1989. The spermatozoa of the polychaeta (Annelida): an ultrastructural review.

Järnegren, J., and D. Altin. 2006. Filtration and respiration of the deep living bivalve Acesta excavata (J.C. Fabricius, 1779) (Bivalvia; Limidae). J. Exp. Mar. Bio. Ecol. 334:122–129.

Järnegren, J., H. T. Rapp, and C. M. Young. 2007. Similar reproductive cycles and life-history traits in congeneric limid bivalves with different modes of nutrition. Mar. Ecol. 28:183–192.

Jensen, K. 2001. Review of reproduction in the Sacoglossa (Mollusca, Opisthobranchia). Boll. Malacol. 5–8:81–98.

Jensen, K. R. 1985. Annotated checklist of Hong Kong Ascoglossa (Mollusca: Opisthobranchia), with descriptions of four new species. Pp. 77–107 *in* Proceedings of the Second International Workshop on the Malacofauna of Hong Kong and Southern China.

Jensen, K. R. 1986. Observations on feeding, copulation and spawning of the ascoglossan opisthobranch *Calliopaea oophaga* lemche. Ophelia 25:97–106.

Jensen, K. R. 1993. Sacoglossa (Mollusca, Opisthobranchia) from Rottnest Island and central Western Australia. Pp. 207–253 *in* Proceedings of the 5th International Marine Biological Workshop: The marine flora and fauna of Rottnest Island, Western Australia.

Jensen, K. R. 2015. Sacoglossa (Mollusca: Gastropoda: Heterobranchia) from northern coasts of Singapore. Raffles Bull. Zool. Suppl. 226–249.

Jensen, N., R. M. Allen, and D. J. Marshall. 2014. Adaptive maternal and paternal effects: Gamete plasticity in response to parental stress. Funct. Ecol. 28:724–733.

Joaquim, S., D. Matias, A. M. Matias, R. Gonçalves, L. Chícharo, and M. B. Gaspar. 2016. New species in aquaculture: Are the striped venus clam *Chamelea gallina* (Linnaeus, 1758) and the surf clam *Spisula solida* (Linnaeus 1758) potential candidates for diversification in shellfish aquaculture? Aquac. Res. 47:1327–1340.

John, C. C. 1933. Memoirs: Habits, structure, and development of *Spadella cephaloptera*. J. Cell Sci. s2-75:625–696.

Johnson, D. W., K. Monro, and D. J. Marshall. 2013. The maintenance of sperm variability: Context-dependent selection on sperm morphology in a broadcast spawning invertebrate. Evolution. 67:1383–1395.

Johnson, M. W. 1943. Studies on the life history of the marine annelid *Nereis vexillosa*. Biol. Bull. 84:106–114.

Johnson, S., and L. Boucher. 1983. Notes on some Opisthobranchia (Mollusca, Gastropoda) from the Marshall-Islands, including 57 new records. Pacific Sci. 37:251–291.

Johnson, W. S. 1973. Respiration rates of some New Zealand echinoderms (note). New Zeal. J. Mar. Freshw. Res. 7:165–169.

Jollivet, D., A. Empis, M. C. Baker, S. Hourdez, T. Comtet, C. Jouin-Toulmond, D. Desbruyères, and P. A. Tyler. 2000. Reproductive biology, sexual dimorphism, and population structure of the deep sea hydrothermal vent scale-worm, *Branchipolynoe seepensis* (Polychaeta: Polynoidae). J. Mar. Biol. Assoc. United Kingdom 80:55–68.

Jones, H. L., C. D. Todd, and W. J. Lambert. 1996. Intraspecific variation in embryonic and larval traits of the dorid nudibranch mollusc *Adalaria proxima* (Alder and Hancock) around the northern coasts of the British Isles. J. Exp. Mar. Bio. Ecol. 202:29–47.

Jonsson, P. R., K. M. Berntsson, C. André, and S. Å. Wängberg. 1999. Larval growth and settlement of the European oyster (*Ostrea edulis*) as a function of food quality measured

as fatty acid composition. Mar. Biol. 134:559–570.
Jørgensen, A., T. O. M. M. Boesgaard, N. Møbjerg, and R. M. Kristensen. 2014. The tardigrade fauna of Australian marine caves: With descriptions of nine new species of Arthrotardigrada. Zootaxa 3802:401–443.
Joyner, A. 1962. Reproduction and larval life of *Nerine cirratulus* (Dellee Chiaje) family Spionidae. Proc. Zool. Soc. London 138:655–666.
Just, E. E. 1922. On rearing sexually mature *Platynereis megalops* from eggs. Am. Nat. 56:471–478.
Kamel, S. J., F. X. Oyarzun, and R. K. Grosberg. 2010. Reproductive biology, family conflict, and size of offspring in marine invertebrates. Integr. Comp. Biol. 50:619–629.
Kang, D.-R., K. S. Tan, and L.-L. Liu. 2018. Egg-collar morphology and identity of nine species of Naticidae (Gastropoda) in Taiwan, with an assessment of their phylogenetic relationships. J. Molluscan Stud. 84:354–378.
Kang, D. H., H. S. Yang, H. S. Park, and K. S. Choi. 2007. Use of plate growth measurement for the estimation of skeletal growth of two sand dollars, *Astriclypeus manni* (Verrill 1867) and *Clypeaster japonicus* (Döderlein 1885), in Jeju, Korea. Plankt. Benthos Res. 2:77–82.
Kano, Y. T., and M. Komatsu. 1978. Development of the sea-star, *Asterina batheri* Goto. Dev. Growth Differ. 20:107–114.
Kano, Y. T., M. Komatsu, and C. Oguro. 1991. Morphological changes of the cushion star, *Culcita novaeguineae* Müller et Troschel, during growth. Pp. 323–326 *in* T. Yanagisawa, I. Yasumasu, C. Oguro, N. Suzuki, and T. Motokawa, eds. Biology of Echinodermata. Balkema, Rotterdam, Netherlands.
Kashenko, S. D. 2003. The reaction of the starfish *Asterias amurensis* and *Patina pectinifera* (Asteroidea) from Vostok Bay (Sea of Japan) to a salinity decrease. Russ. J. Mar. Biol. 29:110–114.
Kawaguti, S., and T. Yamasu. 1960. Spawning habits of a bivalved gastropod, *Tamanovalva limax*. Biol. J. Okayama Univ. 6:133–139.
Kaye, H. R., and H. M. Reiswig. 1991. Sexual reproduction in four caribbean commercial sponges. I. Reproductive cycles and spermatogenesis. Invertebr. Reprod. Dev. 19:1–11.
Keever, C. C. 2010. Life history and population genetic structure of sea stars from the family Asterinidae. Simon Fraser University.
Kelly, M. S., A. D. Hughes, and E. J. Cook. 2013. *Psammechinus miliaris*. Pp. 329–336 *in* J. M. Lawrence, ed. Sea Urchins: Biology and Ecology. Elsevier.
Kempf, S. C., and C. D. Todd. 1989. Feeding potential in the lecithotrophic larvae of *Adalaria proxima* and *Tritonia hombergi*: An evolutionary perspective. J. Mar. Biol. Assoc. United Kingdom 69:659–682.
Kempf, S. C., and A. O. D. Willows. 1977. Laboratory culture of the nudibranch *Tritonia diomedea* Bergh (Tritoniidae: Opisthobranchia) and some aspects of its behavioral development. J. Exp. Mar. Bio. Ecol. 30:261–276.
Kennedy, V., R. Lutz, and S. Fuller. 1989. Larval and early postlarval development of *Macoma mitchelli* Dall (Bivalvia: Tellinidae). Veliger 32:29–38.
Kennedy, V. S., and J. A. Mihursky. 1972. Effects of temperature on the respiratory metabolism of three Chesapeake Bay bivalves. Chesap. Sci. 13:1–22.
Kenny, R. 1969. Growth and asexual reproduction of the starfish *Nepanthia belcheri* (Perrier). Pacific Sci. 23:51–55.
Kerbl, A., E. W. Tolstrup, and K. Worsaae. 2019. Nerves innervating copulatory organs show common FMRFamide, FVRIamide, MIP and serotonin immunoreactivity patterns across Dinophilidae (Annelida) indicating their conserved role in copulatory behaviour. BMC Zool. 4:1–18. BMC Zoology.

Kersey Sturdivant, S., M. Perchik, R. W. Brill, and P. G. Bushnell. 2015. Metabolic responses of the Nereid polychaete, *Alitta succinea*, to hypoxia at two different temperatures. J. Exp. Mar. Bio. Ecol. 473:161–168. Elsevier B.V.

Kettle, B. T., and J. S. Lucas. 1987. Biometric relationships between organ indices, fecundity, oxygen consumption and body size in *Acanthaster planci*. Bull. Mar. Sci. 41:541–551.

Kikuchi, E. 1986. Contribution of the polychaete, *Neanthes japonica* (Izuka), to the oxygen uptake and carbon dioxide production of an intertidal mud-flat of the Nanakita River estuary, Japan. J. Exp. Mar. Bio. Ecol. 97:81–93.

Kincannon, E. A. 1975. The relations between body weight and habitat temperature and the respiratory rate of *Tonicella lineata* (Wood, 1815) (Mollusca: Polyplacophora). The Veliger 18:87–93.

Kinchington, P. R. G., and D. N. Nicholson. 1982. A description of spawning and post-gastrula development of the cool temperate coral, *Caryophyllia smithi*. J. Mar. Biol. Assoc. United Kingdom 62:845–854.

Klumpp, D. W., B. L. Bayne, and A. J. S. Hawkins. 1992. Nutrition of the giant clam *Tridacna gigas* (L). J. Exp. Mar. Bio. Ecol. 155:105–122.

Knight-Jones, P., and N. Bowden. 1984. Incubation and scissiparity in Sabellidae (Polychaeta). J. Mar. Biol. Assoc. United Kingdom 64:809–818.

Knudsen, J. 1950. Egg capsules and development of some marine prosobranchs from tropical West Africa. Danish Science Press.

Knudsen, J. 1994. Further observations on the egg capsules and reproduction of some marine prosobranch molluscs from Hong Kong. Hong Kong University Press, Hong Kong.

Koch, V. 1999. Epibenthic production and energy flow in the Caete mangrove estuary, North Brazil. Univ. Bremen, Ger. 91.

Kodama, M., J. G. Sumbing, M. J. H. Lebata-Ramos, and S. Watanabe. 2015. Metabolic rate characteristics and sediment cleaning potential of the tropical sea cucumber Holothuria scabra. Japan Agric. Res. Q. 49:79–84.

Kohn, A. J., and F. E. Perron. 1994. Life History and Biogeography: Patterns in *Conus*. Oxford University Press Inc., New York.

Kolbasova, G. D., A. B. Tzetlin, and E. K. Kupriyanova. 2013. Biology of *Pseudopotamilla reniformis* (Müller 1771) in the White Sea, with description of asexual reproduction. Invertebr. Reprod. Dev. 57:264–275.

Komatsu, M. 1983. Development of the sea-star, *Archaster typicus*, with a note on male-on-female superposition. Annot. Zool. Jpn. 56:187–195.

Komatsu, M. 1982. Development of the sea-star *Ctenopleura fisheri*. Mar. Biol. 66:199–205.

Komatsu, M. 1975. On the development of the sea star, *Astropecten latespinosus* Meissner. Biol. Bull. 148:49–59.

Komatsu, M., Y. T. Kano, and C. Oguro. 1990. Development of a true ovoviviparous sea star, *Asterina pseudoexigua pacifica* Hayashi. Biol. Bull. 179:254–263.

Komatsu, M., Y. T. Kano, H. Yoshizawa, S. Akabane, and C. Oguro. 1979. Reproduction and development of the hermaphroditic sea-star, *Asterina minor* Hayashi. Biol. Bull. 157:258–274.

Komatsu, M., M. Kawai, S. Nojima, and C. Oguro. 1994. Development of multiarmed seastar, *Luidia maculata* Müller and Troschel. P. *in* Echinoderms through Time. A A Balkema, Rotterdam.

Komatsu, M., and S. Nojima. 1985. Development of the seastar *Astropecten gisselbrechti* Döderlein. Pacific Sci. 39:274–282.

Komatsu, M., and C. Oguro. 1972. Notes on the hermaphroditic specimen of the sea-star, *Certonardoa semiregularis* (Muller et Troschel). Proc. Japanese Soc. Syst. Zool. 49–52.

Komatsu, M., C. Oguro, and Y. T. Kano. 1982. Development of the sea-star, *Luida quinaria*

von Martens. Pp. 497–503 *in* International Echinoderms Conference.
Komatsu, M., and S. Tomoko. 1993. Development of the brittle star, *Ophioplocus japonicus* H.L. Clark. I. Zoolog. Sci. 10:295–306.
Kremenetskaia, A., O. Ezhova, A. L. Drozdov, E. Rybakova, and A. Gebruk. 2020. On the reproduction of two deep-sea Arctic holothurians, *Elpidia heckeri* and *Kolga hyalina* (Holothuroidea:Elpidiidae). Invertebr. Reprod. Dev. 64:33–47. Taylor & Francis.
Kriegstein, A. R., V. Castellucci, and E. R. Kandel. 1974. Metamorphosis of *Aplysia californica* in laboratory culture. Proc. Natl. Acad. Sci. U. S. A. 71:3654–3658.
Kristensen, E. 1981. Direct measurement of ventilation and oxygen uptake in three species of tubicolous polychaetes (*Nereis* spp.). J. Comp. Physiol. 145:45–50.
Krug, P. J. 2009. Not My "Type": Larval dispersal dimorphisms and bet-hedging in opisthobranch life histories. Biol. Bull. 216:355–372.
Krug, P. J. 2007. Poecilogony and larval ecology in the gastropod genus Aladeria. Am. Malacol. Bull. 23:99–111.
Krug, P. J. 1998. Poecilogony in an estuarine opisthobranch: Planktotrophy, lecithotrophy, and mixed clutches in a population of the ascoglossan *Alderia modesta*. Mar. Biol. 132:483–494.
Krüger, F. 1970. Untersuchungen über die temperaturabhängigkeit des sauerstoffverbrauchs von *Crepidula fornicata* (Mollusca: Prosobranchia). Mar. Biol. 5:145–153.
Krüger, F. 1964. Versuche über die abhängigkeit der atmung von *Arenicola marina* (Annelides Polychaeta) von größe und temperatur. Helgoländer Wissenschaftliche Meeresuntersuchungen 10:38–63.
Kubo, K. 1951. Some observations on the development of the sea-star, *Leptasterias ochotensis similispinis* (Clark). J. Fac. Sci. Hokkaido Univ. 10:97–105.
Kumé, M., and K. Dan. 1968. Invertebrate embryology. Nolit Publishing House, Belgrade, Yugoslavia.
Kuper, M., and W. Westheide. 1997. Sperm ultrastructure and spermatogenesis in the interstitial polychaete *Sphaerosyllis hermaphrodita* (Syllidae: Exogoninae). Invertebr. Reprod. Dev. 32:189–200.
Kupriyanova, E. K. 2006. Fertilization success in *Galeolaria caespitosa* (Polychaeta: Serpillidae): gamete characteristics, role of sperm dilution, gamete age, and contact time. Pp. 309–317 *in* Scientia Marina.
Kupriyanova, E. K., E. Nishi, H. A. Ten Hove, and A. V Rzhavsky. 2001. Life-history patterns in serpulimorph polychaetes: Ecological and evolutionary perspectives. Pp. 1–101 *in* R. N. Gibson, M. Barnes, and R. J. A. Atkinson, eds. Oceanography and Marine Biology: an Annual Review. Taylor & Francis.
Kupriyanova, E. K., and A. V. Rzhavsky. 1993. *Serpula* and *Crucigera* (Polychaeta, Serpulidae) from the Russian far-eastern seas. Ophelia 38:47–54.
Kuzmina, T. V., E. N. Temereva, and V. V. Malakhov. 2016. Larval development of brachiopod *Coptothyris grayi* (Davidson, 1852) (Brachiopoda, Rhynchonelliformea). Dokl. Biol. Sci. 471:258–260.
LaBarbera, M. 1982. Metabolic rates of suspension feeding crinoids and ophiuroids (Echinodermata) in a unidirectional laminar flow. Comp. Biochem. Physiol. -- Part A Physiol. 71:303–307.
Lacalli, T. 1977. Remarks on the larvae of two serpulids (Polychaeta) from Barbados. Can. J. Zool. 55:300–303.
Lacalli, T. C. 1980. A guide to the marine flora and fauna of the Bay of Fundy: Polychaete larvae from Passamaquoddy Bay.
Laegdsgaard, P., M. Byrne, and D. T. Anderson. 1991. Reproduction of sympatric populations of *Heliocidaris erythrogramma* and *H. tuberculata* (Echinoidea) in New

South Wales. Mar. Biol. 110:359–374.
Lagos, N. A., F. J. Tapia, S. A. Navarrete, and J. C. Castilla. 2007. Spatial synchrony in the recruitment of intertidal invertebrates along the coast of central Chile. Mar. Ecol. Prog. Ser. 350:29–39.
Lalli, M., and F. E. Wells. 1978. Reproduction in the genus *Limacina* (Opisthobranchia: Thecosomata). J. Zool. 186:95–108.
Lau, S. C. Y., L. J. Grange, L. S. Peck, and A. J. Reed. 2018. The reproductive ecology of the Antarctic bivalve *Aequiyoldia eightsii* (Protobranchia: Sareptidae) follows neither Antarctic nor taxonomic patterns. Polar Biol. 41:1693–1706. Springer Berlin Heidelberg.
Lawrence, J. M., and J. Herrera. 2000. Stress and deviant reproduction in echinoderms. Zool. Stud. 39:151–171.
Lawrence, J. M., J. B. McClintock, and A. Guille. 1984. Organic level and caloric content of eggs of brooding asteroids and an echinoid (Echinodermata) from Kerguelen (South Indian Ocean). Int. J. Invertebr. Reprod. Dev. 7:249–257.
Lawson-Kerr, C., and D. T. Anderson. 1978. Reproduction, spawning and development of the starfish *Patiriella exigua* (Lamarck) (Asteroidea: Asterinidae) and some comparisons with *P. calcar* (Lamarck). Mar. Freshw. Res. 29:45–53.
Le Bourg, B. 1962. Trophic ecology of Southern Ocean seastars: Influence of environmental drivers on trophic diversity. University of Liège, Belgium.
Le Pennec, M., and P. G. Beninger. 2000. Reproductive characteristics and strategies of reducing-system bivalves. Comp. Biochem. Physiol. - A Mol. Integr. Physiol. 126:1–16.
Lebour, M. V. 1931. *Clione limacina* in Plymouth Waters. J. Mar. Biol. Assoc. United Kingdom 17:785–795.
Lebour, M. V. 1936. Notes on the eggs and larvae of some Plymouth prosobranchs. J. Mar. Biol. Assoc. United Kingdom 20:547–565.
Lebour, M. V. 1937. The eggs and larvae of the British prosobranchs with special reference to those living in the plankton. J. Mar. Biol. Assoc. United Kingdom 22:105–166.
Lebour, M. V. 1935. The larval stages of *Balcis alba* and *B. devians*. J. Mar. Biol. Assoc. United Kingdom 20:65–69.
Lebour, M. V. 1933. The life-histories of *Cerithiopsis tubercularis* (Montagu), *C. barleei* Jeffreys and *Triphora perversa* (L.). J. Mar. Biol. Assoc. United Kingdom 18:491–498.
Lebrato, M., D. Iglesias-Rodríguez, R. A. Feely, D. Greeley, D. O. B. Jones, N. Suarez-Bosche, R. S. Lampitt, J. E. Cartes, D. R. H. Green, and B. Alker. 2010. Global contribution of echinoderms to the marine carbon cycle: CaCO 3 budget and benthic compartments. Ecol. Monogr. 80:441–467.
Lehrke, J., and T. Bartolomaeus. 2009. Comparative morphology of spermatozoa in Echiura. Zool. Anz. 248:35–45.
Leonard, J. L. 2013. Williams' paradox and the role of phenotypic plasticity in sexual systems. Integr. Comp. Biol. 53:671–688.
Leone, D. E. 1970. The maturation of *Hydroides dianthus*. Biol. Bull. 138:306–315.
Leung, K. M. Y., A. C. Taylor, and R. W. Furness. 2000. Temperature-dependent physiological responses of the dogwhelk *Nucella lapillus* to cadmium exposure. J. Mar. Biol. Assoc. United Kingdom 80:647–660. Monash University.
Levitan, D. R., M. A. Sewell, and Fu-Shiang Chia. 1991. Kinetics of fertilization in the sea urchin *Strongylocentrotus franciscanus*: interaction of gamete dilution, age, and contact time. Biol. Bull. 181:371–378.
Lewis, L. S., J. E. Smith, and Y. Eynaud. 2018. Comparative metabolic ecology of tropical herbivorous echinoids on a coral reef. PLoS One 13:1–21.
Lezzi, M., F. Cardone, B. Mikac, and A. Giangrande. 2015. Variation and ontogenetic

changes of opercular paleae in a population of *Sabellaria spinulosa* (Polychaeta: Sabellaridae) from the South Adriatic Sea, with remarks on larval development. Sci. Mar. 79:137–150.

Li, B., Z. Zhou, B. Li, Q. Wang, X. Li, and L. Chen. 2018. Size distribution of individuals in the population of *Asterias amurensis* (Echinodermata: Asteroidea) and its reproductive cycle in China. Acta Oceanol. Sin. 37:96–103.

Licciano, M., A. Giangrande, and M. C. Gambi. 2002. Reproduction and simultaneous hermaphroditism in *Branchiomma luctuosum* (Polychaeta, Sabellidae) from the Mediterranean Sea. Invertebr. Biol. 121:55–65.

Lieberkind, I. 1920. On a star-fish (*Asterias groenlandica*) which hatches its young in its stomach. Vidensk. Medd. Dan. Naturhist. Foren. 72:121–126.

Linton, D. L., and G. L. Taghon. 2000. Feeding, growth, and fecundity of *Abarenicola pacifica* in relation to sediment organic concentration. J. Exp. Mar. Bio. Ecol. 254:85–107.

Liu, W., Y. Liao, and X. Li. 2006. *Luidia changi*, a new sea star species (Echinodermata: Asteroidea: Luidiidae) from the Yellow Sea, with a review of two related species. Zootaxa 57–68.

Livingstone, A. A. 1933. Some genera and species of the Asterinidae. Rec. Aust. Museum 19:1–20.

Lombardi, S. A. 2012. Comparative physiological ecology of the Eastern oyster, *Crassostrea virginica*, and the Asian oyster, *Crassostrea ariakensis*: An investigation into aerobic metabolism and hypoxic adaptations. University of Maryland.

Lorenz, F., and C. Melaun. 2011. A new species of *Simnia* from England (Caenogastropoda: Ovulidae). Molluscan Res. 31:167–175.

Lourido, A., L. Gestoso, and J. S. Troncoso. 2006. Assemblages of the molluscan fauna in subtidal soft bottoms of the Ría de Aldán (north-western Spain). J. Mar. Biol. Assoc. United Kingdom 86:129–140.

Lu, H., M. Zhu, and B. Wu. 1994. Ecological and physiological studies of the Antarctic limpet *Nacella concinna* in King George Island. Korean J. Polar Res. 5:1–8.

Lucey, N. M., C. Lombardi, L. Demarchi, A. Schulze, M. C. Gambi, and P. Calosi. 2015. To brood or not to brood: Are marine invertebrates that protect their offspring more resilient to ocean acidification? Sci. Rep. 5:1–7. Nature Publishing Group.

Lutz, R. A., M. Castagna, and J. G. Goodsell. 1982. Larval and early post-larval development of *Arctica islandica*. J. Mar. Biol. Assoc. United Kingdom 62:745–769.

Lützen, J., A. Jespersen, and M. P. Russell. 2014. The Pacific clam *Nutricola tantilla* (Bivalvia: Veneridae) has separate sexes and makes use of brood protection and sperm storage. J. Molluscan Stud. 81:397–406.

Maas, A. E., L. E. Elder, H. M. Dierssen, and B. A. Seibel. 2011. Metabolic response of Antarctic pteropods (Mollusca: Gastropoda) to food depriviation and regional productivity. Mar. Ecol. Prog. Ser. 441:129–139.

MacBride, E. W. 1896. The development of *Asterina gibbosa*. Q. J. Microsc. Sci. 38:339–411.

Macé, A.-M. 1981. Experimental study of the ecophysiology of a boring gastropod, *Polinices alderi* (Forbes). 2. Oxygen consumption and ammoniacal nitrogen excretion. Téthys 10:73–82.

Macé, A.-M., and A. D. Ansell. 1982. Respiration and nitrogen excretion of *Polinices alderi* (Forbes) and *Polinices catena* (da Costa) (Gastropoda: Naticidae). J. Exp. Mar. Bio. Ecol. 60:275–292.

Maciolek, N. J. 1985. A revision of the genus *Prionospio* Malmgren, with special emphasis on species from the Atlantic Ocean, and new records of species belonging to the genera

<i.Apoprionospio</i> Foster and *Paraprionospio* Caullery (Polychaeta, Annelida, Spionida. Zool. J. Linn. Soc. 84:325–383.

Macnae, W. 1962. Notaspidean Opisthobranchiate Molluscs from Southern Africa. Ann. Natal Museum 15:167–181.

Madeira, P., A. Kroh, R. Cordeiro, A. M. DE Frias Martins, and S. P. Ávila. 2019. The echinoderm fauna of the Azores (NE Atlantic Ocean).

Magagnini, G. 1982. Reproduction in *Nerilla antennata* O. Schmidt (Archiannelida Nerillidae): Induction of spawning. Bolletino di Zool. 49:283–286.

Magniez, P. 1983. Reproductive cycle of the brooding echinoid *Abatus cordatus* (Echinodermata) in Kerguelen (Antarctic Ocean): changes in the organ indices, biochemical composition and caloric content of the gonads. Mar. Biol. 74:55–64. Springer-Verlag.

Mak, Y. M. 1995. Egg capsule morphology of five Hong Kong rocky shore littorinids. Hydrobiologia 309:53–59.

Manchenko, G. P., and V. I. Radashevsky. 2002. Genetic differences between two sibling sympatric *Dipolydora* species (Polychaeta: Spionidae) from the Sea of Japan, and a new species description. J. Mar. Biol. Assoc. United Kingdom 82:193–199.

Mangum, C. P. 1963. Studies on speciation in maldanid polychaetes of the North American Atlantic coast—III. Intraspecific and interspecific divergence in oxygen consumption. Comp. Biochem. Physiol. 10:335–349.

Mangum, C. P., and D. M. Miyamoto. 1970. The relation between spontaneous activity cycles and diurnal rhythms of metabolism in the polychaetous annelid *Glycera dibranchiata*. Mar. Biol. 7:7–10.

Manno, C., V. L. Peck, and G. A. Tarling. 2016. Pteropod eggs released at high pCO2 lack resilience to ocean acidification. Sci. Rep. 6:1–10. Nature Publishing Group.

Marina, P., J. Urra, J. de D. Bueno, J. L. Rueda, S. Gofas, and C. Salas. 2020. Spermcast mating with release of zygotes in the small dioecious bivalve *Digitaria digitaria*. Sci. Rep. 10:1–11. Nature Publishing Group UK.

Marinescu, V. P. 1964. La reproduction et le developpement des Polychètes reliques ponto-caspiens du Danube: *Hypaniola kowalewskii* (Grimm) et *Manayunkia caspica*. Rev. Roum. Biol. Ser. Zool. 9:87–100.

Marsden, I. D. 1999. Respiration and feeding of the surf clam *Paphies donacina* from New Zealand. Hydrobiologia 405:179–188.

Marsden, I. D., S. E. Shumway, and D. K. Padilla. 2012. Does size matter? The effects of body size and declining oxygen tension on oxygen uptake in gastropods. J. Mar. Biol. Assoc. United Kingdom 92:1603–1617.

Marsden, J. R. 1992. Reproductive isolation in two forms of the serpulid polychaete, *Spirobranchus polycerus* (Schmarda) in Barbados. Bull. Mar. Sci. 51:14–18.

Marshall, D. J., Y. W. Dong, C. D. McQuaid, and G. A. Williams. 2011. Thermal adaptation in the intertidal snail *Echinolittorina malaccana* contradicts current theory by revealing the crucial roles of resting metabolism. J. Exp. Biol. 214:3649–3657.

Marshall, D. J., P. J. Krug, E. K. Kupriyanova, M. Byrne, and R. B. Emlet. 2012. The biogeography of marine invertebrate life histories. Annu. Rev. Ecol. Evol. Syst. 43:97–114.

Martín, G. S., and T. M. Worsfold. 2015. Guide and keys for the identification of Syllidae (Annelida, Phyllodocida) from the British Isles (reported and expected species). Zookeys 29:1–29.

Martynov, A. V., and N. M. Litvinova. 2008. Deep-water Ophiuroidea of the northern Atlantic with descriptions of three new species and taxonomic remarks on certain genera and species. Mar. Biol. Res. 4:76–111.

Masterman, A. T. 1902. The early development of *Cribrella oculata* (Forbes), with remarks on echinoderm development. Trans. R. Soc. Edinburgh 40:373–417.

Mastrototaro, F., G. Chimienti, A. Matarrese, M. C. Gambi, and A. Giangrande. 2015. Growth and population dynamics of the non-indigenous species *Branchiomma luctuosum* Grube (Annelida, Sabellidae) in the Ionian Sea (Mediterranean Sea). Mar. Ecol. 36:517–529.

Mathivat-Lallier, M. H., and C. Cazaux. 1991. Life-history of *Nephtys hombergii* in Arcachon Bay. Estuar. Coast. Shelf Sci. 32:1–9.

Matsumoto, T. 2018. Mollusca. Pp. 522–525 *in* M. K. Skinner, ed. Encyclopedia of Reproduction. Academic Press.

Matsuo, R., and Y. Ko. 1981. Preliminary notes on the development, growth and maturation of laboratory reared three species of *Hydroides* (Annelida: Polychaeta). Bull. Fac. Fish. Nagasaki Univ. 51:17–22.

Mayzaud, P., M. Boutoute, S. Gasparini, L. Mousseau, and D. Lefevre. 2005. Respiration in marine zooplankton - The other side of the coin: CO2 production. Limnol. Oceanogr. 50:291–298.

Mazurkiewicz, M. 1975. Larval development and habits of *Laeonereis culveri* (Webster) (Polychaeta: Nereidae). Biol. Bull. 149:186–204.

McCarthy, D. A., C. M. Young, and R. H. Emson. 2003. Influence of wave-induced disturbance on seasonal spawning patterns in the sabellariid polychaete *Phragmatopoma lapidosa*. Mar. Ecol. Prog. Ser. 256:123–133.

McClary, D. J., and P. V. Mladenov. 1989. Reproductive pattern in the brooding and broadcasting sea star *Pteraster militaris*. Mar. Biol. 103:531–540.

McClintock, J. B., and B. J. Baker. 1997. Palatability and chemical defense of eggs, embryos and larvae of shallow-water antarctic marine invertebrates. Mar. Ecol. Prog. Ser. 154:121–131.

McClintock, J. B., J. S. Pearse, and I. Bosch. 1988. Population structure and energetics of the shallow-water antarctic sea star *Odontaster validus* in contrasting habitats. Mar. Biol. 99:235–246.

McDonald, J., and L. Marsh. 2005. Asteroidea from shallow waters of the Recherche Archipelago. Pp. 463–476 *in* F. E. Wells, D. I. Walker, and G. A. Kedrick, eds. The Marine Flora and Fauna of Esperance, Western Australia. Western Australian Museum, Perth.

McEdward, L. R., and L. K. Coulter. 1987. Egg volume and energetic content are not correlated among sibling offspring of starfish: Implications for life history theory. Evolution. 41:914.

McEdward, L. R., and B. G. Miner. 2001. Larval and life-cycle patterns in echinoderms. Can. J. Zool. 79:1125–1170.

McEuen, F. S. 1987. Phylum Echinodermata, class Holothuroidea. Pp. 574–596 *in* M. F. Strathmann, ed. Reproduction and Development of Marine Invertebrates of the Northern Pacific Coast. University of Washington Press, Seattle, WA.

McEuen, F. S., and F. S. Chia. 1991. Development and metamorphosis of two psolid sea cucumbers, *Psolus chitonoides* and *Psolidium bullatum*, with a review of reproductive patterns in the family Psolidae (Holothuroidea: Echinodermata). Mar. Biol. 109:267–279.

McEuen, F. S., B. L. Wu, and F. S. Chia. 1983. Reproduction and development of *Sabella media*, a sabellid polychaete with extratubular brooding. Mar. Biol. 76:301–309.

McHugh, D. 1993. A comparative study of reproduction and development in the polychaete family Terebellidae. Biol. Bull. 185:153–167.

McHugh, D. 1989. Population structure and reproductive biology of two sympatric

hydrothermal vent polychaetes, *Paralvinella pandorae* and *P. palmiformis*. Mar. Biol. 103:95–106.
McHugh, D. 1995. Unusual sperm morphology in a deep-sea hydrothermal-vent polychaete *Paralvinella pandorae* (Alvinellidae). Invertebr. Biol. 114:161.
McHugh, D., and P. P. Fong. 2002. Do life history traits account for diversity of polychaete annelids? Invertebr. Biol. 121:325–338.
McHugh, D., and V. Tunnicliffe. 1994. Ecology and reproductive biology of the hydrothermal vent polychaete *Amphisamytha galapagensis* (Ampharetidae). Mar. Ecol. Prog. Ser. 106:111–120.
McKillup, S. C., and A. J. Butler. 1979. Modification of egg production and packaging in response to food availability by *Nassarius pauperatus*. Oecologia 43:221–231.
McLusky, D. S. 1973. The effect of temperature on the oxygen consumption and filtration rate of *Chlamys* (*Aequipecten*) *opercularis* (L.) (bivalvia). Ophelia 10:141–154.
McLusky, D., and A. Stirling. 1975. The oxygen consumption and feeding of *Donax incarnatus* and *Donax spiculum* from tropical beaches. Comp. Biochem. Physiol. -- Part A Physiol. 51:943–947.
McMahon, R. F., W. D. Russell-Hunter, and D. W. Aldridge. 1995. Lack of metabolic temperature compensation in the intertidal gastropods, *Littorina saxatilis* (Olivi) and *L. obtusata* (L.). Hydrobiologia 309:89–100.
Mcnamara, K. J., D. L. Pawson, A. D. Miskelly, and M. Byrne. 2017. Class Echinoidea. Pp. 351–446 *in* T. D. O'Hara and M. Byrne, eds. Australian Echinoderms: Biology, Ecology and Evolution. CSIRO Publishing.
McPherson, B. F. 1968. Feeding and oxygen uptake of the tropical sea urchin *Eucidaris tribuloides* (Lamarck). Biol. Bull. 135:308–321.
McShane, P. E., M. G. Smith, and K. H. H. Beinssen. 1988. Growth and morphometry in abalone (*Haliotis rubra* Leach) from Victoria. Mar. Freshw. Res. 39:161–666.
Ménard, F., F. Gentil, and J. C. Dauvin. 1989. Population dynamics and secondary production of *Owenia fusiformis* Delle Chiaje (Polychaeta) from the Bay of Seine (eastern English Channel). J. Exp. Mar. Bio. Ecol. 133:151–167.
Menge, B. A. 1975. Brood or broadcast? The adaptive significance of different reproductive strategies in the two intertidal sea stars *Leptasterias hexactis* and *Pisaster ochraceus*. Mar. Biol. 31:87–100.
Mercier, A., and J.-F. Hamel. 2013. Reproduction in Asteroidea. Pp. 37–50 *in* J. M. Lawrence, ed. Starfish: Biology and Ecology of the Asteroidea. Johns Hopkins University Press, Baltimore, MD.
Mercier, A., M. A. Sewell, and J. Hamel. 2013. Pelagic propagule duration and developmental mode: reassessment of a fading link. Glob. Ecol. Biogeogr. 22:517–530.
Meretta, P. E., T. Rubilar, M. Cledón, and C. R. R. Ventura. 2014. Geographical implications of seasonal reproduction in the bat star *Asterina stellifera*. J. Sea Res. 85:222–232. Elsevier B.V.
Messina, P., M. Di Filippo, M. C. Gambi, and V. Zupo. 2005. In vitro fertilisation and larval development of a population of *Lumbrineris* (*Scoletoma*) *impatiens* (claparède) (polychaeta, lumbrineridae) of the gulf of naples (italy) in relation to aquaculture. Invertebr. Reprod. Dev. 48:31–40.
Miles, C. M., and K. B. Clark. 2002. Comparison of biochemical composition and developmental mode in two populations of *Costasiella* [Opisthobranchia: Ascoglossa (= Sacoglossa)]. J. Molluscan Stud. 68:101–109.
Miles, C. M., and M. L. Wayne. 2009. Life history trade-offs and response to selection on egg size in the polychaete worm *Hydroides elegans*. Genetica 135:289–298.
Millen, S. V., and T. M. Gosliner. 1985. Four new species of dorid nudibranchs belonging to

the genus *Aldisa* (Mollusca: Opisthobranchia), with a revision of the genus. Zool. J. Linn. Soc. 84:195–233.
Miller, S. E. 1993. Larval period and its influence on post-larval life history: comparison of lecithotrophy and facultative planktotrophy in the aeolid nudibranch *Phestilla sibogae* . Mar. Biol. 117:635–645.
Mills, C. E., and M. F. Strathmann. 1987. Phylum Cnidaria, Class Hydrozoa. Pp. 44–71 *in* M. F. Strathmann, ed. Reproduction and Development of Marine Invertebrates of the Northern Pacific Coast. University of Washington Press, Seattle, WA.
Mills, S., K. Neill, O. Anderson, N. Davey, M. Kelly, and B. Herr. 2017. Extraordinary Echinoderms. National Institute of Water and Atmospheric Research.
Miloslavich, P., and L. Dufresne. 1994. Development and effect of female size on egg and juvenile production in the neogastropod *Buccinum cyaneum* from the Saguenay Fjord. Can. J. Fish. Aquat. Sci. 51:2866–2872.
Miloslavich, P., E. Klein, and P. E. Penchaszadeh. 2003. Reproduction of *Crepidula navicula* Mørch, 1877 and *Crepidula aplysioides* Reeve, 1859 (Caenogastropoda) from Morrocoy and La Restinga Lagoon, Venezuela. Nautilus (Philadelphia). 117:121–134.
Miloslavich, P., and P. Penchaszadeh. 1992. Reproductive biology of *Vermetus* sp. and *Dendropoma corrodens* (Orbigny, 1842) : two vermetid gastropods from the Southern Caribbean. Veliger 35:78–88.
Miloslavich, P., and P. E. Penchaszadeh. 2001. Adelphophagy and cannibalism during early development of *Crucibulum auricula* (Gmelin, 1791) (Gastropoda: Calyptraeidae) from the Venezuelan Caribbean. Nautilus (Philadelphia). 115:39–44.
Miloslavich, P., and P. E. Penchaszadeh. 1994. Spawn and larval development of Engoniophos unicinctus (Say, 1825) (Gastropoda: Prosobranchia) from the southern Caribbean Sea. Veliger 37:425–429.
Minchin, D. 1987. Sea-water temperature and spawning behaviour in the seastar *Marthasterias glacialis*. Mar. Biol. 95:139–143.
Mincks, S. L., P. L. Dyal, G. L. J. Paterson, C. R. Smith, and A. G. Glover. 2009. A new species of *Aurospio* (Polychaeta, Spionidae) from the Antarctic shelf, with analysis of its ecology, reproductive biology and evolutionary history. Mar. Ecol. 30:181–197.
Miura, T., and T. Kajihara. 1984. An ecological study of the life histories of two Japanese serpulid worms *Hydroides ezoensis* and *Pomatoleios kraussii*. Pp. 338–354 *in* P. A. Hutchings, ed. Proceedings of the First International Polychaete Conference. The Linnaean Society of New South Wales, Sydney.
Mjobo, S. 2015. A re-appraisal of the holothuroid genera *Pseudocnus* Panning, 1949 and *Pseudocnella* Thandar, 1987 based on morphological and, for the latter, also molecular evidence (Echinodermata: Holothuroidea: Dendrochirotida: Cucumariidae). University of KwaZulu-Natal.
Mladenov, P. V. 1985. Development and metamorphosis of the brittle star *Ophiocoma pumila*: evolutionary and ecological implications. Biol. Bull. 168:285–295.
Mladenov, P. V., and F. S. Chia. 1983. Development, settling behaviour, metamorphosis and pentacrinoid feeding and growth of the feather star *Florometra serratissima*. Mar. Biol. 73:309–323.
Mladenov, P. V. 1987. Phylum Echinodermata, Class Crinoidea. Pp. 597–605 *in* M. F. Strathmann, ed. Reproduction and Development of Marine Invertebrates of the Northern Pacific Coast. University of Washington Press, Seattle, WA, USA.
Mohan, M. V, and P. V Cheriyan. 1981. Oxygen consumption of *Nausitora hedleyi* Schepman & *Teredo furcifera* Von Martens in relation to body weight. Indian J. Mar. Sci. 10:192–194.
Møhlenberg, F., and T. Kiørboe. 1981. Growth and energetics in *Spisula subtruncata* (Da

Costa) and the effect of suspended bottom material. Ophelia 20:79–90.
Monica Bricelj, V., J. Epp, and R. E. Malouf. 1987. Comparative physiology of young and old cohorts of bay scallop *Argopecten irradians irradians* (Lamarck): mortality, growth, and oxygen consumption. J. Exp. Mar. Bio. Ecol. 112:73–91.
Monro, K., and D. J. Marshall. 2015. The biogeography of fertilization mode in the sea. Glob. Ecol. Biogeogr. 24:1499–1509.
Mooi, R. 1997. Sand dollars of the genus *Dendraster* (Echinoidea: Clypeasteroida): phylogenetic systematics, heterochrony, and distribution of extant species. Bull. Mar. Sci. 61:343–375.
Moran, A. L. 1997. Spawning and larval development of the black turban snail *Tegula funebralis* (Prosobranchia: Trochidae). Mar. Biol. 128:107–114.
Moreau, C., B. Danis, Q. Jossart, M. Eléaume, C. Sands, G. Achaz, A. Agüera, and T. Saucède. 2019. Is reproductive strategy a key factor in understanding the evolutionary history of Southern Ocean Asteroidea (Echinodermata)? Ecol. Evol. 9:8465–8478.
Morley, S. A., T. Hirse, H. O. Pörtner, and L. S. Peck. 2009. Geographical variation in thermal tolerance within Southern Ocean marine ectotherms. Comp. Biochem. Physiol. - A Mol. Integr. Physiol. 153:154–161.
Moroz, L. L. 2011. *Aplysia*. Curr. Biol. 21.
Morris, R. H., D. P. Abbott, and E. C. Haderlie. 1980. Intertidal Invertebrates of California. Stanford University Press.
Mortensen, T. 1928. A monograph of the Echinoidea. Vol.1 Cidaroidea. Copenhagen.
Mortensen, T. 1931. Contribution to the study of development and larval forms of Echinoderms I-II. K. Danske Vidensk. Seiskab Skriffer Naturwidenskbelige Og Math. Afd. 4:1–65.
Mortensen, T. 1938. Contributions to study of the development larval forms of echinoderms IV. K. Danske Vidensk. Selsk. Skr. Naturv. go Math 7:1–59.
Mortensen, T. 1913. Die Echinodermenlarven der Deutschen Südpolar-Expedition 1901-1903 / von Th. Mortensen. Reimer, G, Berlin.
Mortensen, T. 1936. Echinoidea and Ophiuroidea. Cambridge, UK.
Mortensen, T. 1920. On hermaphroditism in viviparous Ophiurids. Acta Zool. 1:1–19.
Mortensen, T. 1921. Studies of the Development and Larval Forms of Echinoderms. Carlsberg Fund, Copenhagen.
Mortensen, T. 1909. The echinoids from the German Antarctic Expedition 1901-1903. Zoologie 3:1–113.
Mortensen, T. 1937. The study of development and larval forms of echinoderms. Mémoires l'Académie R. des Sci. des Lettres Danemark, Copenhague III–IV:406.
Morton, J. E. 1959. The habits and feeding organs of *Dentalium entalis*. J. Mar. Biol. Assoc. United Kingdom 38:225–238. Monash University.
Mukai, H., I. Koike, M. Nishihira, and S. Nojima. 1989. Oxygen consumption and ammonium excretion of mega-sized benthic invertebrates in a tropical seagrass bed. J. Exp. Mar. Bio. Ecol. 134:101–115.
Murdoch, R. C., and S. E. Shumway. 1980. Oxygen consumption in six species of chitons in relation to their position on the shore. Ophelia 19:127–144.
Murray, T. 1982. Morphological characterization of the *Littorina scutulata* species complex. The Veliger 24:233–238.
Murray, F. V. 1962. Notes on the spawn and early life history of two species of *Conuber* Finlay and Marwick, 1937 (Naticidae). J. Malacol. Soc. Aust. 1:49–58.
Muthiga, N. A., and T. R. McClanahan. 2013. *Diadema*. Pp. 257–274 *in* J. M. Lawrence, ed. Sea Urchins: Biology and Ecology. Elsevier.
Naduvaeva, E., and E. Vortsepneva. 2020. General morphology and ultrathin structure of the

gonopericardial complex of *Chaetoderma nitidulum* Lovén, 1844 (Caudofoveata). Zoomorphology 139:21–35. Springer Berlin Heidelberg.
Nagao, Z. 1973. The life history of *Eperetmus typus* Bigelow and the systematics of the family Olindiadidae (Limnomedusae). Publ. Seto Mar. Biol. Lab. 20:89–102.
Navarro, J. M., and R. Torrijos. 1994. Seasonal variation in oxygen uptake and ammonia excretion in the predatory gastropod *Concholepas concholepas* (Bruguiere, 1789). Comp. Biochem. Physiol. -- Part A Physiol. 108:39–46.
Navarro, J. M., and J. E. Winter. 1982. Ingestion rate, assimilation efficiency and energy balance in *Mytilus chilensis* in relation to body size and different algal concentrations. Mar. Biol. 67:255–266.
Nekhaev, I. O. 2013. The first record of *Alvania punctura* from Russian waters (Gastropoda: Rissoidae). Mar. Biodivers. Rec. 6:4–6.
Newell, G. E. 1951. The life history of *Clymenella torquata* (Leidy) (Polychaeta). Proc. Zool. Soc. London 121:561–586.
Newell, R. C., and V. I. Pye. 1971. Variations in the relationship between oxygen consumption, body size and summated tissue metabolism in the winkle *Littorina littorea*. J. Mar. Biol. Assoc. United Kingdom 51:315–338. Monash University.
Nichols, F. H. 1975. Dynamics and energetics of three deposit-feeding benthic invertebrate populations in Puget Sound, Washington. Ecol. Monogr. 45:57–82.
Nicolaidou, A. 1983. Life history and productivity of *Pectinaria koreni* Malmgren (Polychaeta). Estuar. Coast. Shelf Sci. 17:31–43.
Nielsen, A. M., N. T. Eriksen, J. J. Løsnsmann, and H. U. Riisgirdlo. 1995. Feeding, growth and respiration in the polychaetes *Nereis diversicolor* (facultative filter-feeder) and *N. virens* (omnivorous) -- a comparative study. Mar. Ecol. Prog. Ser. 125:149–158.
Nielsen, C. 1998. Origin and evolution of animal life cycles.
Nielsen, C. 1991. The development of the brachiopod *Crania* (*Neocrania*) *anomala* (O. F. Müller) and its phylogenetic significance. Acta Zool. 72:7–28.
Nishi, E. 1992. Occurrence of the boring serpulid Floriprotis sabiuraensis and the brooding serpulid *Paraprotis dendrova* Uchida (Polychaeta : Sedentaria). Galaxea 11:15–20.
Nishi, E. 1996. Serpulid polychaetes associated with living and dead corals at Okinawa Island, southwest Japan. Publ. Seto Mar. Biol. Lab. 37:305–318.
Nishi, E., and M. Nishihira. 1994. Colony formation via sexual and asexual reproduction in *Salmacina dysteri* (Huxley) (Polychaeta, Serpulidae). Zoolog. Sci. 11:589–595.
Nishi, E., and T. Yamasu. 1992a. Brooding habit and larval development of a serpulid worm *Paraprotis dendrova* Uchida (Annelida, Polychaeta, Sedentaria). Bull. Coll. Sci. 54:83–92.
Nishi, E., and T. Yamasu. 1992b. Observation on the reproductive behavior and the development of a common fouling spirorbid, *Dexiospira foraminosa* Bush (Sedentaria, Polychaeta). Bull. Coll. Sci. Univ. Ryukyus 54:101–106.
Niu, C.-J., S. Nakao, and S. Goshima. 1999. Energy requirement for metabolism in a population of the limpet *Lottia kogamogai* (formerly *Collisella heroldi*). Bull. Fac. Fish. Sci. Hokkaido Univ. 50:61–70.
Nival, P., S. Nival, and I. Palazzoli. 1972. Données sur la respiration de différents organismes communs dans le plancton de Villefranche-sur-Mer. Mar. Biol. 17:63–76.
Nojima, S. 1979. Ecological studies of a sea star, *Astropecten latespinosus* Meissner. I. Survivorship curve and life history. Publ. from Amakusa Mar. Biol. Lab. 5:45–65.
Nugranad, J., S. Noodang, W. Ratanachurdchai, K. Promjinda, S. Phanna, and S. Chantara. 2000. Breeding of the Oriental hard clam *Meretrix meretrix* (L., 1758). Phuket Mar. Biol. Cent. Spec. Publ. 21:203–210.
Nuttall, T. 1989. A new *Elysia* (Opisthobranchia: Asgoglossa) from the Florida keys. Veliger

32:302–307.

O' Loughlin, P. M., J. M. Waters, and M. S. Roy. 2002. Description of a new species of *Patiriella* from New Zealand, and review of *Patiriella regularis* (Echinodermata, Asteroidea) based on morphological and molecular data. J. R. Soc. New Zeal. 32:697–711.

O'Connor, W. A., and M. P. Heasman. 1995. Spawning induction and fertilisation in the doughboy scallop *Chlamys* (*Mimachlamys*) *asperrima*. Aquaculture 136:117–129.

O'Connor, W. A., and S. J. O'Connor. 2011. Early ontogeny of the pipi, *Donax* (*Plebidonax*) *deltoides* (Donacidae; Bivalvia). Molluscan Res. 31:53–56.

O'Gorman, E. J., and M. C. Emmerson. 2010. Manipulating interaction strengths and the consequences for trivariate patterns in a marine food web. Pp. 301–419 *in* Advances in Ecological Research.

O'Hara, T. D. 2011. Brittle Star, *Ophiarachnella ramsayi*.

O'Hara, T. D. 2017. Class Ophiuroidea. Pp. 295–350 *in* T. D. O'Hara and M. Byrne, eds. Australian Echinoderms: Biology, Ecology and Evolution. CSIRO.

O'Hara, T. D. 1999. Systematics and biology of Macquarie Island echinoderms. Mem. Museum Victoria 57:167–223.

O'Hara, T. D., and M. Byrne. 2017. Australian Echinoderms: Biology, Ecology and Evolution. CSIRO Publishing.

O'Loughlin, P. M., and F. W. E. Rowe. 2006. A systematic revision of the asterinid genus *Aquilonastra* O'Loughlin, 2004 (Echinodermata: Asteroidea). Mem. Museum Victoria 63:257–287.

O'Loughlin, P. M., J. M. Waters, and M. S. Roy. 2003. A molecular and morphological review of the asterinid, *Patiriella gunnii* (Gray) (Echinodermata: Asteroidea). Mem. Museum Victoria 60:181–195.

Occhioni, G. E., A. C. S. Brasil, and A. F. B. Araújo. 2009. Morphometric study of *Phragmatopoma caudata* (Polychaeta: Sabellida: Sabellariidae). Zoologia 26:739–746.

Oguro, C., M. Komatsu, and Y. T. Kano. 1975. A note on the early development of *Astropecten polyacanthus* Müller et Troschel. Proc. Jap. Soc. Syst. Zool. 11:49–52.

Oguro, C., M. Komatsu, and Y. T. Kano. 1976. Development and metamorphosis of the sea-star, *Astropecten scoparius* Valenciennes. Biol. Bull. 151:560–573.

Ohnheiser, L. T., and M. A. E. Malaquias. 2014. The family Diaphanidae (Gastropoda: Heterobranchia: Cephalaspidea) in Europe, with a redescription of the enigmatic species *Colobocephalus costellatus* M. Sars, 1870. Zootaxa 3774:501–522.

Ohno, T., T. Katoh, and T. Yamasu. 1995. The origin of algal-bivalve photo-symbiosis. Palaeontology 38:1–21.

Okanishi, M., A. Sentoku, S. Fujimoto, N. Jimi, R. Nakayama, Y. Yamana, H. Yamauchi, H. Tanaka, T. Kato, S. Kashio, D. Uyeno, K. Yamamoto, K. Miyazaki, and A. Asakura. 2016. Marine benthic community in Shirahama, southwestern Kii Peninsula, central Japan. Publ. Seto Mar. Biol. Lab. 44:7–52.

Okuda, S. 1946. Studies on the development of Annelida Polychaeta 1. J. Fac. Sci. Hokkaido Imp. Univ. Ser. VI 9:115–219.

Olbers, J., and Y. Samyn. 2012. The *Ophiocoma* species (Ophiurida: Ophiocomidae) of South Africa. West. Indian Ocean J. Mar. Sci. 10:137–154.

Olive, P. J. W. 1970. Reproduction of a Northumberland population of the polychaete *Cirratulus cirratus*. Mar. Biol. 5:259–273.

Oliver, P. G., and A. M. Holmes. 2006. The Arcoidea (Mollusca: Bivalvia): A review of the current phenetic-based systematics. Zool. J. Linn. Soc. 148:237–251.

Olson, R. R., and M. H. Olson. 1989. Food limitation of planktotrophic marine invertebrate larvae: Does it control recruitment success? Annu. Rev. Ecol. Syst. 20:225–247.

Onoda, K. 1931. Notes on the development of *Heliocidaris crassispina* with special reference to the structure of the larval body. Mem. Coll. Sci. Kyoto Imp. Univ. 7:103–134.
Osorio, C., D. Brown, L. Donoso, and H. Atan. 1999. Aspects of the reproductive activity of *Cypraea caputdraconis* from Easter Island (Mollusca: Gastropoda: Cypraeidae). Pacific Sci. 53:15–23.
Osorio, C., C. Gallardo, and H. Atan. 1992. Egg mass and intracapsular development of *Cypraea caputdraconis* Melvill, 1888, from Easter Island (Gastropoda: Cypraeidae). Veliger 35:316–322.
Oug, E. 2012. Guide to identification of Lumbrineridae (Polychaeta) in north east Atlantic waters. Grimstad, Norway.
Oug, E. 1990. Morphology, reproduction, and development of a new species of *Ophryotrocha* (Polychaeta: Dorvilleidae) with strong sexual dimorphism. Sarsia 75:191–201.
Oyarzún, P. A., J. J. Nuñez, J. E. Toro, and J. P. A. Gardner. 2020. Trioecy in the marine mussel *Semimytilus algosus* (Mollusca, Bivalvia): Stable sex ratios across 22 degrees of a latitudinal gradient. Front. Mar. Sci. 7:1–10.
Pain, S. L., P. A. Tyler, and J. D. Gage. 1982a. The reproductive biology of *Hymenaster membranaceus* from the Rockall Trough, North-East Atlantic Ocean, with notes on *H. gennaeus*. Mar. Biol. 70:41–50.
Pain, S. L., P. A. Tyler, and J. D. Gage. 1982b. The reproductive biology of the deep-sea asteroids *Benthopecten simplex* (Perrier), *Pectinaster filholi* Perrier, and *Pontaster tenuispinus* Düben & Koren (Phanerozonia : Benthopectinidae) from the Rockall Trough. J. Exp. Mar. Bio. Ecol. 65:195–211.
Paine, R. T. 1965. Natural History, limiting factors and energetics of the opisthobranch *Navanax inermis*. America (NY). 46:603–619.
Palatzidis, S., S. Yamasaki, H. Hirata, and T. Imai. 1996. Food consumption and growth of *Aplysia dactylomela* Rang (Gastropoda: Opisthobranchia). Aquac. Sci. 44:125–131.
Palmer, A. R. 1990. Effect of crab effluent and scent of damaged conspecifics on feeding, growth, and shell morphology of the Atlantic dogwhelk *Nucella lapillus* (L.). Hydrobiologia 193:155–182.
Palmer, J. B. 1968. An analysis of the distribution of a commensal polynoid on its hosts. University of Oregon.
Pandian, T. J. 2019. Reproduction and Development in Annelida. CRC Press LLC.
Parry, D. M., M. A. Kendall, A. A. Rowden, and S. Widdicombe. 1999. Species body size distribution patterns of marine benthic macrofauna assemblages from contrasting sediment types. J. Mar. Biol. Assoc. United Kingdom 79:793–801.
Patent, D. H. 1970a. Life history of the basket star, *Gorgonocephalus eucnemis* (Müller & Troschel) (Echinodermata; Ophiuroidea). Ophelia 8:145–160.
Patent, D. H. 1970b. The early embryology of the basket star *Gorgonocephalus caryi* (Echinodermata, Ophiuroidea). Mar. Biol. 6:262–267.
Paulet, Y. M., A. Lucas, and A. Gerard. 1988. Reproduction and larval development in two *Pecten maximus* (L.) populations from Brittany. J. Exp. Mar. Bio. Ecol. 119:145–156.
Pawlik, J. R. 1988. Larval settlement and metamorphosis of two gregarious sabellariid polychaetes: *Sabellaria alveolata* compared with *Phragmatopoma californica*. J. Mar. Biol. Assoc. United Kingdom 68:101–124. Monash University.
Paxton, H. 1986. Revision of the *Rhamphobrachium* complex (Polychaeta: Onuphidae). Rec. Aust. Museum 38:75–104.
Paxton, H. 1979. Taxonomy and aspects of the life history of Australian beachworms (Polychaeta: Onuphidae). Mar. Freshw. Res. 30:265–294.
Paz, M., A. Mikhailov, and M. Torrado. 2001. Sexual differentiation of the somatic gonad

tissue in marine bivalve mollusks: Esterase- and fibronectin-like recognition signals. Pp. 119–120 *in* International Journal of Developmental Biology.
Pearse, J. S. 1994. Cold-water echinoderms break "Thorson's Rule." Pp. 26–43 *in* C. M. Young and K. J. Eckelbarger, eds. Reproduction, larval biology, and recruitment of the deep-sea benthos. Columbia University Press, New York.
Pearse, J. S., and J. B. McClintock. 1990. A comparison of reproduction by the brooding spatangoid echinoids *Abatus shackletoni* and *A. nimrodi* in McMurdo Sound, Antarctica. Invertebr. Reprod. Dev. 17:181–191. Taylor & Francis Group.
Pechenik, J. A. 1999. On the advantages and disadvantages of larval stages in benthic marine invertebrate life cycles. Mar. Ecol. Prog. Ser. 177:269–297.
Pedersen, R. V, and L. R. Page. 2000. Development and metamorphosis of the planktotrophic larvae of the moon snail, *Polinices lewisii* (Gould, 1847) (Caenogastropoda: Naticoidea). Veliger 43:58–63.
Pelseneer, P. 1894. Hermaphroditism in Mollusca. Q. J. Microsc. Sci. s2-37:19–46.
Pentreath, R. J. 1971. Respiratory surfaces and respiration in three New Zealand intertidal ophiuroids. J. Zool. 163:397–412.
Percy, J. A. 1972. Thermal adaptation in the boreo-arctic echinoid, *Stronglocentrotus droebachiensis* (O.F. Muller, 1776). I. Seasonal acclimatization of respiration. Physiol. Zool. 45:277–289.
Pereira, R. B., A. A. Almeida, D. M. Pereira, O. Silva, P. B. Andrade, E. Pinto, and P. Valentão. 2018. Trace elements in wild edible *Aplysia* species: Relationship with the desaturation–elongation indexes of fatty acids. Chemosphere 208:682–690.
Pérez Camacho, A., U. Labarta, and E. Navarro. 2000. Energy balance of mussels *Mytilus galloprovincialis*: The effect of length and age. Mar. Ecol. Prog. Ser. 199:149–158.
Pernet, B. 1998. Benthic egg masses and larval development of *Amblyosyllis speciosa* (Polychaeta: Syllidae). J. Mar. Biol. Assoc. United Kingdom 78:1369–1372.
Pernet, B. 1999. Gamete interactions and genetic differentiation among three sympatric polychaetes. Evolution. 53:435.
Pernet, B. 2003. Persistent ancestral feeding structures in nonfeeding annelid larvae. Biol. Bull. 205:295–307.
Pernet, B. 2004. The cryptic filtering house of an invertebrate larva. Science. 306:1757.
Pernet, B., B. T. Livingston, C. Sojka, and D. Lizárraga. 2017. Embryogenesis and larval development of the seastar *Astropecten armatus*. Invertebr. Biol. 1–13.
Perron, F. E., and R. D. Turner. 1977. Development, metamorphosis, and natural history of the nudibranch *Doridella obscura* Verrill (Corambidae: Opisthobranchia). J. Exp. Mar. Bio. Ecol. 27:171–185.
Pettibone, M. H. 1963. Marine polychaete worms of the New England region. I. Aphroditidae through Trochochaetidae.
Pfannenstiel, H.-D., C. Grünig, and J. Lücht. 1987. Gametogenesis and reproduction in nereidid sibling species (*Platynereis dumerilii* and *P. massiliensis*). Bull. Biol. Soc. Wash. 7:272–279.
Phillips, D. W. 1981. Life-history features of the marine intertidal limpet *Notoacmea scutum* (Gastropoda) in central California. Mar. Biol. 64:95–103.
Pillai, T. G. 1958. Studies on a brackish-water polychaetous annelid, *Marphysa borradailei*, sp. n. from Ceylon. Ceylon J. Sci. (Biological Sci. 1:94–106.
Policansky, D. 1982. Sex change in plants and animals. Annu. Rev. Ecol. Syst. 13:471–495.
Pollock, L. W. 1970. *Batillipes dicrocercus* n. sp., *Stygarctus granulatus* n. sp. and other Tardigrada from Woods Hole, Massachusetts, U.S.A. Trans. Am. Microsc. Soc. 89:38.
Pollock, L. W. 1975a. Observations on marine Heterotardigrada, including a new genus from the Western Atlantic Ocean. Cah. Biol. Mar. 17:121–132.

Pollock, L. W. 1975b. Tardigrada. Reprod. Mar. Invertebr. II:43–54.
Pomory, C. M. 2007. Key to the common shallow-water brittle stars (Echinodermata: Ophiuroidea) of the Gulf of Mexico and Caribbean Sea. Caribb. J. Sci. 1–42.
Potswald, H. E. 1968. The biology of fertilization and brood protection in *Spirorbis* (*Laeospira*) *morchi*. Biol. Bull. 135:208–222.
Pradillon, F., N. Le Bris, B. Shillito, C. M. Young, and F. Gaill. 2005. Influence of environmental conditions on early development of the hydrothermal vent polychaete *Alvinella pompejana*. J. Exp. Biol. 208:1551–1561.
Price, A., and F. Rowe. 1996. Indian Ocean echinoderms collected during the Sinbad Voyage (1980-81): 3. Ophiroidea and Echinoidea. Bull. Hist. Museum … 62:71–82.
Price, R., and R. M. Warwick. 1980. Effect of temperature on the respiration rate of meiofauna. Oecologia 44:145–148.
Prince, J. S., and P. M. Johnson. 2013. Role of the digestive gland in ink production in four species of sea hares: An ultrastructural comparison. J. Mar. Biol. 2013:1–5.
Prowse, T. A. A., M. A. Sewell, and M. Byrne. 2008. Fuels for development: Evolution of maternal provisioning in asterinid sea stars. Mar. Biol. 153:337–349.
Przeslawski, R. 2008. Temporal patterns of gastropod egg mass deposition on southeastern Australian shores. Mar. Freshw. Res. 59:457–466.
Qian, P.-Y., and F.-S. Chia. 1989. Sexual reproduction and larval development of *Rhaphidrilus nemasoma* Monticelli, 1910 (Polychaeta: Ctenodrilidae). Can. J. Zool. 67:2345–2351.
Radashevsky, V. I. 1999. Description of the proposed lectotype for *Polydora websteri* Hartman in Loosanoff & Engle, 1943 (Polychaeta: Spionidae). Ophelia 51:107–113.
Radashevsky, V. I. 1989. Ecology, sex determination, reproduction and larval development of the commensal polychaetes *Polydora commensalis* and *Polydora glycymerica* in the Japanese Sea. Pp. 137–164 *in* V. A. Sveshinkov, ed. Symbiosis in Marine Animals. Academy of Sciences USSR, Severtsov Institute of Evolutionary Morphology and Ecology of Animals, Moscow.
Radashevsky, V. I. 1994. Life history of a new *Polydora* species from the Kurile Islands and evolution of lecithotrophy in *Polydorid* genera (Polychaeta: Spionidae). Ophelia 39:121–136.
Radashevsky, V. I. 1988. Morphology, ecology, reproduction, and larval development of *Polydora uschakovi* (Polychaeta, Spionidae) in the Peter the Great Bay of the Sea of Japan. Zool. Zhurnal 67:870–878.
Radashevsky, V. I. 2007. Morphology and biology of a new *Rhynchospio* species (Annelida: Spionidae) from the South China Sea, Vietnam, with the review of *Rhynchospio* taxa. J. Nat. Hist. 41:985–997.
Radashevsky, V. I. 2005. On adult and larval morphology of *Polydora cornuta* Bosc, 1802 (Annelida: Spionidae). Zootaxa 24:1–24.
Radashevsky, V. I., and C. A. Cárdenas. 2004. Morphology and biology of *Polydora rickettsi* (Polychaeta: Spionidae) from Chile. New Zeal. J. Mar. Freshw. Res. 38:243–254.
Radashevsky, V. I., P. Da, and C. Lana. 2009. *Laonice* (Annelida: Spionidae) from South and Central America. Zoosymposia 295:265–295.
Radashevsky, V. I., M. Díaz, and C. Bertrán. 2006a. Morphology and biology of *Prionospio patagonica* (Annelida: Spionidae) from Chile. J. Mar. Biol. Assoc. United Kingdom 86:61–69.
Radashevsky, V. I., P. C. Lana, and R. C. Nalesso. 2006b. Morphology and biology of *Polydora* species (Polychaeta: Spionidae) boring into oyster shells in South America, with the description of a new species. Zootaxa 1–37.
Radashevsky, V. I., and A. E. Migotto. 2009. Morphology and biology of a new

Pseudopolydora (annelida: Spionidae) species from Brazil. J. Mar. Biol. Assoc. United Kingdom 89:461–468.

Radashevsky, V. I., and J. M. D. M. Nogueira. 2003. Life history, morphology and distribution of *Dipolydora armata* (Polychaeta: Spionidae). J. Mar. Biol. Assoc. United Kingdom 83:375–384.

Radashevsky, V. I., and C. Olivares. 2005. *Polydora uncinata* (Polychaeta : Spionidae) in Chile: An accidental transportation across the Pacific. Biol. Invasions 7:489–496.

Rakaj, A., A. Fianchini, P. Boncagni, M. Scardi, and S. Cataudella. 2019. Artificial reproduction of *Holothuria polii*: A new candidate for aquaculture. Aquaculture 498:444–453.

Ralph, R., and J. Maxwell. 1977a. The oxygen consumption of the Antarctic limpet *Nacella* (*Patinigera*) *concinna*. Br. Antarct. Surv. Bull. 45:19–23.

Ralph, R., and J. G. H. Maxwell. 1977b. The oxygen consumption of the Antarctic lamellibranch *Gaimardia trapesina* trapesina in relation to cold adaptation in polar invertebrates. Br. Antart. Surv. Bull. 45:41–46.

Ramey, P. 2008. Life history of a dominant polychaete, *Polygordius jouinae*, in inner continental shelf sands of the Mid-Atlantic Bight, USA. Mar. Biol. 154:443–452.

Ramofafia, C., M. Byrne, and S. C. Battaglene. 2003. Development of three commercial sea cucumbers, *Holothuria scabra*, *H. fuscogilva* and *Actinopyga mauritiana*: larval structure and growth. Mar. Freshw. Res. 54:657–667.

Rampal, J. 1975. Les Thécosomes (mollusques pélagiques) Systématique et Evolution - Ecologie et Biogéographie Méditerranéennes. University of Aix-Marseille I, Marseille.

Rao, K. V. 1961. Development and life history of a nudibranchiate gastropod *Cuthona adyarensis* Rao. J. Mar. Biol. Assoc. India 3:186–197.

Rasmussen, E. 1956. Faunistic and biological notes on marine invertebrates III. Biol. Meddelelser det K. Dansk Vidensk. Selsk. 23:1–84.

Rasmussen, E. 1973. Systematics and ecology of the Isefjord marine fauna (Denmark): with a survey of the eelgrass (*Zostera*) vegetation and its communities. Ophelia 11:1–507.

Raup, D. M. 1965. Crystal orientations in the echinoid apical system. J. Paleontol. 39:934–951.

Read, G. B. 1974. Egg masses and larvae of the polychaete *Nereis falcaria* (note). New Zeal. J. Mar. Freshw. Res. 8:557–561.

Read, G. B. 1975. Systematics and biology of polydorid species (Polychaeta: Spionidae) from Wellington Harbour. J. R. Soc. New Zeal. 5:395–419.

Reed, C. G. 1987. Phylum Brachiopoda. Pp. 486–493 *in* M. F. Strathmann, ed. Reproduction and Development of Marine Invertebrates of the Northern Pacific Coast Book. University of Washington Press, Seattle, WA, USA.

Rehfeldt, N. 1968. Reproductive and morphological variations in the prosobranch "<i>Rissoa membranacea’/i>". Ophelia 5:157–173.

Reid, D. G. 2007. The genus *Echinolittorina* Habe, 1956 (Gastropoda: Littorinidae) in the Indo-West Pacific Ocean. Zootaxa 1–161.

Reid, J. D. 1964. The reproduction of the sacoglossan opisthobranch *Elysia maoria*. Proc. Zool. Soc. London 143:365–393.

Reish, D. J. 1980. The effect of different pollutants on ecologically important polychaete worms. Ann Arbor, Michigan, USA.

Reish, D. J. 1957. The life history of the polychaetous annelid *Neanthes caudata* (delle Chiaje), including a summary of development in the family Nereidae. Pacific Sci. 11:216–228.

Reynoldson, T. B. 1939. On the life-history and ecology of *Lumbricillus lineatus* Mull. (Oligochaeta). Ann. Appl. Biol. 26:782–799.

Rice, M. E. 1975. Sipuncula. Pp. 67–127 *in* A. C. Giese and J. S. Pearse, eds. Reproduction of Marine Invertebrates. Academic Press Inc., New York.

Richards, S. L. 1970. Spawning and reproductive morphology of *Scolelepis squamata* (Spionidae: Polychaeta). Can. J. Zool. 48:1369–1379.

Robilliard, G. A. 1972. A new species of *Dendronotus* from the northeastern Pacific with notes on *Dendronotus nanus* and *Dendronotus robustus* (Mollusca: Opisthobranchia). Can. J. Zool. 50:421–432.

Robinson, L. A., S. P. R. Greenstreet, H. Reiss, R. Callaway, J. Craeymeersch, I. De Boois, S. Degraer, S. Ehrich, H. M. Fraser, A. Goffin, I. Kröncke, L. L. Jorgenson, M. R. Robertson, and J. Lancaster. 2010. Length-weight relationships of 216 North Sea benthic invertebrates and fish. J. Mar. Biol. Assoc. United Kingdom 90:95–104.

Rodhouse, P. G. 1978. Energy transformations by the oyster *Ostrea edulis* L. in a temperate estuary. J. Exp. Mar. Bio. Ecol. 34:1–22.

Roe, P. 1975. Aspects of life history and of territorial behavior in young individuals of *Platynereis bicanaliculata* and *Nereis vexillosa* (Annelida, Polychaeta). Pacific Sci. 29:341–348.

Ropert, M., and P. Goulletquer. 2000. Comparative physiological energetics of two suspension feeders: Polychaete annelid Lanice conchilega (Pallas 1766) and Pacific cupped oyster Crassostrea gigas (Thunberg 1795). Aquaculture 181:171–189.

Ropes, J. W. 1982. Hermaphroditism, sexuality and sex ratio in the surf clam *Spisula solidissitna* and the soft shell clam *Mya arenaria*. Nautilus (Philadelphia). 96:141–146.

Rose, R. 1985. Spawning and development of Opisthobranchs. Proc. Linn. Soc. NSW 108:23–36.

Rouse, G. 1994. New species of *Oriopsis* Caullery and Mesnil from Florida, Belize, and Aldabra Atoll (Seychelles), and a new species of *Amphiglena* Claparède from Seychelles (Polychaeta: Sabellidae: Sabellinae). Bull. Mar. Sci. 54:180–202.

Rouse, G., and K. Fitzhugh. 1994. Broadcasting fables: Is external fertilization really primitive? Sex, size, and larvae in sabellid polychaetes. Zool. Scr. 23:271–312.

Rouse, G. W. 1996a. A new species of *Perkinsiana* (Sabellidae, Polychaeta) from Papua New Guinea; with a description of larval development. Ophelia 45:101–114.

Rouse, G. W. 1993a. *Amphiglena terebro* sp nov. (Polychaeta: Sabellidae: Sabellinae) from eastern australia; Including a description of larval development and sperm ultrastructure. Ophelia 37:1–18.

Rouse, G. W. 2000. Bias? What bias? The evolution of downstream larval-feeding in animals. Zool. Scr. 29:213–236.

Rouse, G. W. 1990a. Four new species of *Micromaldane* (Polychaeta: Maldanidae) from eastern Australia. Rec. Aust. Museum 42:209–219.

Rouse, G. W. 1996b. New *Fabriciola* and *Manayunkia* species (Fabriciinae: Sabellidae: Polychaeta) from Papua New Guinea. J. Nat. Hist. 30:1761–1778.

Rouse, G. W. 1993b. New *Fabriciola species* (Polychaeta, Sabellidae, Fabriciinae) from the eastern Atlantic, with a description of sperm and spermathecal ultrastructure. Zool. Scr. 22:249–261.

Rouse, G. W. 1990b. New species of *Oriopsis* and a new record for *Augeneriella* cf. *dubia* Hartmann-Schröder, 1965 from eastern Australia (Polychaeta: Sabellidae). Rec. Aust. Museum 42:221–235.

Rouse, G. W., and M. C. Gambi. 1997. Cladistic relationships within *Amphiglena* Claparède (Polychaeta: Sabellidae) with a new species and a redescription of *A. mediterranea* (Leydig). J. Nat. Hist. 31:999–1018.

Rouse, G. W., and M. C. Gambi. 1998. Evolution of reproductive features and larval development in the genus *Amphiglena* (Polychaeta: Sabellidae). Mar. Biol. 131:743–

754.
Rouse, G. W., and F. Pleijel. 2001a. Polychaeta. OUP Oxford, Oxford, U.K.
Rouse, G. W., and F. Pleijel. 2001b. Polychaetes. Oxford University Press, New York.
Rouse, G. W., and F. Pleijel. 2006. Reproductive Biology and Phylogeny of Annelida. 4th ed. Science Publishers, Enfield, NH USA.
Rowe, F. W. E., T. D. O'Hara, and T. M. Bardsley. 2017. Class Holothuroidea. Pp. 447–490 *in* T. D. O'Hara and M. Byrne, eds. Australian Echinoderms: Biology, Ecology and Evolution. CSIRO Publishing.
Ruiz-Velásquez, M., M. Zapata, M. T. Gonzalez, D. Ordenes, and M. Escalona. 2017. Sexual differentiation and size at first maturity of the mussel *Choromytilus chorus* (Molina, 1782) (Mollusca, Bivalvia) in Northern Chile. Am. Malacol. Bull. 35:31–41.
Rumohr, H., T. Brey, and S. Ankar. 1987. A compilation of biometric conversion factors for benthic invertebrates of the Baltic Sea. Balt. Mar. Biol. 9:1–56.
Rumrill, S. S., and J. S. Pearse. 2020. Contrasting reproductive periodicities among north-eastern Pacific ophiuroids. Echinodermata 633–638.
Run, J. Q., C. P. Chen, K. H. Chang, and F. S. Chia. 1988. Mating behaviour and reproductive cycle of *Archaster typicus* (Echinodermata: Asteroidea).
Ruppert, E. E., R. S. Fox, and R. D. Barnes. 2004. Invetebrate Zoology - A Functional Evolutionary Approach. 7th ed. Brooks/Cole - Thompson Learning, Belmont, USA.
Ruta, C., and F. Pleijel. 2006. A revision of *Syllidia* (Psamathini, Hesionidae, Polychaeta). J. Nat. Hist. 40:503–521.
Rutherford, J. C. 1977. Geographical variation in morphological and electrophoretic characters in the holothurian *Cucumaria curata*. Mar. Biol. 43:165–174.
Rutherford, J. C. 1973. Reproduction, growth and mortality of the holothurian *Cucumaria pseudocurata*. Mar. Biol. 22:167–176.
Ryabushko, V. I., and L. N. Propp. 1985. Rates of respiration and nitrogen and phosphorous metabolism in echinoderms in the South China Sea. Sov. J. Mar. Biol. 11:331–335.
Ryabushko, V. I., V. G. Tarasov, I. I. Cherbadzhi, and L. I. Ryabushko. 1981. Respiration of sand dollars in the community of mobile sestonophages. Sov. J. Mar. Biol. 7:320–325.
Rzhavsky, A. V., and E. K. Kupriyanova. 2019. Evolution of spirorbin brooding: A phylogenetic analysis and a test of an oxygen limitation hypothesis. Invertebr. Zool. 16:409–430.
Saleuddin, A. S. M. 1964. The gonads and reproductive cycle of *Astarte sulcata* (da Costa) and sexuality in *A. elliptica* (Brown). J. Molluscan Stud. 36:141–148.
Salzwedel, H. 1979. Zur Ökologie von Tellina fabula Gmelin (Bivalvia) in der Deutschen Bucht. University of Kiel, Germany.
Sarà, A., C. Cerrano, and M. Sarà. 2002. Viviparous development in the Antarctic sponge *Stylocordyla borealis* Loven, 1868. Polar Biol. 25:425–431.
Sarma, A. 1975. Three new species of the bivalved gastropods *Julia* and *Berthelinia* found in eastern Indian Ocean. Venus (Japanese J. Malacol. 34:11–25.
Sastry, A. N. 1979. Pelecypoda (excluding Ostreidae). Pp. 113–292 *in* A. C. Giese and J. S. Pearse, eds. Reproduction of Marine Invertebrates. Academic Press.
Sato-Okoshi, W. 1998. Three new species of polydorids (Polychaeta, Spionidae) from Japan. Species Divers. 3:277–288.
Sato-Okoshi, W., and K. Okoshi. 1997. Survey of the genera *Polydora*, *Boccardiella* and *Boccardia* (Polychaeta, Spionidae) in Barkley sound (Vancouver Island, Canada), with special reference to boring activity. Bull. Mar. Sci. 60:482–493.
Sato-Okoshi, W., Y. Sugawara, and T. Nomura. 1990. Reproduction of the boring polychaete *Polydora variegata* inhabiting scallops in Abashiri Bay, North Japan. Mar. Biol. 104:61–66.

Sato, M., and K. Osanai. 1996. Role of jelly matrix of egg masses in fertilization of the polychaete *Lumbrineris latreilli*. Invertebr. Reprod. Dev. 29:185–191.

Schade, H., N. Arneth, M. Powilleit, and S. Forster. 2019. Sand gapers' breath: Respiration of *Mya arenaria* (L. 1758) and its contribution to total oxygen utilization in sediments. Mar. Environ. Res. 143:101–110. Elsevier.

Schaefer, K., and B. Ruthensteiner. 2001. The cephalic sensory organ in pelagic and intracapsular larvae of the primitive opisthobranch genus *Haminoea* (Mollusca: Gastropoda). Zool. Anz. 240:69–82.

Scheibling, R. E., and J. M. Lawrence. 1982. Differences in reproductive strategies of morphs of the genus *Echinaster* (Echinodermata: Asteroidea) from the Eastern Gulf of Mexico. Mar. Biol. 70:51–62.

Scheltema, R. S. 1995. Architectonicidae of the Indo-Pacific (Mollusca, Gastropoda). J. Exp. Mar. Bio. Ecol. 188:145–146.

Scheltema, R., I. Williams, M. Shaw, and C. Loudon. 1981. Gregarious settlement by the larvae of *Hydroides dianthus* (Polychaeta, Serpulidae). Mar. Ecol. Prog. Ser. 5:69–74.

Schmekel, L., and A. Portmann. 1982. Opisthobranchia des Mittelmeeres. Springer-Verlag, Berlin.

Schmid, M. K. 1996. On the distribution and oxygen consumption of ecologically important benthic animals in the waters around Svalbard (Arctic). Berichte zur Polarforsch. 202:1–93.

Schmid, M. K., and D. Piepenburg. 1993. The benthos zonation of the Disko Fjord, West Greenland. Meddelelser om Gronland, Biosci. 37:1–23.

Schmitt, R. J. 1979. Mechanics and timing of egg capsule release by the littoral fringe periwinkle *Littorina planaxis* (Gastropoda: Prosobranchia). Mar. Biol. 50:359–366.

Schneider, K., J. Silverman, B. Kravitz, T. Rivlin, A. Schneider-Mor, S. Barbosa, M. Byrne, and K. Caldeira. 2013. Inorganic carbon turnover caused by digestion of carbonate sands and metabolic activity of holothurians. Estuar. Coast. Shelf Sci. 133:217–223. Elsevier Ltd.

Schneider, S., A. Fischer, and A. W. C. Dorresteijn. 1992. A morphometric comparison of dissimilar early development in sibling species of *Platynereis* (Annelida, Polychaeta). Roux's Arch. Dev. Biol. 201:243–256.

Schoener, A. 1972. Fecundity and possible mode of development of some deep-sea ophiuroids. Limnol. Oceanogr. 17:193–199.

Schram, J. B., J. B. McClintock, R. A. Angus, and J. M. Lawrence. 2011. Regenerative capacity and biochemical composition of the sea star *Luidia clathrata* (Say) (Echinodermata: Asteroidea) under conditions of near-future ocean acidification. J. Exp. Mar. Bio. Ecol. 407:266–274. Elsevier B.V.

Schram, T. A., and B. Haaland. 1984. Larval development and metamorphosis of *Nereimyra punctata* (O.F. Müller) (Hesionidae, Polychaeta). Sarsia 69:169–181.

Schroeder, P. C., and C. O. Hermans. 1975a. Annelida: Polychaeta. Pp. 1–213 *in* A. C. Giese and J. S. Pearse, eds. Reproduction of Marine Invertebrates. Academic Press Inc.

Schroeder, P. C., and C. O. Hermans. 1975b. Annelida: Polychaeta. Pp. 1–213 *in* A. C. Giese and J. S. Pearse, eds. Reproduction of Marine Invertebrates. Vol III. ACADEMIC PRESS, INC.

Schultz, H. A. G. 2017. Echinoidea: With Bilateral Symmetry. Irregularia. 2nd ed. De Gruyter, Boston, MA, USA.

Schultz, H. A. G. 2015. Echinoidea. 1st ed. De Gruyter.

Schulze, A. 2006. Phylogeny and genetic diversity of Palolo worms (*Palola*, Eunicidae) from the tropical North Pacific and the Caribbean. Biol. Bull. 210:25–37.

Schulze, A., A. Maiorova, L. E. Timm, and M. E. Rice. 2012. Sipunculan larvae and

"cosmopolitan" species. Pp. 497–510 *in* Integrative and Comparative Biology.
Sedova, L. G. 2000. The effect of temperature on the rate of oxygen consumption in the sea urchin *Strongylocentrotus intermedius*. Russ. J. Mar. Biol. 26:51–53.
Segrove, F. 1941. The development of the serpulid *Pomatoceros triqueter* L. J. Cell Sci. s2-82:467–540.
Seibel, B. A., and H. M. Dierssen. 2003. Cascading trophic impacts of reduced biomass in the Ross Sea, Antarctica: Just the tip of the iceberg? Biol. Bull. 205:93–97.
Seitz, R. D., and L. C. Schaffner. 1995. Population ecology and secondary production of the polychaete *Loimia medusa* (Terebellidae). Mar. Biol. 121:701–711.
Sejr, M. K., J. K. Petersen, K. T. Jensen, and S. Rysgaard. 2004. Effects of food concentration on clearance rate and energy budget of the Arctic bivalve *Hiatella arctica* (L) at subzero temperature. J. Exp. Mar. Bio. Ecol. 311:171–183.
Sekizawa, A., S. Seki, M. Tokuzato, S. Shiga, and Y. Nakashima. 2013. Disposable penis and its replenishment in a simultaneous hermaphrodite. Biol. Lett. 9:15–18.
Sella, G., M. C. Premoli, and F. Turri. 1997. Egg trading in the simultaneously hermaphroditic polychaete worm *Ophryotrocha gracilis* (Huth). Behav. Ecol. 8:83–86.
Selvakumaraswamy, P., and M. Byrne. 2000. Reproduction, spawning, and development of 5 ophiuroids from Australia and New Zealand. Invertebr. Biol. 119:394–402.
Sendall, K., and S. I. Salazar-Vallejo. 2013. Revision of *Sternaspis* Otto, 1821 (Polychaeta, Sternaspidae). Zookeys 286:1–74.
Sewell, M. A. 1994. Small size, brooding, and protandry in the apodid sea cucumber *Leptosynapta clarki*. Biol. Bull. 187:112–123.
Shain, D. H. 2009. Annelids in Modern Biology.
Shick, J. M., W. F. Taylor, and A. N. Lamb. 1981. Reproduction and genetic variation in the deposit-feeding sea star *Ctenodiscus crispatus*. Mar. Biol. 63:51–66.
Shirayama, Y. 1992. Respiration rates of bathyal meiobenthos collected using a deep-sea submersible SHINKAI 2000. Deep Sea Res. Part A, Oceanogr. Res. Pap. 39:781–788.
Shojaei, M. G., L. Gutow, J. Dannheim, H. Pehlke, and T. Brey. 2015. Functional Diversity and Traits Assembly Patterns of Benthic Macrofaunal Communities in the Southern North Sea. Pp. 183–195 *in* G. Lohmann, H. Meggers, V. Unnithan, D. Wolf-Gladrow, J. Notholt, and A. Bracher, eds. Towards an Interdisciplinary Approach in Earth System Science: Advances of a Helmholtz Graduate Research School. Springer International Publishing Switzerland.
Shumway, S. E. 1983. Factors affecting oxygen consumption in the coot clam *Mulinia lateralis* (Say). Ophelia 22:143–171.
Shumway, S. E. 1979. The effects of body size, oxygen tension and mode of life on the oxygen uptake rates of polychaetes. Comp. Biochem. Physiol. -- Part A Physiol. 64:273–278.
Shumway, S. E., C. Bogdanowiczt, and D. Dean. 1988. Oxygen consumption and feeding rates of the sabellid polychaete, *Myxicola infundibulum*. Exp. Anim. 90A:425–428.
Shumway, S., and R. Koehn. 1982. Oxygen consumption in the American oyster *Crassostrea virginica*. Mar. Ecol. Prog. Ser. 9:59–68.
Siikavuopio, S. I., A. Mortensen, and J. S. Christiansen. 2008. Effects of body weight and temperature on feed intake, gonad growth and oxygen consumption in green sea urchin, *Strongylocentrotus droebachiensis*. Aquaculture 281:77–82.
Simon, J. L. 1967. Reproduction and larval development of *Spio setosa* (Spionidae: Polychaeta). Bull. Mar. Sci. 17:398–431.
Simone, L., G. Pastorino, and P. Penchaszadeh. 2000. *Crepidula argentina*, (Gastropoda; Calyptraeidae) a new species from Argentine littoral waters with description of the anatomy and reproductive pattern. The Veliger 114:127–141.

Simonini, R., G. Massamba-N'siala, V. Grandi, and D. Prevedelli. 2009. Distribution of the genus *Ophryotrocha* (Polychaeta) in Italy: New records and comments on the biogeography of Mediterranean species. Vie Milieu 59:79–88.
Simpson, M. 1962. Reproduction of the polychaete *Glycera dibranchiata* at Solomons, Maryland. Biol. Bull. 123:396–411.
Simpson, R. D. 1982. Papers from the Echinoderm Conference. 3. The reproduction of some echinoderms from Macquarie Island. Pap. from Echinoderm Conf. Aust. Museum Mem. 16:39–52. Trustees of the Australian Museum, Sydney, NSW.
Sirenko, B. I. 2015. The enigmatic viviparous chiton *Calloplax vivipara* (Plate, 1899) (Mollusca: Polyplacophora) and a survey of the types of reproduction in chitons. Russ. J. Mar. Biol. 41:24–31.
Sisson, C. G. 2005. Veligers from the nudibranch *Dendronotus frondosus* show shell growth and extended planktonic period in laboratory culture. Hydrobiologia 541:205–213.
Smith, B. J., and R. L. Jensz. 1968. Unusual mode of reproduction in a new species of polychaete.
Smith, I. F. 2015. *Phorcus lineatus*, Identification & Biology (Unpublished). https://doi.org/10.13140/2.1.5005.0881.
Smith, K. L. 1985. Deep-sea hydrothermal vent mussels: Nutritional state and distribution at the Galapagos Rift. Ecology 66:1067–1080.
Smith, K. L. 1983. Metabolism of two dominant epibenthic echinoderms measured at bathyal depths in the Santa Catalina Basin. Mar. Biol. 72:249–256.
Smith, R. 1984. Development and settling of *Spirobranchus giganteus* (Polychaeta; Serpulidae). Pp. 461–483 *in* P. A. Hutchings, ed. Proceedings of the First International Polychaete Conference. Linnean Society of New South Wales, Sydney.
Smolensky, N., M. R. Romero, and P. J. Krug. 2009. Evidence for costs of mating and self-fertilization in a simultaneous hermaphrodite with hypodermic insemination, the opisthobranch *Alderia willowi*. Biol. Bull. 216:188–199.
Sofian, A., A. Suhermanto, S. Saidin, M. Sayuti, D. Novianto, and F. Widyasari. 2021. Short communication: Environment and morphometric of sea hare *Dolabella auricularia* from shrimp pond, Sorong, West Papua, Indonesia. Biodiversitas J. Biol. Divers. 22:983–987.
Soliman, E., and S. Nojima. 1984. Some observations on dispersal behaviour of the early juvenile stage of the sea-star, *Asterina minor* Hayashi. Publ. Amakusa makusa Mar. Biol. Lab. Kyushu Univ. 7:81–93.
Solis Marin, F. A. 2003. Systematics and phylogeny of the holothurian family Synallactidae. University of Southhampton.
Solsona, M., and J. Martinell. 1999. Protoconch as a taxonomic tool in Gastropoda systematics. Application in the Pliocene Mediterranean Naticidae. Geobios 32:409–419.
Son, M. H., and S. Y. Hong. 1998. Reproduction of *Littorina brevicula* in Korean waters. Mar. Ecol. Prog. Ser. 172:215–223.
Soong, K., D. Chang, and S. M. Chao. 2005. Presence of spawn-inducing pheromones in two brittle stars (Echinodermata: Ophiuroidea). Mar. Ecol. Prog. Ser. 292:195–201.
Southgate, P. C., and J. S. Lucas. 2008. The Pearl Oyster. Elsevier Science.
Southward, E. C., and A. J. Southward. 1958. The breeding of *Arenicola ecaudata* Johnston and *A. banchialis* Aud. & Edw. at Plymouth. J. Mar. Biol. Assoc. United Kingdom 37:268–285. Monash University.
Spight, T. M. 1976. Ecology of hatching size for marine snails. Oecologia 24:283–294.
Spight, T. M., and J. Emlen. 1976. Population sizes of two marine snails with a changing food supply. Ecology 57:1162–1178.
Sriwoon, R., and N. Poonsud. 2019. Growth and reproductive of *Babylonia areolata* Link 1807 in Songkhla and Pattani, the lower part of Gulf of Thailand. Songklanakarin J. Sci.

Technol. 41:1402–1410.
Stancyk, S. E. 1973. Development of *Ophiolepis elegans* (Echinodermata: Ophiuroidea) and its implications in the estuarine environment. Mar. Biol. 21:7–12.
Stead, R. A., and R. J. Thompson. 2003. Physiological energetics of the protobranch bivalve *Yoldia hyperborea* in a cold ocean environment. Polar Biol. 26:71–78.
Stewart, B., and P. V Mladenov. 1994. Aspects of reproduction and anatomy of the euryalinid snake star *Astrohrachion constrictum*. Pp. 491–497 *in* B. David, A. Guille, J.-P. Feral, and M. Roux, eds. Echinoderms through Time. Balkema, Rotterdam, Netherlands.
Stigzelius, J., A. Laine, J. Rissanen, A. B. Andersin, and E. Ilus. 1997. The introduction of *Marenzelleria viridis* (Polychaeta, Spionidae) into the Gulf of Finland and the Gulf of Bothnia (northem Baltic Sea). Ann. Zool. Fennici 34:205–212.
Stöhr, S. 2004. *Ophiuraster patersoni* Litvinova, 1998 is the postlarva of *Ophiomyxa serpentaria* Lyman, 1883 (Echinodermata, Ophiuroidea). Zoosystema 26:95–105.
Stöhr, S., and A. Martynov. 2016. Paedomorphosis as an evolutionary driving force: Insights from deep-sea brittle stars. PLoS One 11:1–24.
Strathmann, M. F. 1987a. Phylum Mollusca, Class Gastropoda, Subclass Prosobranchia. Pp. 220–267 *in* M. F. Strathmann, ed. Reproduction and Development of Marine Invertebrates of the Northern Pacific Coast. University of Washington Press, Seattle, WA.
Strathmann, M. F. 1987b. Reproduction and Development of Marine Invertebrates of the Northern Pacific Coast. University of Washington Press, Seattle.
Strathmann, M. F., R. L. Fernald, C. O. Hermans, T. C. Lacalli, J. Wilson, W H, and S. A. Woodin. 1987. Phylum Annelida, Class Polychaeta. Pp. 138–195 *in* M. F. Strathmann, ed. Reproduction and Development of Marine Invertebrates of the NorthernPacific Coast. University of Washington Press, Seattle, WA.
Strathmann, M. F., and S. S. Rumrill. 1987. Phylum Echinodermata, class Ophiuroidea. Pp. 556–573 *in* M. F. Strathmann, ed. Reproduction and Development of Marine Invertebrates of the Northern Pacific Coast. University of Washington Press, Seattle, WA.
Strathmann, R. R. 1971. The feeding behavior of planktotrophic echinoderm larvae: Mechanisms, regulation, and rates of suspension feeding. J. Exp. Mar. Bio. Ecol. 6:109–160.
Strathmann, R. R. 1974. The spread of sibling larvae of sedentary marine invertebrates. Am. Nat. 108:29–44.
Strathmann, R. R., L. Fenaux, and M. F. Strathmann. 1992. Heterochronic developmental plasticity larval sea urchins and its implications for evolution of nonfeeding larvae. Evolution. 46:972–986.
Styan, C. A., and A. J. Butler. 2003. Asynchronous patterns of reproduction for the sympatric scallops *Chlamys bifrons* and *Chlamys asperrima* (Bivalvia: Pectinidae) in South Australia. Mar. Freshw. Res. 54:77–86.
Styan, C. A., and A. J. Butler. 2000. Fitting fertilisation kinetics models for free-spawning marine invertibrates. Mar. Biol. 137:943–951.
Suckling, C. C., and J. Richard. 2020. Short-term exposure to storm-like scenario microplastic and salinity conditions does not impact adult sea urchin (*Arbacia punctulata*) physiology. Arch. Environ. Contam. Toxicol. 78:495–500. Springer US.
Sukhn, C. M. 2013. Bioaccumulation and depuration in sea urchins *Paracentrotus lividus* (Lebanon) and *Heliocidaris erythrogramma* (Australia). University of New South Wales.
Sukhotin, A. A. 1992. Respiration and energetics in mussels (*Mytilus edulis* L.) cultured in the White Sea. Aquaculture 101:41–57.

Sukhotin, A. A., and H. O. Pörtner. 2001. Age-dependence of metabolism in mussels *Mytilus edulis* (L.) from the White Sea. J. Exp. Mar. Bio. Ecol. 257:53–72.
Sumida, P. Y. G., P. A. Tyler, R. S. Lampitt, and J. D. Gage. 2000. Reproduction, dispersal and settlement of the bathyal ophiuroid *Ophiocten gracilis* in the NE Atlantic Ocean. Mar. Biol. 137:623–630.
Sunday, J., L. Raeburn, H. Stewart, and M. W. Hart. 2009. Allelic inheritance in naturally occurring parthenogenetic offspring of the gonochoric sea star *Patiria miniata*. Invertebr. Biol. 128:276–282.
Sveshnikov, V. A. 1968. Pelagic larvae of some Polychaeta in the White Sea (Pelagicheskie Lichinki Nekotorykh Polikhet Belogo Morya).
Switzer-Dunlap, M., and M. G. Hadfield. 1977. Observations on development, larval growth and metamorphosis of four species of Aplysiidae (Gastropoda: Opisthobranchia) in laboratory culture. J. Exp. Mar. Bio. Ecol. 29:245–261.
Switzer-Dunlap, M., K. Meyers-Schulte, and E. A. Gardner. 1984. The effect of size, age, and recent egg laying on copulatory choice of the hermaphroditic mollusc *Aplysia juliana*. Int. J. Invertebr. Reprod. Dev. 7:217–225.
Tagliarolo, M., F. Porri, and U. M. Scharler. 2018. Temperature-induced variability in metabolic activity of ecologically important estuarine macrobenthos. Mar. Biol. 165:1–13.
Takano, T., Y. M. Hirano, C. D. Trowbridge, Y. J. Hirano, and Y. Watano. 2013. Taxonomic clarification in the genus *Elysia* (Gastropoda: Sacoglossa): *E. atroviridis* and *E. setoensis*. Am. Malacol. Bull. 31:25–37.
Takeda, N., Y. Nakajima, O. Koizumi, T. Fujisawa, T. Takahashi, M. Matsumoto, and R. Deguchi. 2013. Neuropeptides trigger oocyte maturation and subsequent spawning in the hydrozoan jellyfish *Cytaeis uchidae*. Mol. Reprod. Dev. 80:223–232.
Tampi, P. R. S. 1960. On the early development of *Protula tubularia* (Montagu). J. Mar. Biol. Assoc. India 2:53–56.
Tampi, P., and K. Rengarajan. 1963. Occurrence of *Arenicola brasiliensis* Nonato (Fam. Arenicolidae, Polychaeta) in Indian waters. J. Mar. Biol. Assoc. India 5:108–112.
Tanner, R. L., L. E. Faye, and J. H. Stillman. 2019. Temperature and salinity sensitivity of respiration, grazing, and defecation rates in the estuarine eelgrass sea hare, *Phyllaplysia taylori*. Mar. Biol. 166:1–12. Springer Berlin Heidelberg.
Thiriot-Quiévreux, C. 1973. Heteropoda. Oceanogr. Mar. Biol. - An Annu. Rev. 11:237–261.
Thomas, F. I. M. 1994. Transport and mixing of gametes in three free-spawning polychaete annelids, *Phragmatopoma californica* (Fewkes), *Sabellaria cementarium* (Moore), and *Schizobranchia insignis* (Bush). J. Exp. Mar. Bio. Ecol. 179:11–27.
Thompson, T. E. 1962. Studies on the ontogeny of *Tritonia hombergi* Cuvier (Gastropoda Opisthobranchia). Philos. Trans. R. Soc. B Biol. Sci. 245:171–218.
Thompson, T. E., and U. Salghetti-Drioli. 1984. Unusual features of the development of the sacoglossan *Elysia hopei* in the Mediterranean Sea. J. Molluscan Stud. 50:61–63.
Thorson, G. 1946. Reproduction and larval development of Danish marine bottom invertebrates, with special reference to the planktonic larvae in the Sound (Öresund).
Thorson, G. 1950. Reproduction and larval ecology of marine bottom invertebrates. Biol. Rev. 25:1–45.
Thorson, G. 1940. Studies on the egg masses and larval development of Gastropoda from the Iranian Gulf. Danish Sci. Investig. Iran 2:159–238.
Thuesen, E. V., and J. J. Childress. 1993a. Enzymatic activities and metabolic rates of pelagic chaetognaths: Lack of depth-related declines. Limnol. Oceanogr. 38:935–948.
Thuesen, E. V., and J. J. Childress. 1993b. Metabolic rates, enzyme activities and chemical compositions of some deep-sea pelagic worms, particularly Nectonemertes mirabilis

(Nemertea; Hoplonemertinea) and Poeobius meseres (Annelida; Polychaeta). Deep. Res. Part I 40:937–951.
Todd, C. D., and J. N. Havenhand. 1988. Physiological ecology of Adalaria proxima (Alder et Hancock) and *Onchidoris muricata* (Müller) (Gastropoda: Nudibranchia). I. Freeding, growth, and respiration. J. Exp. Mar. Bio. Ecol. 118:191–205.
Todd, C., and R. Doyle. 1981. Reproductive strategies of marine benthic invertebrates: A settlement-timing hypothesis. Mar. Ecol. Prog. Ser. 4:75–83.
Tominaga, H., S. Nakamura, and M. Komatsu. 2004. Reproduction and development of the conspicuously dimorphic brittle star *Ophiodaphne formata* (Ophiuroidea). Biol. Bull. 206:25–34.
Tovar-Hernánde, M. A., B. Yáñez-Rivera, and J. L. Bortolini-Rosales. 2011. Reproduction of the invasive fan worm *Branchiomma bairdi* (Polychaeta: Sabellidae). Mar. Biol. Res. 7:710–718.
Tovar-Hernández, M. A. A., M. E. García-Garza, and J. A. de León-González. 2020. Sclerozoan and fouling sabellid worms (Annelida: Sabellidae) from Mexico with the establishment of two new species. Biodivers. Data J. 8:1–39.
Trevallion, A. 1971. Studies on *Tellina tenuis* Da Costa. III. Aspects of general biology and energy flow. J. Exp. Mar. Bio. Ecol. 7:95–122.
Trowbridge, C. D. 1995. Hypodermic insemination, oviposition, and embryonic development of a pool-dwelling ascoglossan (= sacoglosan) opisthobranch: *Ercolania feline* (Hutton, 1882) on New Zealand shores. Veliger 38:203–211.
Trowbridge, C. D. 1992. Phenology and demography of a marine specialist herbivore: *Placida dendritica* (Gastropoda: Opisthobranchia) on the central coast of Oregon. Mar. Biol. 114:443–452.
Trowbridge, C. D. 2000. The missing links: Larval and post-larval development of the ascoglossan opisthobranch *Elysia viridis*. J. Mar. Biol. Assoc. United Kingdom 80:1087–1094.
Trowbridge, C. D., Y. J. Hirano, and Y. M. Hirano. 2010. Sacoglossan opisthobranchs on Northwestern Pacific Shores: *Stiliger berghi* Baba, 1937, and *Elysia* sp. on filamentous red algae. Veliger 51:43–62.
Tsutsumi, H., and T. Kikuchi. 1984. Study of the life history of *Capitella capitata* (Polychaeta: Capitellidae) in Amakusa, South Japan including a comparison with other geographical regions. Mar. Biol. 80:315–321.
Turner, R. L. 2013. *Echinaster*. Pp. 201–214 *in* J. M. Lawrence, ed. Starfish: Biology and Ecology of the Asteroidea. Johns Hopkins University Press.
Turner, S. 1992. The egg capsules and early life history of the corallivorous gastropod *Drupella cornus* (Röding, 1798). Veliger 35:16–25.
Tutschulte, T. C. 1976. The Comparative Ecology of Three Sympatric Abalone. University of California, San Diego.
Twomey, M., E. Brodte, U. Jacob, U. Brose, T. P. Crowe, and M. C. Emmerson. 2012. Idiosyncratic species effects confound size-based predictions of responses to climate change. Philos. Trans. R. Soc. B Biol. Sci. 367:2971–2978.
Tyler, P. A., and J. D. Gage. 1984. The reproductive biology of echinothuriid and cidarid sea urchins from the deep sea (Rockall Trough, North-East Atlantic Ocean). Mar. Biol. 80:63–74.
Tyler, P. A., and J. D. Gage. 1982. The reproductive biology of *Ophiacantha bidenta* at a (Echinodermata: Ophiuroidea) from the Rockall Trough. J. Mar. Biol. Assoc. United Kingdom 62:45–55.
Tyler, P. A., and S. L. Pain. 1982. The reproductive biology of *Plutonaster bifrons*, *Dytaster insignis* and *Psilaster andromeda* (Asteroidea: Astropectinidae) from the Rockall

Trough. J. Mar. Biol. Assoc. United Kingdom 62:869–887. Monash University.
Tyler, P. A., S. L. Pain, and J. D. Gage. 1982. The reproductive biology of the deep-sea asteroid *Bathybiaster vexillifer*. J. Mar. Biol. Assoc. United Kingdom 62:57–69.
Tyler, P. A., S. L. Pain, J. D. Gage, and D. S. M. Billett. 1984. The reproductive biology of deep-sea forcipulate seastars (Asteroidea: Echinodermata) from the N.E. Atlantic Ocean. J. Mar. Biol. Assoc. United Kingdom 64:587–601.
Tyler, P. A., S. Reeves, L. Peck, A. Clarke, and D. Powell. 2003. Seasonal variation in the gametogenic ecology of the Antarctic scallop *Adamussium colbecki*. Polar Biol. 26:727–733.
Ulbricht, R. J., and A. W. Pritchard. 1972. Effect of temperature acclimation on the metabolic rate of sea urchins. Mar. Biol. 142:178–185.
Underwood, A. J. 1975. Comparative studies on the biology of *Nerita atramentosa* Reeve, *Bembicium nanum* (Lamarck) and *Cellana tramoserica* (Sowerby) (Gastropoda: Prosobranchia) in S.E. Australia. J. Exp. Mar. Bio. Ecol. 18:153–172.
Underwood, A. J. 1976. Food competition between age-classes in the intertidal neritacean *Nerita atramentosa* Reeve (Gastropoda: Prosobranchia). J. Exp. Mar. Bio. Ecol. 23:145–154.
Underwood, A. J. 1979. The ecology of intertidal gastropods. Adv. Mar. Biol. 16:111–210.
Uthicke, S. 1998. Respiration of *Holothuria* (*Halodeima*) *atra*, *Holothuria* (*Halodeima*) *edulis* and *Stichopus chloronotus*: Intact individuals and products of asexual reproduction. Pp. 531–536 *in* Mooi and Telford, eds. Echinoderms: San Francisco. Balkema, Rotterdam, Netherlands.
Vahl, O. 1972. Particle retention and relation between water transport and oxygen uptake in *Chlamys opercularis* (L.) (Bivalvia). Ophelia 10:67–74.
Vahl, O. 1978. Seasonal changes in oxygen consumption of the Iceland scallop (*Chlamys islandica* (O. F. Müller)) from 70°N. Ophelia 17:143–154.
Vahl, O., and J. H. Sundet. 1985. Is sperm really so cheap? Pp. 281–285 *in* J. S. Gray and M. E. Christainsen, eds. Marine biology of polar regions and effects of stress on marine organisms. John Wiley & Sons Ltd.
van der Land, J. 1975. Priapulida. Reprod. Mar. Invertebr. II:55–65.
Van Dover, C. L., J. Trask, J. Gross, and A. Knowlton. 1999. Reproductive biology of free-living and commensal polynoid polychaetes at the Lucky Strike hydrothermal vent field (Mid-Atlantic Ridge). Mar. Ecol. Prog. Ser. 181:201–214.
Van Soest, R. W. M. 2002. Family Agelasidae Verrill, 1907. Pp. 819–823 *in* J. N. A. Hooper and R. W. M. Van Soest, eds. Systema Porifera: A Guide to the Classification of Sponges. Kluwer Academic/Plenum Publishers, New York, USA.
Véliz, D., C. Guisado, and F. Winkler. 2001. Morphological, reproductive, and genetic variability among three populations of *Crucibulum quiriquinae* (Gastropoda: Calyptraeidae) in northern Chile. Mar. Biol. 139:527–534.
Véliz, D., F. M. Winkler, and C. Guisado. 2003. Developmental and genetic evidence for the existence of three morphologically cryptic species of *Crepidula* in northern Chile. Mar. Biol. 143:131–142.
Ventura, C. R. R. 2013. *Astropecten*. Pp. 101–108 *in* J. M. Lawrence, ed. Starfish: Biology and Ecology of the Asteroidea. Johns Hopkins University Press.
Verrill, A. E. 1914. Monograph of the shallow-water starfishes of the North Pacific Coast from the Arctic Ocean to California. Smithson. Inst. Publ. 2140 14:1–408.
Vial, M. V., R. W. Simpfendörfer, D. A. López, M. L. González, and K. Oelckers. 1992. Metabolic responses of the intertidal mussel *Perumytilus purpuratus* (Lamarck) in emersion and immersion. J. Exp. Mar. Bio. Ecol. 159:191–201.
Villalobos, F. B. 2005. Reproduction and larval biology of North Atlantic asteroids related to

the invasion of the deep sea. Univeristy of Southampton.
Villinski, J. T., J. C. Villinski, M. Byrne, and R. A. Raff. 2002. Convergent maternal provisioning and life-history evolution in echinoderms. Evolution. 56:1764.
Vuturo, S. A. 2004. The reproductive biology of *Ophiodromus pugettensis*. Northern Arizona University.
Walker, C. W., and M. P. Lesser. 1989. Nutrition and development of brooded embryos in the brittlestar *Amphipholis squamata*: do endosymbiotic bacteria play a role? Mar. Biol. 103:519–530.
Waller, R. G., P. A. Tyler, and J. D. Gage. 2005. Sexual reproduction in three hermaphroditic deep-sea *Caryophyllia* species (Anthozoa: Scleractinia) from the NE Atlantic Ocean. Coral Reefs 24:594–602.
Warnock, R. E., and W. D. Liddell. 1985. Oxygen consumption in two shallow-water comatulid crinoids. J. Exp. Mar. Bio. Ecol. 91:169–182.
Webb, C. M., and P. A. Tyler. 1985. Post-larval development of the common north-west European brittle stars Ophiura ophiura, O. albida and Acrocnida brachiata (Echinodermata: Ophiuroidea). Mar. Biol. 89:281–292.
Webber, H. H. 1977. Gastropoda: Prosobranchia. Pp. 1–97 *in* A. C. Giese and J. S. Pearse, eds. Reproduction of Marine Invertebrates: Molluscs: Gastropods and Cephalopods. Academic Press Inc., New York.
Webber, H. H., and A. C. Giese. 1969. Reproductive cycle and gametogenesis in the black abalone *Haliotis cracheroidii* (Gastropoda: Prosobranchiata). Mar. Biol. 4:152–159.
Weber, A. A. T., S. Stöhr, and A. Chenuil. 2014. Genetic data, reproduction season and reproductive strategy data support the existence of biological species in *Ophioderma longicauda*. Comptes Rendus - Biol. 337:553–560.
Webster, S. K. 1975. Oxygen consumption in echinoderms from several geographical locations, with particular reference to the Echinoidea. Biol. Bull. 148:157–164.
Webster, S. K., and A. C. Giese. 1975. Oxygen consumption of the purple sea urchin with special reference to the reproductive cycle. Biol. Bull. 148:165–180.
Weinrauch, A. M., and T. A. Blewett. 2019. Anoxia tolerance in the sea cucumbers *Parastichopus californicus* and *Cucumaria miniata* reflects habitat use. J. Exp. Mar. Bio. Ecol. 520:1–6. Elsevier.
Weis, V. M., E. A. Verde, A. Pribyl, and J. A. Schwarz. 2002. Aspects of the larval biology of the sea anemones *Anthopleura elegantissima* and *A. artemisia*. Invertebr. Biol. 121:190–201.
Welch, J. J. 2010. The "island rule" and deep-sea gastropods: Re-examining the evidence. PLoS One 5.
West, H., J. Harrigan, and S. Pierce. 1984. Hybridization of two populations of a marine opisthobranch with different developmental patterns. The Veliger 26:199–206.
Westheide, W. 1990. A hermaphroditic *Sphaerosyllis* (Polychaeta: Syllidae) with epitokous genital chaetae from intertidal sands of the Island of Phuket (Thailand). Can. J. Zool. 68:2360–2363.
Westheide, W. 1967. Die Gattung *Trilobodrilus* (Archiannelida, Polychaeta) von der deutschen Nordseeküste. Helgoländer Wissenschaftliche Meeresuntersuchungen 16:207–215.
Westheide, W. 1974. Interstituelle fauna von Galapagos. XI. Pisionidae, Pilargidae, Syllidae. Mikrofauna Meeresbodens 44:195–338.
Westheide, W. 1987. Systematics of the amphiatlantic *Microphthalmus-listensis*-species-group (Polychaeta: Hesionidae): Facts and concepts for reconstruction of phylogeny and speciation. J. Zool. Syst. Evol. Res. 25:12–39.
Westheide, W., and G. C. Rao. 1977. On some species of the genus Hesionides (Polychaeta,

Hesionidae) from Indian sandy beaches. Cah. Biol. Mar. 18:275–287.
Wheeling, R. J., E. A. Verde, and J. R. Nestler. 2007. Diel cycles of activity, metabolism, and ammonium concentration in tropical holothurians. Mar. Biol. 152:297–305.
Williams, J. D., and V. I. Radashevsky. 1999. Morphology, ecology, and reproduction of a new *Polydora* species from the east coast of North America (Polychaeta: Spionidae). Ophelia 51:115–127.
Wilson, D. P. 1936. Memoirs: The development of the sabellid *Branchiomma vesiculosum*. J. Cell Sci. s2-78:543–603.
Wilson, D. P. 1932. The development of *Nereis pelagica* Linnæus. J. Mar. Biol. Assoc. United Kingdom 18:203–217.
Wilson, D. P. 1929. The larvae of the British sabellarians. J. Mar. Biol. Assoc. United Kingdom 16:221–260.
Wilson, D. P. 1948. The larval development of *Ophelia bicornis* Savigny. J. Mar. Biol. Assoc. United Kingdom 27:540–553.
Wilson, D. P. 1933. The larval stages of *Notomastus latericeus* Sars. J. Mar. Biol. Assoc. United Kingdom 18:511–518. Monash University.
Wilson, N. G. 2002. Egg masses of chromodorid nudibranchs (Mollusca: Gastropoda: Opisthobranchia).
Wilson, S., J. Yeh, K. E. Korsmeyer, and J. C. Drazen. 2013. Metabolism of shallow and deep-sea benthic crustaceans and echinoderms in Hawaii. Mar. Biol. 160:2363–2373.
Wilson, W. H. 1983. Life-history evidence for sibling species in *Axiothella rubrocincta* (Polychaeta: Maldanidae). Mar. Biol. 76:297–300.
Wilson, W. H. 1991. Sexual reproductive modes in polychaetes: classification and diversity. Bull. Mar. Sci. 48:500–516.
Wisely, B. 1957. The development and settling of a serpulid worm, *Hydroides norvegica gunnerus* (polychaeta). Mar. Freshw. Res. 9:351–361.
Wolfowicz, I., S. Baumgarten, P. A. Voss, E. A. Hambleton, C. R. Voolstra, M. Hatta, and A. Guse. 2016. Aiptasia sp. larvae as a model to reveal mechanisms of symbiont selection in cnidarians. Sci. Rep. 6:1–12. Nature Publishing Group.
Wong, E., A. R. Davis, and M. Byrne. 2010. Reproduction and early development in *Haliotis coccoradiata* (Vetigastropoda: Haliotidae). Invertebr. Reprod. Dev. 54:77–87.
Woodwick, K. H. 1953. *Polydora nuchalis*, a new species of polychaetous annelid from California. J. Washingt. Acad. Sci. 43:381–383.
Worley, E. K., D. R. Franz, and G. Hendler. 1977. Seasonal patterns of gametogenesis in a North Atlantic brooding asteroid, *Leptasterias tenera*. Biol. Bull. 153:237–253.
Worsaae, K., A. Kerbl, Á. Vang, and B. C. Gonzalez. 2019. Broad North Atlantic distribution of a meiobenthic annelid – against all odds. Sci. Rep. 9:1–13.
Worsfold, T. 2009. Identification of Cirratulidae in British and Irish waters. Prog. Identif. Cirratulidae Br. Irish Waters Through NMBAQC Scheme 1996-2009 1–114.
Wray, G. A., and R. A. Raff. 1991. The evolution of developmental strategy in marine invertebrates. Trends Ecol. Evol. 6:45–50.
Wyatt, H. V. 1960. Duration of embryonic life of *Calyptraea chinensis* (L.) (Mollusca). Ann. Mag. Nat. Hist. 3:333–335.
Yamaguchi, M. 1975. Coral-reef asteroids of Guam. Biotropica 7:12–23.
Yamaguchi, M. 1973. Early life histories of coral reef asteroids, with special reference to *Acanthaster planci* (L.). Pp. 369–387 *in* A. Jones and R. Endean, eds. Biology and Geology of Coral Reefs. Academic Press, New York.
Yamaguchi, M. 1974. Larval life span of the coral reef asteroid *Gomophia egyptiaca*. Micronesica 10:57–64.
Yamaguchi, M., and J. S. Lucas. 1984. Natural parthenogenesis, larval and juvenile

development, and geographical distribution of the coral reef asteroid *Ophidiaster granifer*. Mar. Biol. 83:33–42.
Yamashita, M. 1983. Electron microscopic observations during monospermic fertilization process of the brittle-star *Amphipholis kochii* Lütken. J. Exp. Zool. 228:109–120.
Yamashita, M. 1985. Embryonic development of the brittle-star *Amphipholis kochii* in laboratory culture. Biol. Bull. 169:131–142.
Yamazaki, D., O. Miura, M. Ikeda, A. Kijima, D. Van Tu, T. Sasaki, and S. Chiba. 2017. Genetic diversification of intertidal gastropoda in an archipelago: the effects of islands, oceanic currents, and ecology. Mar. Biol. 164:164–184.
Yeruham, E., A. Abelson, G. Rilov, D. Ben Ezra, and M. Shpigel. 2019. Energy budget of cultured *Paracentrotus lividus* under different temperatures. Aquaculture 501:7–13.
Yonow, N. 1996. Gametogenesis, egg production and development in *Acteon tornatilis* (Opisthobranchia: Cephalaspidea). Malacol. Rev. Molluscan Reprod. Supplement:31–52.
Yoshiwara, S. 1898. Preliminary notice of new Japanese echinoids. Annot. Zool. Jpn. 2:57–61.
Young, C. M. 2003. Reproduction, development, and life history traits. Pp. 381–426 *in* P. A. Tyler, ed. Ecosyst. World.
Young, C. M., S. M. Arellano, J. F. Hamel, and A. Mercier. 2018. Ecology and evolution of larval dispersal in the deep sea. Pp. 229–250 *in* T. J. Carrier, A. M. Reitzel, and A. Heyland, eds. Evolutionary Ecology of Marine Invertebrate Larvae. 1. C. M. Young, S. M. Arellano, J. F. Hamel, A. Mercier, in Evolutionary Ecology of Marine Invertebrate Larvae (2018), pp. 229–250.
Young, C. M., and F.-S. Chia. 1982. Ontogeny of phototaxis during larval development of the sedentary Polychaete, *Serpula vermicularis* (L.). Biol. Bull. 162:457–468.
Young, C. M., and S. B. George. 2000. Larval development of the tropical deep-sea echinoid *Aspidodiadema jacobyi*: Phylogenetic implications. Biol. Bull. 198:387–395.
Yu, Z., Z. Qi, C. Hu, W. Liu, and H. Huang. 2013. Effects of salinity on ingestion, oxygen consumption and ammonium excretion rates of the sea cucumber *Holothuria leucospilota*. Aquac. Res. 44:1760–1767.
Yukihira, H., D. W. Klumpp, and J. S. Lucas. 1998. Effects of body size on suspension feeding and energy budgets of the pearl oysters *Pinctada margaritifera* and *P. maxima*. Mar. Ecol. Prog. Ser. 170:119–130.
Yun, S. G., and T. Kikuchi. 1991. Larval development and settlement of *Chone duneri* Malmgren (Polychaeta: Sabellidae). Publ. from Amakusa Mar. Biol. Lab. Kyushu Univ. 11:31-42,illustr.
Zal, F., D. Jollivet, P. Chevaldonné, and D. Desbruyères. 1995. Reproductive biology and population structure of the deep-sea hydrothermal vent worm *Paralvinella grasslei* (Polychaeta: Alvinellidae) at 13°N on the East Pacific Rise. Mar. Biol. 122:637–648.
Zardus, J. D. 2002. Protobranch bivalves. Adv. Mar. Biol. 42:1–65.
Zawierucha, K., K. Grzelak, L. Kotwicki, Ł. Kaczmarek, and R. M. Kristensen. 2015. First observation of the marine tardigrades *Batillipes mirus* and *Batillipes noerrevangi* (Arthrotardigrada, Batillipedidae) from a strongly brackish part of the Polish Baltic Sea coast. Mar. Biol. Res. 11:859–868.
Zeuthen, E. 1947. Body size and metabolic rate in the animal kingdom with special regard to the marine microfauna. C. R. Trav. Lab. Carlsberg 26:17–161.
Zhadan, A. E., E. V. Vortsepneva, and A. B. Tsetlin. 2012. Redescription and biology of *Cossura pygodactylata* Jones, 1956 (Polychaeta: Cossuridae) in the White Sea. Invertebr. Zool. 9:115–125.
Ziv, I., S. Markovich, C. Lustig, and A. J. Susswein. 1991. Effects of food and mates on time

budget in *Aplysia fasciata*: Integration of feeding, reproduction, and locomotion. Behav. Neural Biol. 55:68–85.

Zottoli, R. A. . 1974. Reproduction and larval development of the ampharetid polychaete *Amphicteis floridus*. Trans. Am. Microsc. Soc. 93:78–89.
