# Supplementary figures and images for: Macroevolutionary patterns in marine hermaphroditism
Source: Evolution. 2022 Oct 13;76(12):3014–25. doi: 10.1111/evo.14639 (PMC10091813; doi:10.1111/evo.14639)

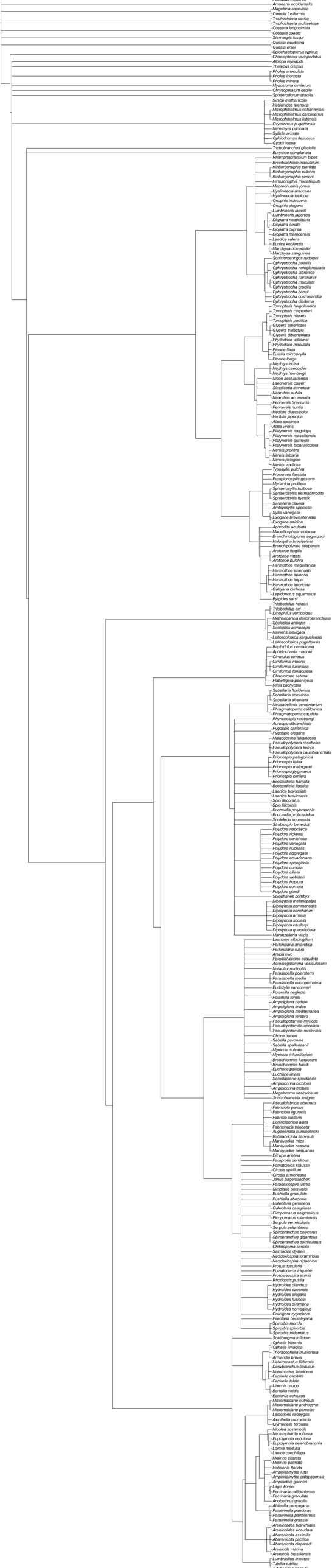

Supplement: Supplementary file 3 [file EVO-76-3014-s001.zip › raw_data_references_trees/Annelid_tree.pdf]

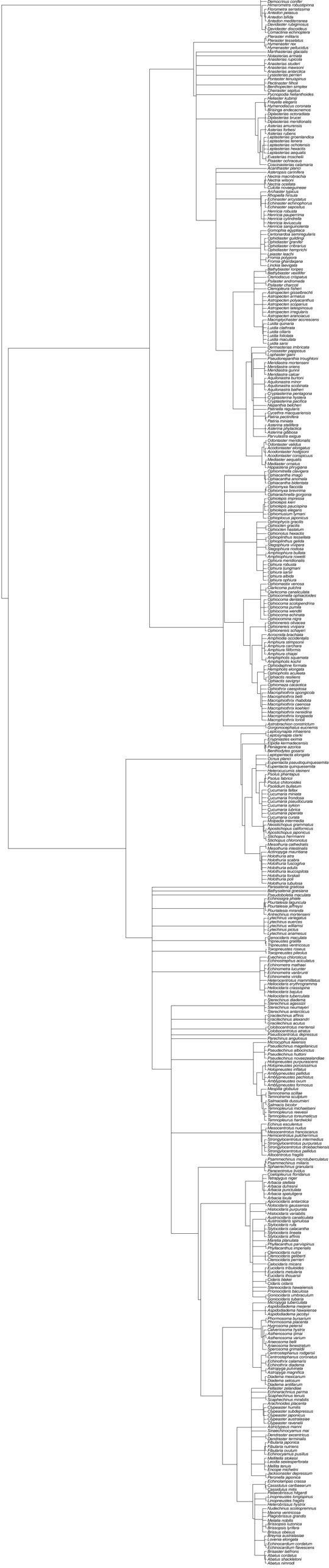

Supplement: Supplementary file 3 [file EVO-76-3014-s001.zip › raw_data_references_trees/Echinoderm_tree.pdf]

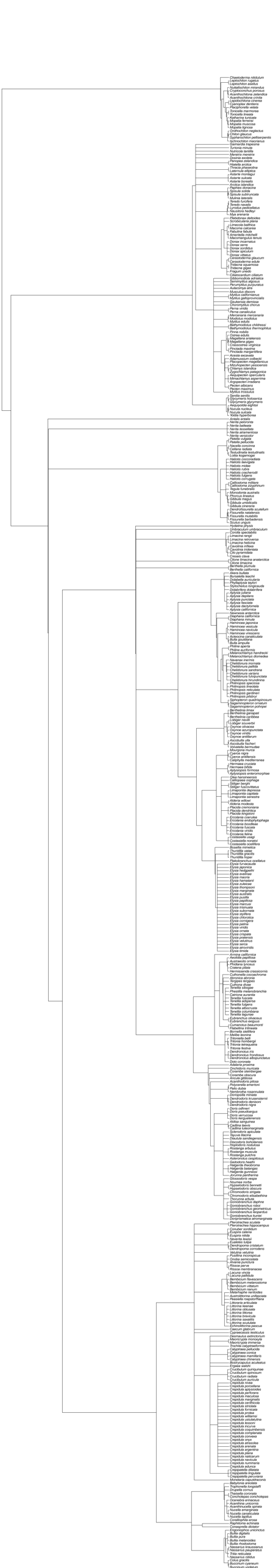

Supplement: Supplementary file 3 [file EVO-76-3014-s001.zip › raw_data_references_trees/Mollusc_tree.pdf]
